# Antimicrobial susceptibility and molecular characteristics of *Mycoplasma pneumoniae* isolates from children in Xi'an regions of China

**DOI:** 10.3389/fped.2025.1660687

**Published:** 2025-10-15

**Authors:** Chao Huang, Jun Yang, Jia Cao, Haiyan Ren, Nan Gao, Jianjun Liu

**Affiliations:** ^1^ Xi’an International Medical Center Hospital, Xi’an, China; ^2^ Xi’an Gaoxin Hospital, Xi’an, China

**Keywords:** *mycoplasma pneumoniae*, MALDI-TOF MS, P1-I, children, antibiotic resistance

## Abstract

**Introduction:**

Mycoplasma pneumoniae (MP) is a primary cause of pediatric pneumonia, with rising macrolide resistance being a global concern. Data on its molecular epidemiology and resistance patterns in Xi'an, China, are limited. This study aimed to characterize MP isolates from children in Xi'an to inform local treatment strategies.

**Methods:**

In 2023, throat swabs were collected from 376 children hospitalized with MP infection. From these, 70 MP isolates were cultured. Antimicrobial susceptibility testing was performed, and isolates were genotyped using PCR-MALDI-TOF MS to identify P1 types and mutations in the 23S rRNA gene associated with macrolide resistance.

**Results:**

The MP culture-positive rate was 18.61% (70/376), with the highest prevalence in school-aged children (5–14 years, 81%). The P1-I genotype was predominant (97%). All 70 isolates harbored A2063G, A2064T, and A2617C mutations in the 23S rRNA gene. However, phenotypic resistance was 38.6% for macrolides, 31.4% for quinolones, and 38.6% for tetracyclines, indicating a significant genotype-phenotype discordance for macrolides.

**Conclusion:**

MP strains in Xi'an show high rates of genotypic macrolide resistance and a predominance of the P1-I genotype. The notable discordance between the universal presence of resistance mutations and observed phenotypic resistance underscores the importance of integrating both molecular and culture-based susceptibility testing to guide effective clinical management.

## Introduction

*Mycoplasmas*, diminutive self-replicating entities devoid of cell walls, comprise more than 200 species distributed among plants, animals, arthropods, and humans. Certain *Mycoplasmas* are implicated in human maladies, among which *Mycoplasma pneumoniae*(*M. pneumoniae*) stands as a thoroughly investigated specimen (1). This particular strain is notorious for instigating both upper and lower respiratory tract afflictions in individuals of all ages (2, 3). It accounts for approximately 10%–50% of cases of pediatric community-acquired pneumonia (4). Moreover, *M. pneumoniae* has been implicated in various extrapulmonary disorders, encompassing encephalitis, dermatological manifestations, and septic arthritis (2, 3).

In 1968, Niitu et al. (4) in Japan initially isolated multidrug-resistant *M. pneumoniae* (MP) from a young girl undergoing treatment with erythromycin for pneumonia. Subsequently, Critchley et al. (5) isolated two strains resistant to macrolide antibiotics between 1995 and 1999. Presently, drug resistance rates are on the rise worldwide, with Northeast Asia (China, Japan, and South Korea) grappling with the most pronounced challenge (6). Significant disparities in drug resistance rates exist across different regions of China, with Beijing reporting the highest incidence and resistance ratio (7). The macrolide(ML) antibiotic is commonly prescribed for *M. pneumoniae* infections, yet since 2000, the resistance rate to macrolide antibiotics has been steadily escalating. East Asian nations, notably China, exhibit relatively elevated resistance rates compared to European and American counterparts, where resistance rates are lower (8). Japan experienced an initial surge followed by a decline in resistance rates, with the highest recorded rate ranging from 2010 to 2015, reaching 70.7% (9).

Molecular characterization assumes paramount significance in the surveillance of *M. pneumoniae* infection epidemiology. To enhance reproducibility and comparability, this study now provides detailed information about the molecular typing methodology used, including the specific SNPs(single nucleotide polymorphisms) analyzed. The sequences reported have yet to be deposited in a DNA sequence database, a limitation acknowledged here with a recommendation for future studies to use public repositories like GenBank. Diverse genotyping methodologies have been devised for this endeavor, facilitating the exploration of *M. pneumoniae* 's molecular biology and epidemiological attributes. The genome of *M. pneumoniae* exhibits remarkable conservation, with sequence homogeneity among distinct strains reaching a staggering 99%. This bacterium is classified into genotype I and genotype II based on SNPs (10). In recent years, matrix-assisted laser desorption/ionization time-of-flight mass spectrometry (MALDI-TOF MS) has emerged as a prevalent tool for microbial detection and analysis, heralded as the new benchmark for identifying various microorganisms (11). SNP analysis utilizing MALDI-TOF mass spectrometry confers advantages such as high-throughput capability, rapid detection, and concurrent analysis of multiple targets, rendering it a propitious biomolecular technology (12).

Although the majority of *M. pneumoniae*-related investigations in mainland China have centered on Beijing and Shanghai, scant research has explored other regions (13–18). Notably, prior studies examining *M. pneumoniae* genotypes and antimicrobial resistance have predominantly relied on *M. pneumoniae* DNA detection rather than clinical isolates. This study underscores the analysis of genotypes and antimicrobial drug susceptibility in 70 clinical isolates of *M. pneumoniae* gathered in Xi'an in 2023.

## Material and methods

### M. pneumoniae strains

A cohort of 376 children diagnosed with *M. pneumoniae* infection were admitted to the pediatric department of our hospital. The study comprised 205 males (54.52%) and 171 females (45.47%). Specimens were procured from throat swabs and subsequently clinically isolated, purified, and cultured for *M. pneumoniae*.

### Instruments

The study employed an array of instruments and reagents, including a PCR instrument from Therm Company, a high-speed centrifuge from Shanghai Analytical Instrument Factory, *M. pneumoniae* detection kits from Antu Biotechnology and Fuji Rebio, Taq mixDNA Polymerase and associated products from Sangon Bioengineering, MALDI-TOF MS QUABTOF from Qingdao Rongzhi Biotechnology, and antibiotics sourced from the British Oxoid Company.

### Culture and isolation of *M. pneumoniae*

Clinical throat swab specimens were immediately inoculated into PPLO broth, a nutrient-rich transport/culture medium supplemented with ∼20% horse serum, yeast extract, antibiotics, glucose, and a phenol red pH indicator (19). Cultures were incubated at 37°C with 5% CO₂ and high humidity for 2–3 weeks, as recommended by CLSI guidelines. Due to the slow growth and lack of visible turbidity of *M. pneumoniae*, cultures were monitored by color change (red to yellow), indicating acid production from glucose metabolism (20). Positive broths were subsequently subcultured onto solid PPLO agar and incubated for an additional 1–2 weeks under identical conditions. Colonies were examined microscopically at low magnification for the characteristic “fried-egg” appearance (small, 100–300 µm diameter colonies) before further identification (21).

### Drug sensitivity and MIV test

A drug sensitivity test is conducted to ascertain the tolerance levels of *M. pneumoniae* to various antibiotics, including Erythromycin Estolate, erythromycin, minocycline, doxycycline, azithromycin, josamycin, acetylspiramycin, clindamycin, clarithromycin, roxithromycin, ciprofloxacin, moxifloxacin, levofloxacin, and gatifloxacin, utilizing a multiple dilution method.

We performed antimicrobial susceptibility testing and MIC assays using the *M. pneumoniae* culture and susceptibility test kit (Autobio Diagnostics Co., Ltd). The culture medium and antimicrobial susceptibility test plate were prepared near the front of the workspace before sample collection. After opening the culture medium bottle, 100 µl of the culture medium was transferred using a pipette tip into the negative control well (C- well). The throat swab (or 100 µl sputum swab/sputum sample) was then added to the remaining culture medium, capped, and mixed thoroughly by shaking. Subsequently, 100 µl of the sample-containing culture medium was dispensed into each of the remaining wells, and the susceptibility test plate was gently agitated to ensure even distribution. One drop of the kit-provided mineral oil was added to each well. The plate was then covered, placed into an incubator, and cultured at 36–38°C for 24–48 h, after which results were observed. The reference strain was ATCC 15377.

### PCR-MALDI-TOF Ms

Nucleotide identification of each purified isolate was confirmed using PCR-MALDI-TOF MS, as outlined in a previous study (22). Nucleic acids extracted from 70 *M. pneumoniae* strains (ranging from 12 to 83 ng/μl, quantified using Qubit 3.0) were utilized (refer to Supplementary Table S1). Nucleic acid-free water served as the negative control. For multiplex PCR amplification, a total volume of 5 µl was prepared, comprising 1 µl of DNA template, 2.0 µl of PCR buffer (Intelligene Biosystems, Qingdao, China), and 1.0 µl of PCR primer mixture. The amplification conditions were as follows: initial denaturation at 95°C for 15 min, followed by 30 cycles of denaturation at 95°C for 15 s, annealing at 59°C for 30 s, and extension at 72°C for 30 s Subsequently, the PCR products underwent treatment with shrimp alkaline phosphatase (SAP) to eliminate free dNTPs. Each PCR tube was then supplemented with 2 μl of SAP (Intelligene Biosystems, Qingdao, China), and the mixture underwent PCR amplification under the following conditions: 37°C for 40 min, followed by 85°C for 5 min, with maintenance at 4°C. For mass probe extension (MPE), a 4 µl buffer solution was prepared, comprising 1 µl E-ddNTP, 1.4 µl MPE buffer, 0.6 µl MPE enzyme, and 1.0 µl primer. To each tube containing SAP-treated products, 4 μl of the MPE mixture was added and vortexed. The PCR conditions for MPE were as follows: 95°C for 30 min, followed by denaturation at 95°C for 5 s, 5 cycles of annealing at 52°C for 5 s and extension at 80°C for 5 s, 40 cycles of denaturation at 95°C for 5 s, followed by 5 cycles, and a final extension at 72°C for 3 min. Subsequently, single base pair extension was performed. For salt purification, 14 μl of ultrapure water and resin were added to each well, followed by vortexing for 30 min. The supernatant was harvested for testing post-centrifugation. For SNP identification and subsequent data analysis via MALDI-TOF MS, 3-hydroxypyridine-2-carboxylic acid (3-HPA, 0.9 μl) was applied at the center of the sample target. Upon drying, the matrix was overlaid with 0.3μl of purified supernatant. Following this, the samples underwent testing post-crystallization. Data collection was executed utilizing the QuanTOF I system (Intelligene Biosystems, Qingdao, China) under the specified parameters: positive ion data acquisition mode across a mass range of 4,000–9,000 Da. The focus mass was set at 4,000 Da, with a pulse frequency of 2000Hz. The detector voltage was −0.53 kV, and the extraction voltage was −3.45 kV. Laser pulse energy remained constant at 25 μJ. Subsequent analysis of the sample extension results was conducted employing the QuanSNP system (V1.0, IntelliBio, China).

## Results

### Incidence of *M.pneumoniae*

A total of 376 samples were collected from pediatric patients for testing, revealing that 70 cases of *M. pneumoniae* were cultured, resulting in a positive rate of 18.61%. The pediatric patients were grouped based on age and gender, divided into the following age groups: <1 year old, 1–3 years old, 3–5 years old, and 5–14 years old. Among the 376 pediatric patients tested, the majority of *M. pneumoniae*-positive cases were identified in school-age children (5–14 years old), accounting for approximately 81% of positive isolates (57 out of 70 cases). In contrast, significantly fewer infections were observed in younger age groups, with only 12 cases (17.1%) among children aged 3–5 years and one positive case (1.4%) in the 1–3 years group. No infections were detected in infants under one year of age. This age distribution suggests higher susceptibility or exposure risk among older children. It was observed that there were more children over 5 years old compared to those under 5 years old. Among the *M. pneumoniae* positive children, there were 37 males and 33 females. Please refer to Table 1 for further details.

**Table 1:** Incidence of M.*pneumoniae* by age and gender.

Age	Male			Female			Total		
	No.	MP positive	Positive rate	No.	MP positive	Positive rate	No.	MP positive	Positive rate
<1	1	0	0%	0	0	0%	1	0	0%
1∼3	5	1	20%	1	0	0%	6	1	16.66%
3∼5	40	6	15%	25	6	24%	65	12	18.46%
5∼14	159	30	18.87%	145	27	18.62%	304	57	18.69%

Age groups: <1 year old, 1–3 years old, 3–5 years old, and 5–14 years old.

### Antimicrobial susceptibility testing of *M. pneumoniae*

Resistance rates among the 70 isolates varied across different macrolide antibiotics tested individually: erythromycin (20%), azithromycin (12.9%), clarithromycin (12.9%), josamycin (12.9%), midecamycin (18.6%), roxithromycin (17.1%), spiramycin (12.9%), and acetylspiramycin (11.4%). Resistance rates to quinolone antibiotics were: ciprofloxacin (25.7%), levofloxacin (30%), moxifloxacin (21.4%), and ofloxacin (20%). Additionally, the resistance rates of *M. pneumoniae* to tetracycline antibiotics exceed 35%. For tetracyclines, resistance rates were 8.6% for tetracycline and 30% for doxycycline.For further details, please consult Table 2, Supplementary Tables S2 and S3.

**Table 2:** M.*pneumoniae* drug sensitivity results.

Drug	Drug resistance		Intermediary		Sensitive	
	No.	Ratio	No.	Ratio	No.	Ratio
Macrolide	27	38.57%	11	15.71%	32	45.71%
Erythromycin Estolate	14	20%	1	1.43%	55	78.57%
Erythromycin	9	12.86%	6	8.57%	55	78.57%
Azithromycin	9	12.86%	6	8.57%	55	78.57%
Josamycin	9	12.86%	14	20%	47	67.14%
Acetylspiramycin	13	18.57%	9	12.86%	48	68.57%
Clindamycin	12	17.14%	15	21.43%	43	61.43%
Clarithromycin	9	12.86%	6	8.57%	55	78.57%
Roxithromycin	8	11.43%	5	7.14%	57	81.43%
Quinolone	22	31.43%	24	34.28%	24	34.28%
Ciprofloxacin	18	25.71%	19	27.14%	33	47.15%
Moxifloxacin	21	30%	11	15.71%	38	54.29%
Levofloxacin	15	21.43%	15	21.43%	40	57.14%
Gatifloxacin	14	20%	15	21.43%	41	58.57%
Tetracycline	27	38.57%	30	42.86%	13	18.57%
Minocycline	6	8.57%	23	32.86%	41	58.57%
Doxycycline	21	30%	30	42.86%	19	27.14%

Antimicrobial susceptibility test including Macrolide, Quinolone and Tetracycline.

### Nucleic acid detection of *M. pneumoniae* isolates

**Figure 1:**
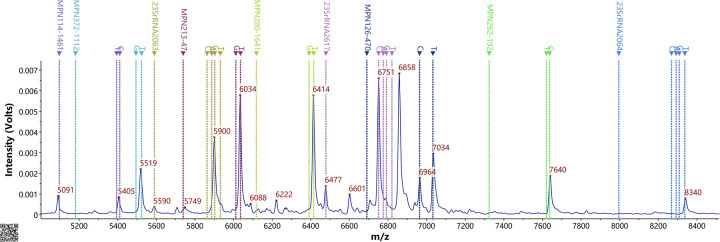
Ms peak of the MPE probes. Detection of 9 SNP sites in the presence of nucleic acid from *M. pneumoniae* based on the PCR-MALDI-TOF MS method.

Multiplex PCR coupled with MALDI-TOF MS assay was employed to streamline the detection of the nine SNPs, thereby obviating the need for sequencing gene fragments. Consequently, the SNP of this site served as the foundation of the complementary strand (see Table 3, Figure 1). MALDI-TOF MS-based amplification and identification were conducted on *M. pneumoniae* strains. Six SNP genotyping sites and three ML susceptibility gene sites were discerned, with no MS peak observed in the blank control. There existed complete concordance between the MS SNP results of the three ML susceptibility sites and the sequencing outcomes of the strains. Among the 70 strains, four SNP types (0, 11, 17, and 27) were identified. Sixty-eight odd-numbered SNP types were categorized into genotype I, while two even-numbered SNP types were classified into genotype II (refer to Table 3). For instance, in Figure 1, there were nine MS peaks for *M. pneumoniae*, representing six SNP typing mass probes and three ML susceptibility mass probes. Therefore, the predominant type of *M. pneumoniae* in Xi'an is P1 type 1.

**Table 3:** Genotyping and macrolide susceptibility results of M. *pneumoniae* strains used in this study.

Sample	MP						23S rRNA			SNP type	Geno type
	N114^1461^	N126^470^	N213^47^	N262^192^	N280^1641^	N372^1112^	2063	2064	2617		
	C>T	C>T	G>T	C>T	G>T	G>T	A>G,C,T	A>G,C,T	A>G,C,T		
1	C	C,T	T	C	T	T	G	T	C	11/17	Ⅰ
2	C	C	T	C	T	T	G	T	C	11	Ⅰ
3	C	T	T	C	T	T	G	T	C	27	Ⅰ
4	C	C,T	T	C	T	T	G	T	C	11/17	Ⅰ
5	C	C,T	T	C	T	T	G	T	C	11/17	Ⅰ
6	C	T	T	C	T	T	G	T	C	27	Ⅰ
7	C	C,T	T	C	T	T	G	T	C	11/17	Ⅰ
8	C	C	T	C	T	T	G	T	C	11	Ⅰ
9	C	C	T	C	T	T	G	T	C	11	Ⅰ
10	C	C,T	T	C	T	T	G	T	C	11/17	Ⅰ
11	C	T	T	C	T	T	G	T	C	27	Ⅰ
12	C	C,T	T	C	T	T	G	T	C	11/17	Ⅰ
13	C	T	T	C	T	T	G	T	C	27	Ⅰ
14	C	T	T	C	T	T	G	T	C	27	Ⅰ
15	C	C	G	C	G	G	G	T	C	0	Ⅱ
16	C	C	T	C	T	T	G	T	C	11	Ⅰ
17	C	T	T	C	T	T	G	T	C	27	Ⅰ
18	C	T	T	C	T	T	G	T	C	27	Ⅰ
19	C	T	T	C	T	T	G	T	C	27	Ⅰ
20	C	T	T	C	T	T	G	T	C	27	Ⅰ
21	C	C,T	T	C	T	T	G	T	C	11/17	Ⅰ
22	C	T	T	C	T	T	G	T	C	27	Ⅰ
23	C	T	T	C	T	T	G	T	C	27	Ⅰ
24	C	T	T	C	T	T	G	T	C	27	Ⅰ
25	C	T	T	C	T	T	G	T	C	27	Ⅰ
26	C	T	T	C	T	T	G	T	C	27	Ⅰ
27	C	T	T	C	T	T	G	T	C	27	Ⅰ
28	C	T	T	C	T	T	G	T	C	27	Ⅰ
29	C	T	T	C	T	T	G	T	C	27	Ⅰ
30	C	T	T	C	T	T	G	T	C	27	Ⅰ
31	C	T	T	C	T	T	G	T	C	27	Ⅰ
32	C	C	T	C	T	T	G	T	C	11	Ⅰ
33	C	T	T	C	T	T	G	T	C	27	Ⅰ
34	C	C,T	T	C	T	T	G	T	C	11/17	Ⅰ
35	C	T	T	C	T	T	G	T	C	27	Ⅰ
36	C	T	T	C	T	T	G	T	C	27	Ⅰ
37	C	C	G,T	C	G,T	T,G	G	T	C	0	Ⅱ
38	C	T	T	C	T	T	G	T	C	27	Ⅰ
39	C	T	T	C	T	T	G	T	C	27	Ⅰ
40	C	T	T	C	T	T	G	T	C	27	Ⅰ
41	C	T	T	C	T	T	G	T	C	27	Ⅰ
42	C	T	T	C	T	T	G	T	C	27	Ⅰ
43	C	C,T	T	C	T	T	G	T	C	11/17	Ⅰ
44	C	T	T	C	T	T	G	T	C	27	Ⅰ
45	C	T	T	C	T	T	G	T	C	27	Ⅰ
46	C	T	T	C	T	T	G	T	C	27	Ⅰ
47	C	C,T	T	C	T	T	G	T	C	11/17	Ⅰ
48	C	C,T	T	C	T	T	G	T	C	11/17	Ⅰ
49	C	T	T	C	T	T	G	T	C	27	Ⅰ
50	C	T	T	C	T	T	G	T	C	27	Ⅰ
51	C	T	T	C	T	T	G	T	C	27	Ⅰ
52	C	T	T	C	T	T	G	T	C	27	Ⅰ
53	C	T	T	C	T	T	G	T	C	27	Ⅰ
54	C	T	T	C	T	T	G	T	C	27	Ⅰ
55	C	T	T	C	T	T	G	T	C	27	Ⅰ
56	C	T	T	C	T	T	G	T	C	27	Ⅰ
57	C	T	T	C	T	T	G	T	C	27	Ⅰ
58	C	T	T	C	T	T	G	T	C	27	Ⅰ
59	C	T	T	C	T	T	G	T	C	27	Ⅰ
60	C	T	T	C	T	T	G	T	C	27	Ⅰ
61	C	T	T	C	T	T	G	T	C	27	Ⅰ
62	C	T	T	C	T	T	G	T	C	27	Ⅰ
63	C	T	T	C	T	T	G	T	C	27	Ⅰ
64	C	T	T	C	T	T	G	T	C	27	Ⅰ
65	C	T	T	C	T	T	G	T	C	27	Ⅰ
66	C	T	T	C	T	T	G	T	C	27	Ⅰ
67	C	T	T	C	T	T	G	T	C	27	Ⅰ
68	C	T	T	C	T	T	G	T	C	27	Ⅰ
69	C	T	T	C	T	T	G	T	C	27	Ⅰ
70	C	T	T	C	T	T	G	T	C	27	Ⅰ

Six SNP genotyping sites and three ML susceptibility gene sites were detected.

For the ML susceptibility sites, all 70 strains were confirmed to possess the A2063G mutation, 70 strains with the A2064T mutation, and 70 strains with the A2617C mutation. However, the MS findings were incongruent with the results of drug sensitivity tests.

Locations of key macrolide-resistance mutations in the domain V region of the *M. pneumoniae* 23S rRNA. A2063 and A2064 reside in the central loop of domain V, while position 2617 is nearby on an adjacent stem-loop. All of these mutations map to the peptidyl-transferase loop of 23S rRNA, which is the macrolide binding site, explaining their impact on drug affinity.

## Discussion

This study presents the latest epidemiological insights into *M. pneumoniae* at Xi'an Hospital in China. The data elucidates that the P1–1 genotype remains predominant among *M. pneumoniae* strains in Xi'an, with primary SNP types identified as 0, 11, 17, and 27. The prevalence of the P1 genotype of *M. pneumoniae* demonstrates variability across different regions and over time. A study conducted by Dumke et al. (23) revealed that between 2003 and 2006, the distribution of P1 genotypes in *M. pneumoniae* isolates from Germany was 31% for P1 I, 35% for P1 II, and 34% for the variant strains P1-IIa and P1-IIb. Similarly, research by Martínez et al. (24) demonstrated that in Chile between 2005 and 2006, the distribution of P1 genotypes in *M. pneumoniae* isolates was 21.7% for P1 I and 78.3% for P1 II. In Beijing, the primary genotype of *M. pneumoniae* from 2008 to 2012 was type I (25) but in 2013, there was an increase in the proportion of genotype II *M. pneumoniae* (26). From 2014 to 2016, the proportion of genotype II *M. pneumoniae* ranged from 30% to 40% (27). Wang et al. (28) reported that in the Shanghai region, 166 strains of *M. pneumoniae* were predominantly classified as P1 I, with only 16 strains identified as P1 II. Notably, an observed phenomenon of alternating epidemic transitions between different types (P1 I and P1 II) was noted (29).

A significant finding of this study was the age distribution of *M. pneumoniae* infections, with school-age children (5–14 years old) exhibiting the highest positivity rate, accounting for approximately 81% (57 out of 70) of the positive isolates. This susceptibility in older children can be attributed to several factors, including their developing immune systems and increased exposure in densely populated environments such as schools and kindergartens, which facilitate the spread of respiratory illnesses. Our findings are consistent with previous literature; for example, Ken et al. (2) observed the highest infection rates among children aged 4–6 years, and high incidence rates have been noted among school-age children in the United States (30, 31). When comparing our results with studies from Iran, a nuanced picture emerges (32). While Iranian studies generally report *M. pneumoniae* as causing a minority of pediatric respiratory infections—a 2019 meta-analysis found a pooled prevalence of about 9% and a specific PCR-based study in Tehran (2014–2015) detected it in only 4.7% of cases —our study's higher positivity rate in Xi'an (∼18.6%) aligns with the higher end of the reported Iranian range (32, 33). This difference may reflect variations in the age of the pediatric sample, active epidemic conditions, and detection methodologies, as the Tehran study grew no isolates by culture. Globally, Northeast Asia has reported higher *M. pneumoniae* incidence in children during epidemic years. Supporting the age effect, a Tehran seroepidemiology survey found that 25.1% of asymptomatic children aged 5–6 already possessed IgG antibodies against *M. pneumoniae*, indicative of past exposure, even without acute infection at the time of the survey (34). This suggests that many infections occur by early school age, often mildly or subclinically, which is consistent with our finding that most cases in Xi'an occurred in children over 5 years old (32). Thus, while prevalence varies, a consistent pattern of higher infection rates in susceptible older children is observed, with our Xi'an data confirming a significant burden in this demographic.

The antimicrobial resistance landscape in Xi'an presents a considerable challenge. Our study revealed that *M. pneumoniae* isolates exhibited resistance rates exceeding 30% to commonly used macrolide, quinolone, and tetracycline antibiotics. Specifically, overall macrolide resistance was 38.57%, quinolone resistance was 31.43%, and tetracycline resistance was 38.57%. These findings are based on antimicrobial susceptibility testing methodology updated according to CLSI(Clinical and Laboratory Standards Institute) guidelines (2011) for human mycoplasmas, incorporating MIC(minimum inhibitory concentrations)50, MIC90 values, control strains, and MIC breakpoints for robust interpretation.

These local resistance patterns are situated within a global context of rising macrolide resistance in *M. pneumoniae*. Furthermore, a delayed resurgence of *M. pneumoniae* infections has been noted post-COVID-19, with significant impacts reported in China, as detailed in recent studies by Meyer Sauteur et al. (35) and Chen et al. (36). Our 2023 data provides a critical, timely snapshot of the resistance landscape during this post-pandemic resurgence, highlighting the challenges clinicians now face. Comparing resistance rates internationally, Japan reported a rate of 17.1% in 2004 with an upward trend, while the United States saw an increase from 2.2% before 2006 to 27% between 2006 and 2007 (16, 37, 38). Within China, alarming rates have been documented, such as 92% in Beijing and an average of 97.3% in Shanghai over the past three years, highlighting the severity of the issue. Prior to this research, information on *M. pneumoniae* resistance specifically in the Xi'an area was limited.

The molecular basis of the observed macrolide resistance is a key focus of this study. All 70 *M. pneumoniae* strains analyzed harbored mutations in the 23S rRNA gene, specifically A2063G, A2064T, and A2617C. It is well-established that the excessive or inappropriate use of antibiotics can drive the selection and proliferation of resistant bacterial strains, often leading to more severe clinical symptoms and complicated treatment pathways. Mutations at positions A2063 and A2064 in domain V of the 23S rRNA are particularly significant, as they are known to confer high-level resistance to 14- and 15-membered ring macrolides (like erythromycin) and cross-resistance to other macrolides (39). These mutations reduce the binding affinity of macrolide antibiotics to the ribosome, thereby inhibiting protein synthesis. The prevalence and types of mutations can vary, impacting the degree of resistance. For example, Xin et al. (6) found that among 46 drug-resistant strains in Beijing, 40 had the A2063G mutation, 5 had A2064G, and 1 had A2063C. Cao et al. (8) reported 41 strains with A2063G, 4 with A2064G, and 1 with A2063T, with A2063G and A2064G mutants typically showing high-level resistance (MIC ≥32 mg/L) (40–42). The A2063G mutation is by far the most frequently observed resistance genotype worldwide, often resulting in very high MICs (e.g., erythromycin MIC 128–256 mg/L) (43). In contrast, the A2064G mutation, while still causing resistance, generally leads to a lower level of resistance (41). Other rarer mutations, such as A2063T/C or changes at position 2617 (e.g., A2617C, which was found in all our isolates), tend to confer even lower-level or partial resistance (44). For instance, mutations at position 2617 have been associated with erythromycin MICs in the range of ∼0.03–8 mg/L, which may fall near or below clinical resistance breakpoints (41, 42).

A striking observation in our study is the discordance between genotypic and phenotypic resistance: while 100% of the 70 strains possessed 23S rRNA mutations (A2063G, A2064T, and A2617C), the overall phenotypic macrolide resistance rate was 38.57% (27 resistant, 11 intermediate, 32 sensitive). This discrepancy can be explored through several lenses. The presence of “weaker” mutations, such as A2617C (found in all our strains), might lead to lower-level resistance that phenotypically manifests as susceptible or intermediate. Indeed, literature confirms that different 23S rRNA mutations lead to varying degrees of macrolide affinity reduction and MIC increase (42). Thus, an isolate carrying a less potent mutation might harbor a genotypic hallmark of resistance yet exhibit an MIC that classifies it as phenotypically susceptible. Conversely, though less common, some studies have reported isolates with elevated macrolide MICs despite lacking common 23S rRNA mutations, suggesting alternative resistance mechanisms such as ribosomal protein changes or efflux pumps (41, 42). While all our strains had mutations, the *degree* of phenotypic resistance could still be modulated by such factors or by complex interactions between the identified mutations (A2063G, A2064T, and A2617C). The universal detection of these three specific mutations in all isolates is unusual and may point to a unique regional strain characteristic or methodological factors influencing detection sensitivity. This complex interplay underscores that the mere presence of a 23S rRNA mutation is not an absolute determinant of resistance; rather, each mutation has a characteristic effect size, and unrecognized mechanisms can sometimes modulate the resistance phenotype.

In summary, the A2063G and A2064G mutations remain the primary drivers of macrolide resistance in *M. pneumoniae* with A2063G especially conferring high-level resistance. A2617C (and analogous 2617 mutations), while reported, tend to produce a weaker resistance phenotype, which can lead to genotype–phenotype mismatches in susceptibility testing (43). There have indeed been cases of discordance—both mutation-positive susceptible strains and mutation-negative resistant strains—reported in the literature. Multiple studies corroborate that such discordances arise from the variable impact of different 23S mutations and possibly additional factors (like ribosomal protein changes or efflux pumps) modulating resistance. These findings strengthen our discussion by highlighting that the presence of a 23S rRNA mutation is not an all-or-nothing determinant of resistance. Rather, each mutation has a characteristic effect size on macrolide susceptibility, and unrecognized mechanisms can sometimes substitute for, or compensate for, the classic mutations (41). A holistic view from the literature confirms that while genotypic assays are extremely useful, phenotypic testing and awareness of rare mutations or mechanisms remain critical to fully understand macrolide resistance in *M. pneumoniae* (42).

Methodologically, the MALDI-TOF MS-based SNP genotyping method employed in this study offers practical advantages, particularly in settings with limited resources for Whole Genome Sequencing (WGS), providing robust data on genotype distribution and key resistance mutations. However, WGS would offer more comprehensive genomic data. The finding that all isolates simultaneously carried mutations A2063G, A2064T, and A2617C is notable and warrants further investigation, potentially using WGS, to confirm these observations and explore if this represents a unique regional strain or if methodological factors played a role.

The significant resistance to multiple antibiotic classes observed in Xi'an aligns with broader trends reported across Asia and highlights the complexities in antimicrobial resistance mechanisms. The discordance between genotypic mutations and phenotypic resistance may be attributed to factors such as differential expression of mutations, the presence of compensatory genetic elements, or heterogeneous microbial populations within isolates. This significant genotype-phenotype gap highlights the limitations of relying solely on molecular assays for clinical decision-making and strongly reinforces the need for concurrent phenotypic susceptibility testing to accurately guide therapeutic choices. These findings have direct clinical implications, suggesting a need for routine surveillance of *M. pneumoniae* genotypes and their antimicrobial susceptibility patterns. Furthermore, treatment guidelines may require revision based on local resistance data. From a clinical perspective, distinguishing between macrolide-sensitive and macrolide-resistant *M. pneumoniae* (MRMP) infections before treatment is challenging, as initial symptoms are often identical. However, a key indicator for suspected MRMP is a lack of clinical improvement after 48–72 h of appropriate macrolide therapy. Persistent fever, unremitting cough, and worsening radiological findings despite treatment should prompt clinicians to consider macrolide resistance and switch to alternative agents. Consideration should be given to alternative therapeutic agents, such as fluoroquinolones and tetracyclines, as second-line options for *M. pneumoniae* infections in older children and adolescents, particularly when macrolide resistance is suspected or confirmed. However, the notable resistance to quinolones (31.43%) and tetracyclines (38.57%) found in this study also calls for cautious and informed use of these alternatives.

This study is not without limitations. The absence of detailed clinical characteristics of the patients prevents a deeper correlation between microbiological findings and clinical outcomes. Additionally, potential inter-patient strain differences were not extensively explored, and the limited number of strains collected in 2023 might temper the statistical robustness and generalizability of our findings. Moreover, the reported sequences have not yet been deposited in a public DNA sequence database, which is recommended for future studies to enhance data sharing and comparability.

In conclusion, the macrolide resistance rate of *M. pneumoniae* in Xi'an remains alarmingly elevated, characterized by the pervasive A2063G mutation in the V domain of 23S rRNA (alongside A2064T and A2617C) and a predominance of P1 I strains. These findings underscore the urgent need for continuous surveillance and the revision of current treatment guidelines. It is also important to note that resistance in *M. pneumoniae* is primarily driven by chromosomal point mutations rather than plasmid-mediated horizontal gene transfer. While this limits inter-species spread, the fundamental principle of preventing resistance development remains paramount. This reinforces the urgent need for robust antimicrobial stewardship programs—including diagnostics-guided therapy and avoiding unnecessary antibiotic use—to preserve the efficacy of remaining second-line agents. While alternative antimicrobial agents such as tetracyclines and fluoroquinolones are considered second-line options, the high resistance rates observed in this study (38.6% and 31.4%, respectively) demand extreme caution. Therefore, their use should be guided by specific susceptibility testing rather than empirical prescription to ensure efficacy and prevent the further spread of multidrug-resistant strains. Future research, ideally incorporating WGS and comprehensive clinical data, is crucial for a more profound understanding of the evolving epidemiology and resistance mechanisms of *M. pneumoniae*.

## Data Availability

The original contributions presented in the study are included in the article/Supplementary Material, further inquiries can be directed to the corresponding authors.
