# Supplementary material for: Antimicrobial susceptibility and molecular characteristics of Mycoplasma pneumoniae isolates from children in Xi'an regions of China
Source: Front Pediatr. 2025 Oct 15;13:1660687. doi: 10.3389/fped.2025.1660687 (PMC12568347; doi:10.3389/fped.2025.1660687)
Supplement: Supplementary file 1 [file Datasheet1.pdf]

Table S1. Primer sequences for *M. pneumoniae* SNP typing and susceptibility target site amplification and MPE probes for SNP detection														
			Multiple-PCR		Mass probe extension									
	Target gene	SNP	Forward primer sequence	Reverse primer sequence	Mass probe sequence	Mass probe Mass (Da)	Extension call	Extended mass (Da)	Extension call	Extended mass (Da)	Extension call	Extended mass (Da)	Extension call	Extended mass (Da)
GENO TYPING	MP N114^{1461}	C/T	acgttggatgCA CCGAGTGTC TTCGTCCTT	acgttggatgGGT TGACCCCAC TACCCTTT	CCCTTTTGC GCTAACCG	5097.4	C	5410.4	T	5394.4				
	MP N126^{470}	C/T	acgttggatgAA ATTCCCCGT AATCCGAGA	acgttggatgGCC TTCTGCTTG TTAGGTAGA TG	AGTGATTT AACGTTCC ATTTTC	6690.4	C	6963.4	T	7032.4				
	MP N213^{47}	G/T	acgttggatgATC AGTCGCTGG TACTGTGG	acgttggatgGC TGTGCTTTT GGCAACTTA	TACTACCAC CGCAGTTGT T	5738.8	G	6011.8	T	6035.8				
	MP N262^{192}	C/T	acgttggatgACC ACTTAGAGC CGGAACAA	acgttggatgTT GCTGAAACT GACCATCCA	GCCCAATTT AGTCTTATTT TGGAT	7323.8	C	7636.8	T	7620.8				
	MP N280^{1641}	G/T	acgttggatgCTC AATTAAACG CGGTTTGG	acgttggatgCGG TTGAAATCA ACGGGTTA	TTCCAAAT GAAGTAAGA CAC	6118.0	G	6391.0	T	6415.0				
	MP N372^{1112}	G/T	cgttggatgAGT GTTAGCGCG GTTAATGC	acgttggatgTC GTGTTGTGA ACCACCACT	AATGACAC CGCAAGACA	5181.4	T	5523.4	G	5494.4				
ML DETECTION	23S rRNA^{2063}	A/T/ C/G	acgttggatgCCA GGTACGGGT GAAGACAC	acgttggatgATT CCACCTTTC GCATCAAC	TTAGGCGCA ACGGGACGG	5589.6	G	5902.6	A	5886.6	C	5862.6	T	5931.6
	23S rRNA^{2064}	A/T/ C/G	acgttggatgCCA GGTACGGGT GAAGACAC	acgttggatgATT CCACCTTTC GCATCAAC	AGCTACAGT AAAGCTTCA CGGGGTCT	7995.2	T	8337.2	C	8268.2	G	8308.2	A	8292.2
	23S rRNA^{2617}	A/T/ C/G	acgttggatgGCT GTTCGCCGA TTAAAGAG	acgttggatgGCG CTACAACTG GAGCATAA	CCGTCGT GAGACAGG TTGGTC	6478.2	C	6751.2	G	6791.2	A	6775.2	T	6820.2

Table S2. *M .pneumoniae* drug sensitivity results														
Sample	Erythromycin Estolate	Erythromycin	Azithromycin	Josamycin	Acetylspiramycin	Clindamycin	Clarithromycin	Roxithromycin	Ciprofloxacin	Moxifloxacin	Levofloxacin	Gatifloxacin	Minocycline	Doxycycline
1	Sensitive	Sensitive	Sensitive	Sensitive	Sensitive	Sensitive	Sensitive	Sensitive	Intermediary	Sensitive	Intermediary	Sensitive	Sensitive	Intermediary
2	Sensitive	Sensitive	Sensitive	Sensitive	Sensitive	Sensitive	Sensitive	Sensitive	Intermediary	Intermediary	Intermediary	Intermediary	Sensitive	Resistance
3	Sensitive	Sensitive	Intermediary	Resistance	Resistance	Intermediary	Sensitive	Intermediary	Sensitive	Intermediary	Sensitive	Sensitive	Sensitive	Intermediary
4	Sensitive	Sensitive	Sensitive	Sensitive	Sensitive	Sensitive	Sensitive	Sensitive	Intermediary	Sensitive	Intermediary	Intermediary	Sensitive	Resistance
5	Sensitive	Sensitive	Sensitive	Sensitive	Sensitive	Sensitive	Sensitive	Sensitive	Resistance	Intermediary	Intermediary	Intermediary	Resistance	Resistance
6	Resistance	Intermediary	Intermediary	Resistance	Resistance	Intermediary	Sensitive	Sensitive	Sensitive	Intermediary	Sensitive	Intermediary	Intermediary	Resistance
7	Sensitive	Sensitive	Sensitive	Sensitive	Intermediary	Intermediary	Sensitive	Sensitive	Resistance	Resistance	Resistance	Resistance	Resistance	Resistance
8	Sensitive	Sensitive	Sensitive	Sensitive	Sensitive	Sensitive	Sensitive	Sensitive	Resistance	Resistance	Intermediary	Intermediary	Intermediary	Sensitive
9	Sensitive	Sensitive	Sensitive	Sensitive	Intermediary	Sensitive	Sensitive	Sensitive	Sensitive	Sensitive	Sensitive	Sensitive	Sensitive	Intermediary
10	Sensitive	Sensitive	Sensitive	Sensitive	Sensitive	Sensitive	Sensitive	Sensitive	Sensitive	Sensitive	Sensitive	Sensitive	Sensitive	Sensitive
11	Sensitive	Sensitive	Sensitive	Intermediary	Resistance	Sensitive	Sensitive	Sensitive	Sensitive	Sensitive	Sensitive	Sensitive	Intermediary	Intermediary
12	Sensitive	Sensitive	Sensitive	Sensitive	Sensitive	Sensitive	Sensitive	Sensitive	Resistance	Resistance	Resistance	Resistance	Sensitive	Intermediary
13	Sensitive	Sensitive	Sensitive	Sensitive	Sensitive	Sensitive	Sensitive	Sensitive	Sensitive	Sensitive	Sensitive	Sensitive	Sensitive	Intermediary
14	Sensitive	Sensitive	Sensitive	Intermediary	Intermediary	Intermediary	Sensitive	Sensitive	Sensitive	Intermediary	Sensitive	Sensitive	Intermediary	Resistance
15	Sensitive	Sensitive	Sensitive	Sensitive	Sensitive	Sensitive	Sensitive	Sensitive	Intermediary	Sensitive	Intermediary	Intermediary	Sensitive	Resistance
16	Sensitive	Sensitive	Sensitive	Intermediary	Resistance	Resistance	Sensitive	Sensitive	Sensitive	Intermediary	Sensitive	Sensitive	Intermediary	Intermediary
17	Sensitive	Sensitive	Sensitive	Sensitive	Sensitive	Sensitive	Sensitive	Sensitive	Sensitive	Sensitive	Sensitive	Sensitive	Sensitive	Sensitive
18	Sensitive	Sensitive	Sensitive	Sensitive	Sensitive	Sensitive	Sensitive	Sensitive	Resistance	Resistance	Resistance	Resistance	Sensitive	Resistance
19	Sensitive	Sensitive	Sensitive	Sensitive	Sensitive	Sensitive	Sensitive	Sensitive	Resistance	Resistance	Resistance	Resistance	Sensitive	Intermediary
20	Sensitive	Sensitive	Sensitive	Sensitive	Sensitive	Sensitive	Sensitive	Sensitive	Resistance	Resistance	Resistance	Resistance	Sensitive	Intermediary
21	Sensitive	Sensitive	Sensitive	Sensitive	Sensitive	Sensitive	Sensitive	Sensitive	Intermediary	Intermediary	Intermediary	Intermediary	Sensitive	Intermediary
22	Resistance	Sensitive	Sensitive	Intermediary	Resistance	Resistance	Sensitive	Sensitive	Sensitive	Resistance	Sensitive	Sensitive	Intermediary	Intermediary
23	Resistance	Sensitive	Sensitive	Intermediary	Resistance	Sensitive	Sensitive	Sensitive	Sensitive	Sensitive	Sensitive	Sensitive	Intermediary	Resistance
24	Sensitive	Sensitive	Sensitive	Sensitive	Intermediary	Intermediary	Sensitive	Sensitive	Intermediary	Resistance	Sensitive	Sensitive	Intermediary	Intermediary
25	Sensitive	Sensitive	Sensitive	Sensitive	Sensitive	Sensitive	Sensitive	Sensitive	Intermediary	Sensitive	Intermediary	Intermediary	Sensitive	Resistance
26	Sensitive	Sensitive	Sensitive	Sensitive	Intermediary	Intermediary	Sensitive	Sensitive	Intermediary	Resistance	Intermediary	Intermediary	Sensitive	Intermediary
27	Sensitive	Sensitive	Sensitive	Intermediary	Intermediary	Intermediary	Sensitive	Sensitive	Intermediary	Resistance	Intermediary	Intermediary	Intermediary	Intermediary
28	Sensitive	Sensitive	Sensitive	Sensitive	Sensitive	Sensitive	Sensitive	Sensitive	Intermediary	Intermediary	Intermediary	Intermediary	Intermediary	Resistance
29	Resistance	Sensitive	Sensitive	Resistance	Resistance	Sensitive	Sensitive	Sensitive	Sensitive	Intermediary	Sensitive	Sensitive	Intermediary	Resistance
30	Sensitive	Sensitive	Sensitive	Sensitive	Sensitive	Sensitive	Sensitive	Sensitive	Intermediary	Sensitive	Sensitive	Sensitive	Intermediary	Resistance
31	Sensitive	Sensitive	Sensitive	Sensitive	Sensitive	Sensitive	Sensitive	Sensitive	Resistance	Resistance	Resistance	Resistance	Sensitive	Intermediary
32	Sensitive	Sensitive	Sensitive	Sensitive	Sensitive	Sensitive	Sensitive	Sensitive	Resistance	Resistance	Resistance	Resistance	Sensitive	Resistance
33	Sensitive	Sensitive	Intermediary	Resistance	Resistance	Intermediary	Sensitive	Sensitive	Resistance	Resistance	Resistance	Resistance	Intermediary	Resistance
34	Sensitive	Sensitive	Sensitive	Sensitive	Sensitive	Sensitive	Sensitive	Intermediary	Intermediary	Sensitive	Sensitive	Sensitive	Sensitive	Intermediary
35	Sensitive	Sensitive	Sensitive	Sensitive	Sensitive	Sensitive	Sensitive	Sensitive	Resistance	Resistance	Resistance	Resistance	Sensitive	Resistance
36	Resistance	Sensitive	Sensitive	Intermediary	Resistance	Intermediary	Sensitive	Sensitive	Sensitive	Intermediary	Sensitive	Sensitive	Intermediary	Intermediary
37	Sensitive	Sensitive	Sensitive	Intermediary	Resistance	Intermediary	Sensitive	Sensitive	Resistance	Resistance	Intermediary	Intermediary	Intermediary	Intermediary
38	Sensitive	Sensitive	Sensitive	Intermediary	Resistance	Sensitive	Sensitive	Sensitive	Sensitive	Intermediary	Sensitive	Sensitive	Intermediary	Intermediary
39	Sensitive	Sensitive	Sensitive	Intermediary	Intermediary	Sensitive	Sensitive	Sensitive	Sensitive	Sensitive	Sensitive	Sensitive	Sensitive	Sensitive
40	Sensitive	Sensitive	Sensitive	Sensitive	Sensitive	Intermediary	Sensitive	Sensitive	Sensitive	Sensitive	Sensitive	Sensitive	Sensitive	Sensitive
41	Sensitive	Sensitive	Sensitive	Sensitive	Sensitive	Sensitive	Sensitive	Sensitive	Resistance	Resistance	Resistance	Resistance	Sensitive	Intermediary
42	Resistance	Resistance	Resistance	Sensitive	Sensitive	Resistance	Resistance	Resistance	Intermediary	Sensitive	Sensitive	Sensitive	Intermediary	Intermediary
43	Sensitive	Sensitive	Sensitive	Intermediary	Resistance	Sensitive	Sensitive	Sensitive	Sensitive	Sensitive	Sensitive	Sensitive	Intermediary	Intermediary

44	Sensitive	Sensitive	Sensitive	Sensitive	Sensitive	Sensitive	Sensitive	Sensitive	Sensitive	Sensitive	Sensitive	Sensitive	Sensitive	Intermediary
45	Sensitive	Sensitive	Sensitive	Sensitive	Sensitive	Sensitive	Sensitive	Sensitive	Resistance	Resistance	Resistance	Intermediary	Sensitive	Intermediary
46	Sensitive	Sensitive	Sensitive	Sensitive	Sensitive	Sensitive	Intermediary	Sensitive	Sensitive	Sensitive	Sensitive	Sensitive	Sensitive	Sensitive
47	Resistance	Intermediary	Sensitive	Sensitive	Sensitive	Resistance	Resistance	Intermediary	Sensitive	Sensitive	Sensitive	Sensitive	Sensitive	Sensitive
48	Resistance	Resistance	Intermediary	Intermediary	Sensitive	Resistance	Resistance	Resistance	Sensitive	Sensitive	Sensitive	Sensitive	Sensitive	Intermediary
49	Sensitive	Sensitive	Sensitive	Sensitive	Sensitive	Sensitive	Sensitive	Sensitive	Sensitive	Sensitive	Sensitive	Sensitive	Intermediary	Intermediary
50	Resistance	Resistance	Resistance	Intermediary	Sensitive	Resistance	Resistance	Resistance	Sensitive	Sensitive	Sensitive	Sensitive	Intermediary	Intermediary
51	Sensitive	Sensitive	Sensitive	Sensitive	Sensitive	Sensitive	Sensitive	Sensitive	Sensitive	Sensitive	Sensitive	Sensitive	Sensitive	Intermediary
52	Sensitive	Sensitive	Sensitive	Sensitive	Sensitive	Sensitive	Sensitive	Sensitive	Intermediary	Sensitive	Sensitive	Sensitive	Sensitive	Resistance
53	Sensitive	Intermediary	Intermediary	Sensitive	Sensitive	Intermediary	Intermediary	Intermediary	Sensitive	Sensitive	Sensitive	Sensitive	Sensitive	Sensitive
54	Sensitive	Sensitive	Sensitive	Sensitive	Sensitive	Sensitive	Sensitive	Sensitive	Resistance	Resistance	Resistance	Resistance	Resistance	Sensitive
55	Sensitive	Sensitive	Sensitive	Sensitive	Sensitive	Sensitive	Sensitive	Sensitive	Resistance	Resistance	Resistance	Resistance	Resistance	Sensitive
56	Intermediary	Sensitive	Sensitive	Sensitive	Sensitive	Sensitive	Sensitive	Sensitive	Resistance	Resistance	Resistance	Resistance	Resistance	Sensitive
57	Sensitive	Sensitive	Sensitive	Sensitive	Sensitive	Sensitive	Sensitive	Sensitive	Intermediary	Sensitive	Sensitive	Sensitive	Intermediary	Sensitive
58	Sensitive	Sensitive	Sensitive	Sensitive	Sensitive	Sensitive	Sensitive	Sensitive	Intermediary	Sensitive	Intermediary	Intermediary	Intermediary	Sensitive
59	Sensitive	Intermediary	Intermediary	Sensitive	Sensitive	Intermediary	Intermediary	Sensitive	Sensitive	Sensitive	Sensitive	Sensitive	Sensitive	Sensitive
60	Resistance	Resistance	Resistance	Intermediary	Sensitive	Resistance	Resistance	Resistance	Sensitive	Sensitive	Sensitive	Sensitive	Sensitive	Sensitive
61	Resistance	Resistance	Resistance	Resistance	Sensitive	Resistance	Resistance	Resistance	Sensitive	Sensitive	Sensitive	Sensitive	Sensitive	Sensitive
62	Resistance	Resistance	Resistance	Resistance	Sensitive	Resistance	Resistance	Resistance	Intermediary	Sensitive	Sensitive	Sensitive	Sensitive	Resistance
63	Sensitive	Intermediary	Sensitive	Resistance	Resistance	Intermediary	Intermediary	Intermediary	Sensitive	Sensitive	Intermediary	Sensitive	Resistance	Resistance
64	Resistance	Resistance	Resistance	Resistance	Intermediary	Resistance	Resistance	Resistance	Sensitive	Sensitive	Sensitive	Sensitive	Sensitive	Sensitive
65	Resistance	Resistance	Resistance	Resistance	Intermediary	Resistance	Resistance	Resistance	Sensitive	Sensitive	Sensitive	Sensitive	Intermediary	Intermediary
66	Sensitive	Sensitive	Sensitive	Sensitive	Sensitive	Sensitive	Sensitive	Sensitive	Intermediary	Sensitive	Intermediary	Intermediary	Sensitive	Resistance
67	Sensitive	Sensitive	Sensitive	Sensitive	Sensitive	Resistance	Sensitive	Sensitive	Intermediary	Sensitive	Sensitive	Sensitive	Sensitive	Resistance
68	Sensitive	Resistance	Resistance	Sensitive	Sensitive	Sensitive	Intermediary	Sensitive	Sensitive	Sensitive	Sensitive	Sensitive	Sensitive	Sensitive
69	Sensitive	Sensitive	Sensitive	Sensitive	Sensitive	Sensitive	Sensitive	Sensitive	Resistance	Resistance	Resistance	Resistance	Sensitive	Intermediary
70	Sensitive	Intermediary	Resistance	Sensitive	Sensitive	Intermediary	Intermediary	Sensitive	Sensitive	Sensitive	Sensitive	Sensitive	Sensitive	Sensitive

Table S3. MIC (MIC, in mg/L) of different antimicrobials of *M .pneumoniae*

Antibiotics	Low concentration（mg/L）	High concentration（mg/L）
Erythromycin estolate	1	4
Erythromycin	2	8
Azithromycin	1	4
Clarithromycin	1	4
Roxithromycin	1	4
Josamycin	2	8
Acetylspiramycin	1	4
Ciprofloxacin	1	4
Moxifloxacin	1	4
Levofloxacin	1	4
Gatifloxacin	1	4
Minocycline	2	8
Doxycycline	4	8
Clindamycin	1	4

Sample-1

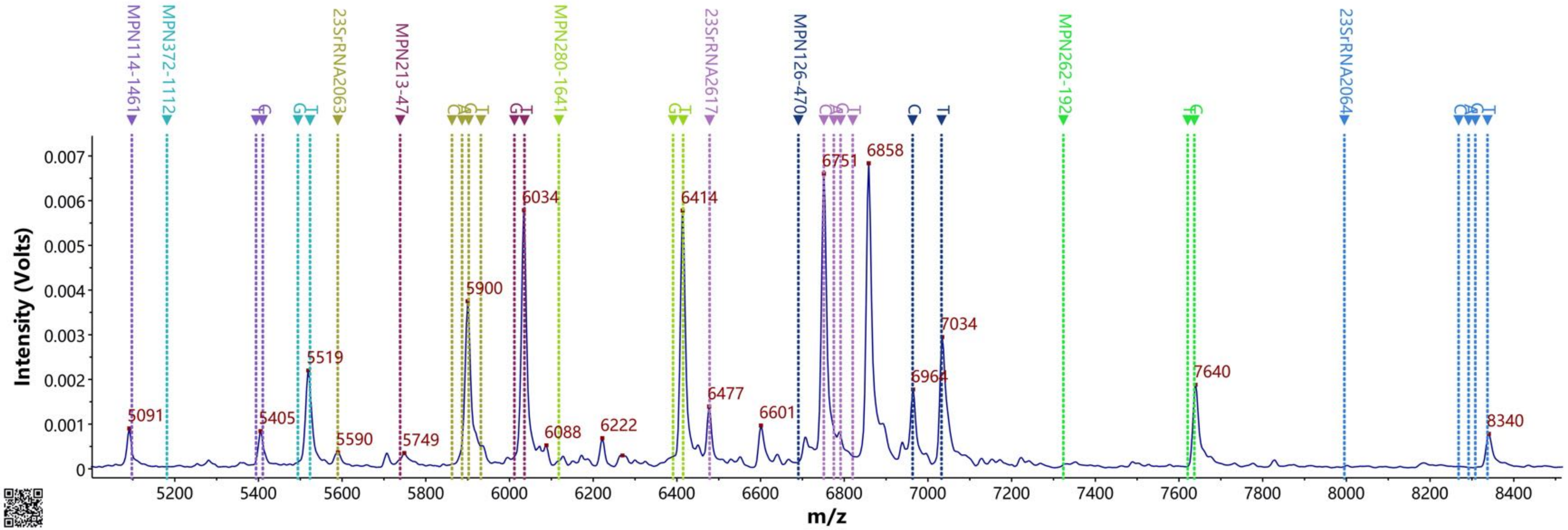
MPN114-1461
MPN372-1112
23SrRNA2063
MPN213-47
MPN280-1641
23SrRNA2617
MPN126-470
MPN262-192
23SrRNA2064
Intensity (Volts)
m/z
5091
5405
5519
5590
5749
5900
6034
6088
6222
6414
6477
6601
6751
6858
6964
7034
7640
8340

Sample-2

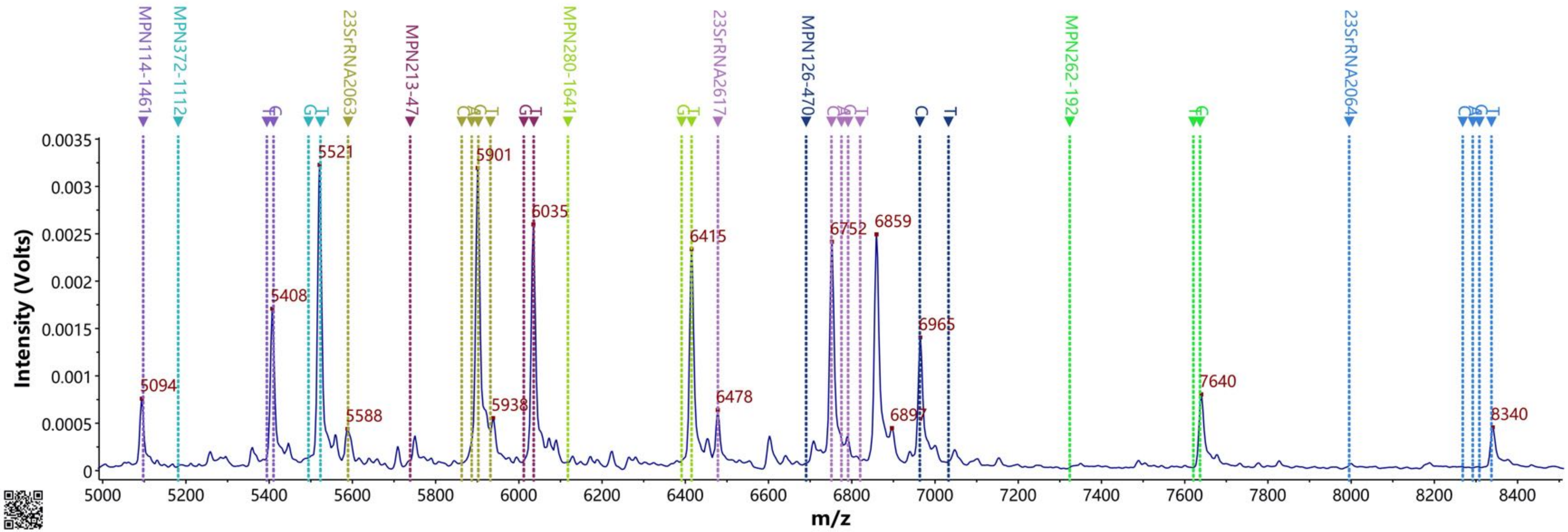

MPN114-1461
MPN372-1112
23SrRNA2063
MPN213-47
MPN280-1641
23SrRNA2617
MPN126-470
MPN262-192
23SrRNA2064
5094
5408
5521
5588
5901
5938
6035
6415
6478
6752
6859
6897
6965
7640
8340
Intensity (Volts)
m/z

Sample-3

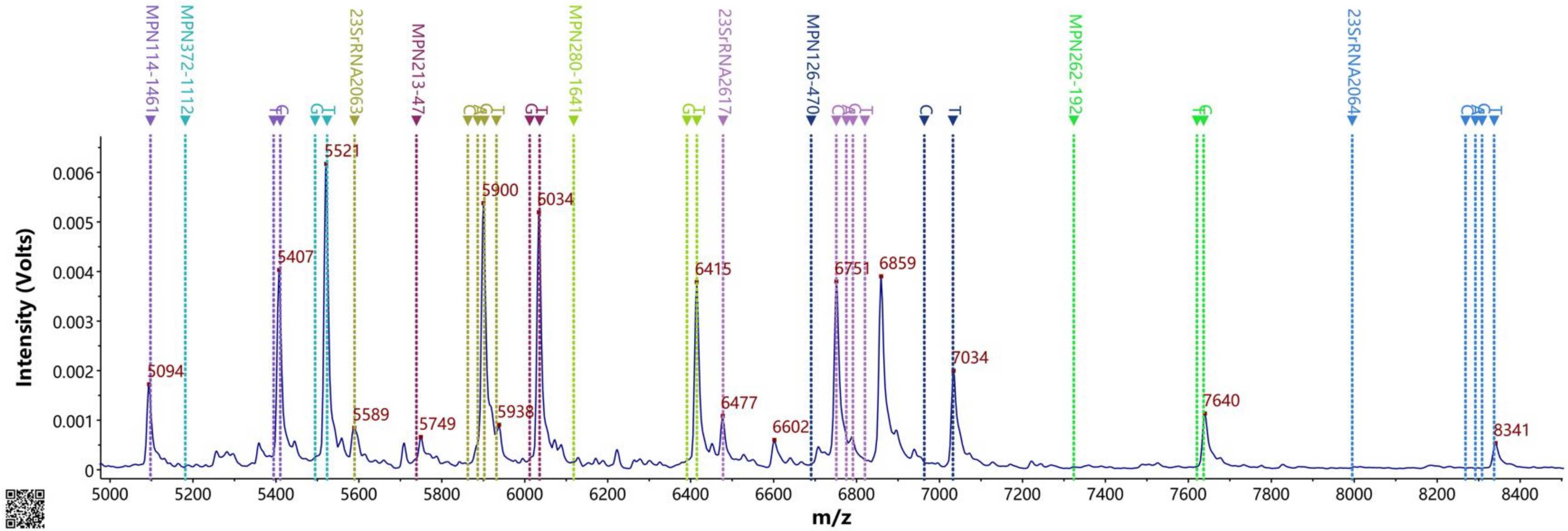

MPN114-1461
MPN372-1112
23SrRNA2063
MPN213-47
MPN280-1641
23SrRNA2617
MPN126-470
MPN262-192
23SrRNA2064
5094
5407
5521
5589
5749
5900
5938
6034
6415
6477
6602
6751
6859
7034
7640
8341
Intensity (Volts)
m/z

Sample-4

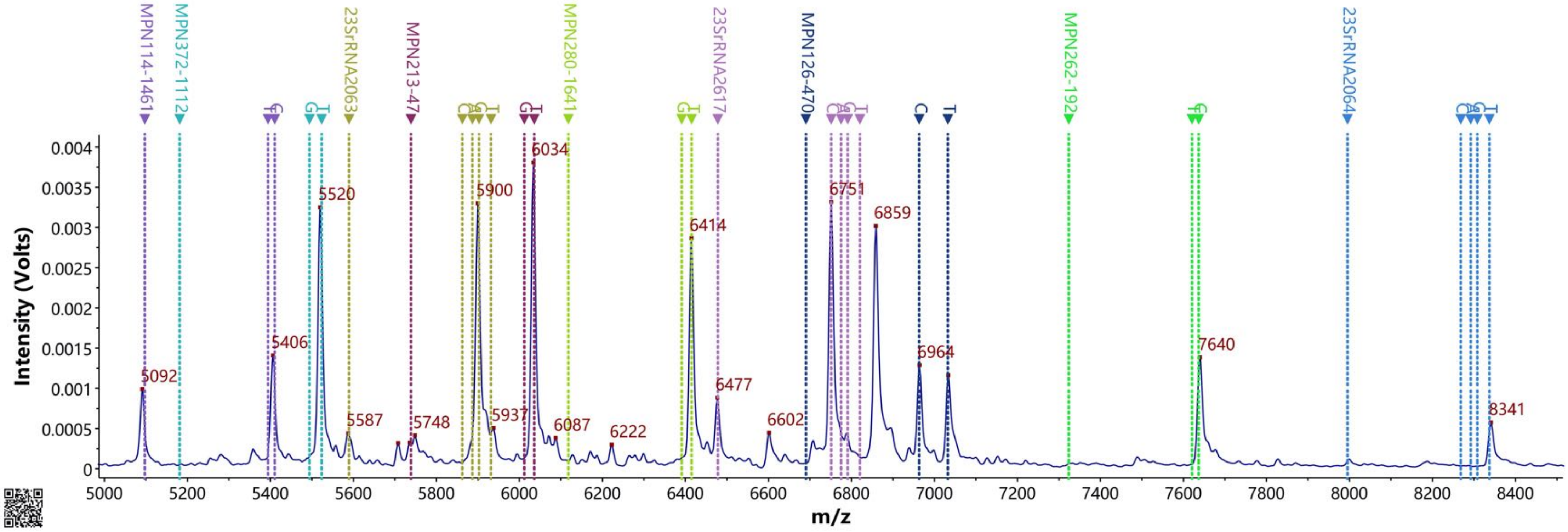

MPN114-1461
MPN372-1112
23SrRNA2063
MPN213-47
MPN280-1641
23SrRNA2617
MPN126-470
MPN262-192
23SrRNA2064
5092
5406
5520
5587
5748
5900
5937
6034
6087
6222
6414
6477
6602
6751
6859
6964
7640
8341
Intensity (Volts)
m/z

Sample-5

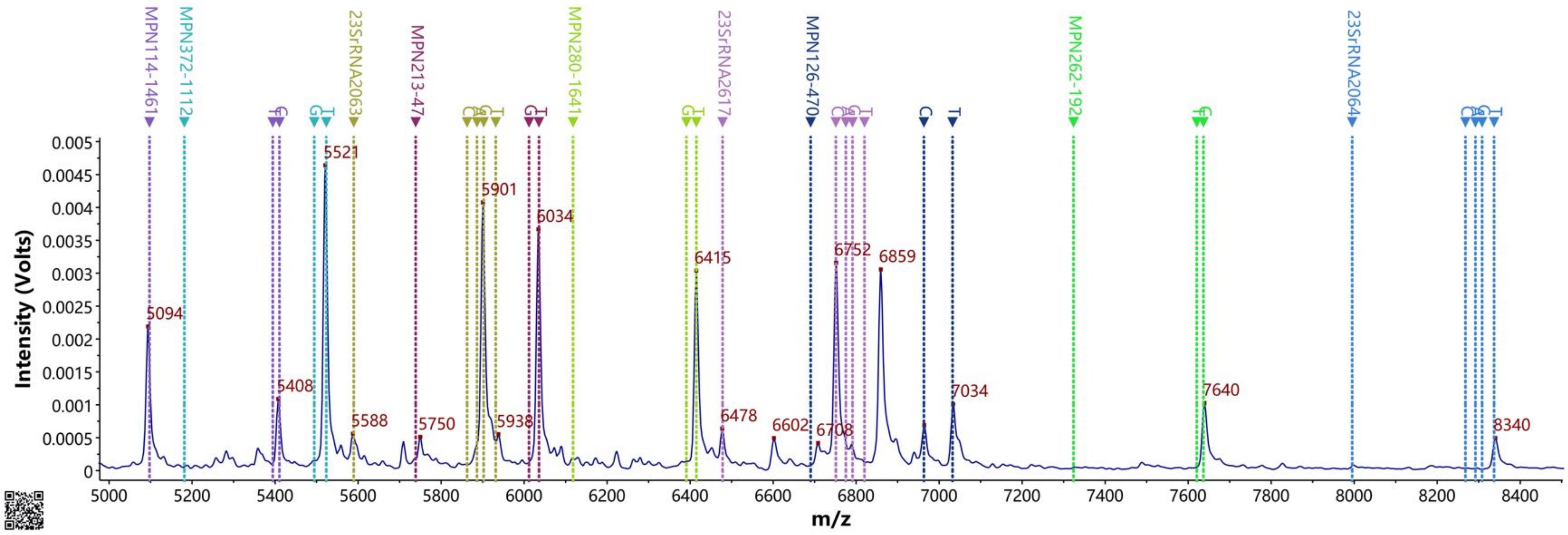

MPN114-1461
MPN372-1112
23SrRNA2063
MPN213-47
MPN280-1641
23SrRNA2617
MPN126-470
MPN262-192
23SrRNA2064
Intensity (Volts)
m/z
5094
5408
5521
5588
5750
5901
5938
6034
6415
6478
6602
6708
6752
6859
7034
7640
8340

Sample-6

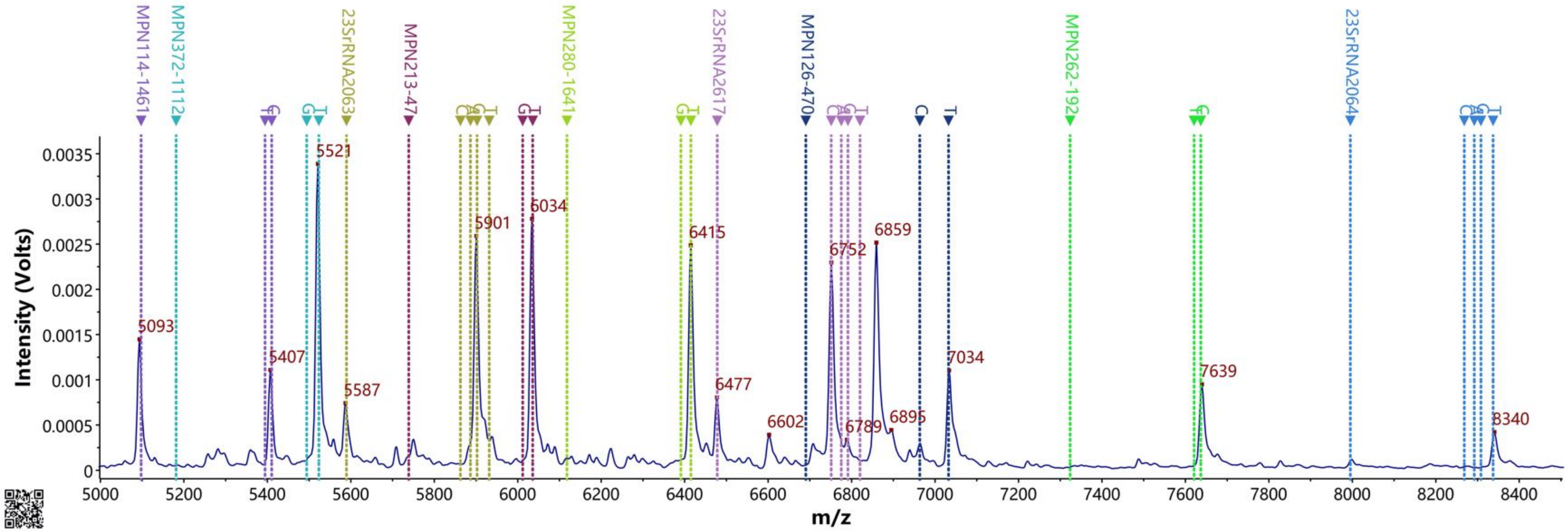

MPN114-1461
MPN372-1112
23SrRNA2063
MPN213-47
MPN280-1641
23SrRNA2617
MPN126-470
MPN262-192
23SrRNA2064
5093
5407
5521
5587
5901
6034
6415
6477
6602
6752
6789
6859
6895
7034
7639
8340
Intensity (Volts)
m/z

Sample-7

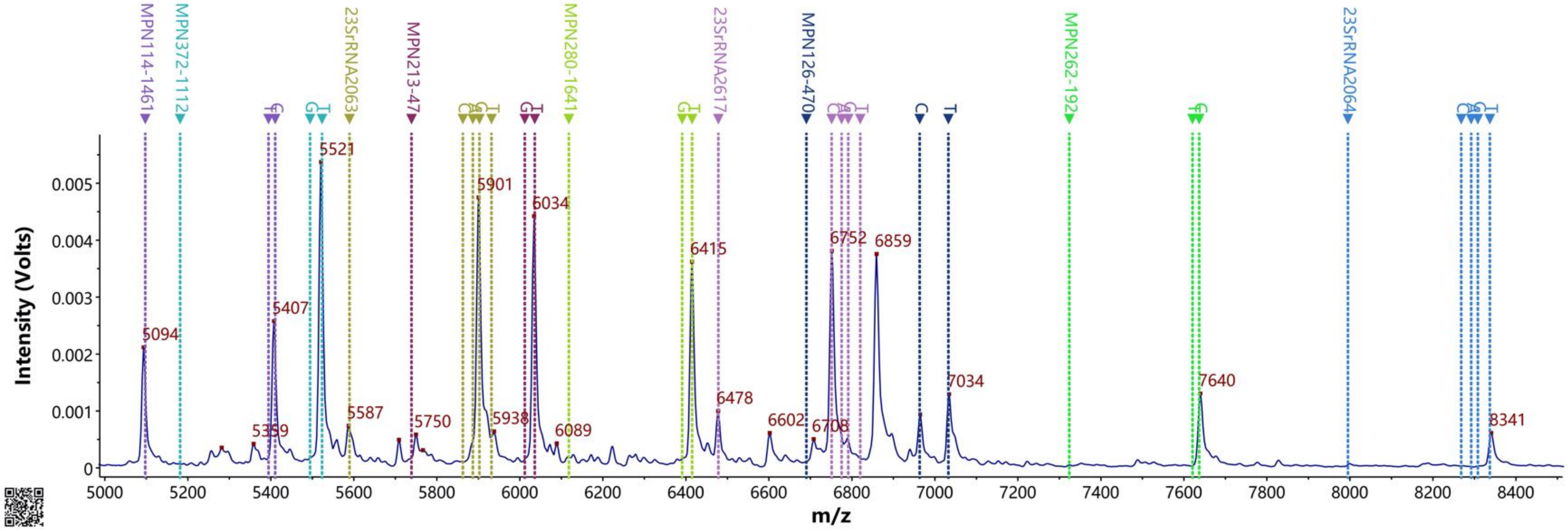
MPN114-1461
MPN372-1112
23SrRNA2063
MPN213-47
MPN280-1641
23SrRNA2617
MPN126-470
MPN262-192
23SrRNA2064
5094
5359
5407
5521
5587
5750
5901
5938
6034
6089
6415
6478
6602
6708
6752
6859
7034
7640
8341
Intensity (Volts)
m/z

Sample-8

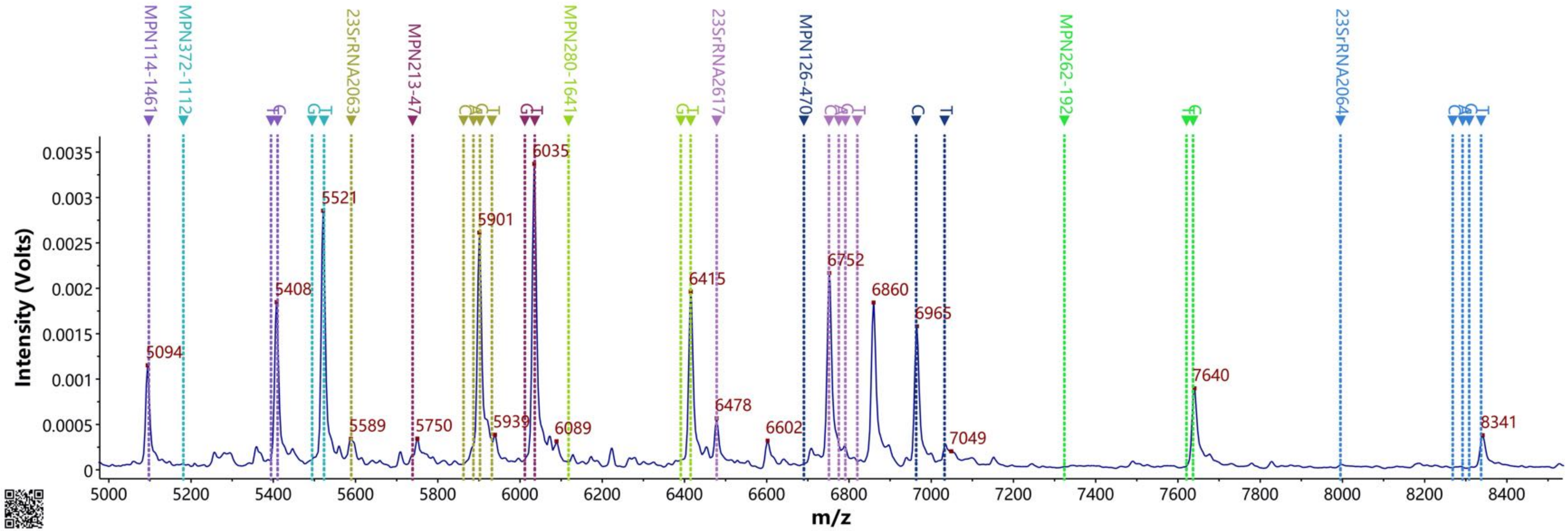

MPN114-1461
MPN372-1112
23SrRNA2063
MPN213-47
MPN280-1641
23SrRNA2617
MPN126-470
MPN262-192
23SrRNA2064
Intensity (Volts)
m/z
5094
5408
5521
5589
5750
5901
5939
6035
6089
6415
6478
6602
6752
6860
6965
7049
7640
8341

Sample-9

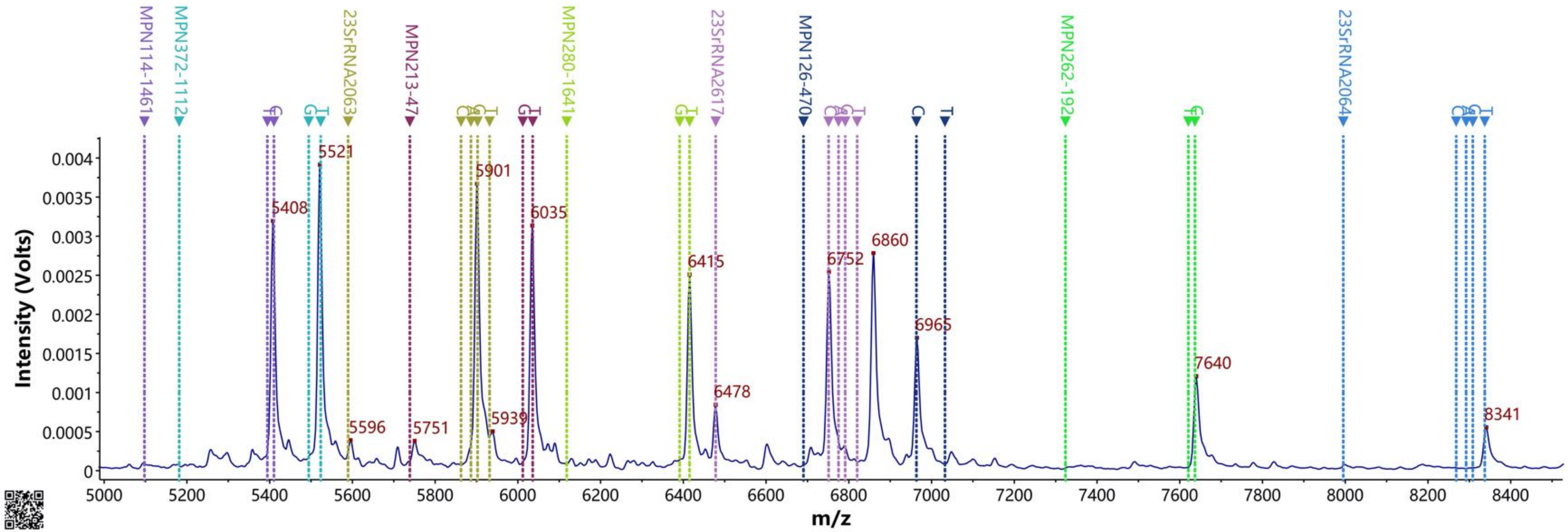

MPN114-1461
MPN372-1112
23SrRNA2063
MPN213-47
MPN280-1641
23SrRNA2617
MPN126-470
MPN262-192
23SrRNA2064
5408
5521
5596
5751
5901
5939
6035
6415
6478
6752
6860
6965
7640
8341
Intensity (Volts)
m/z

Sample-10

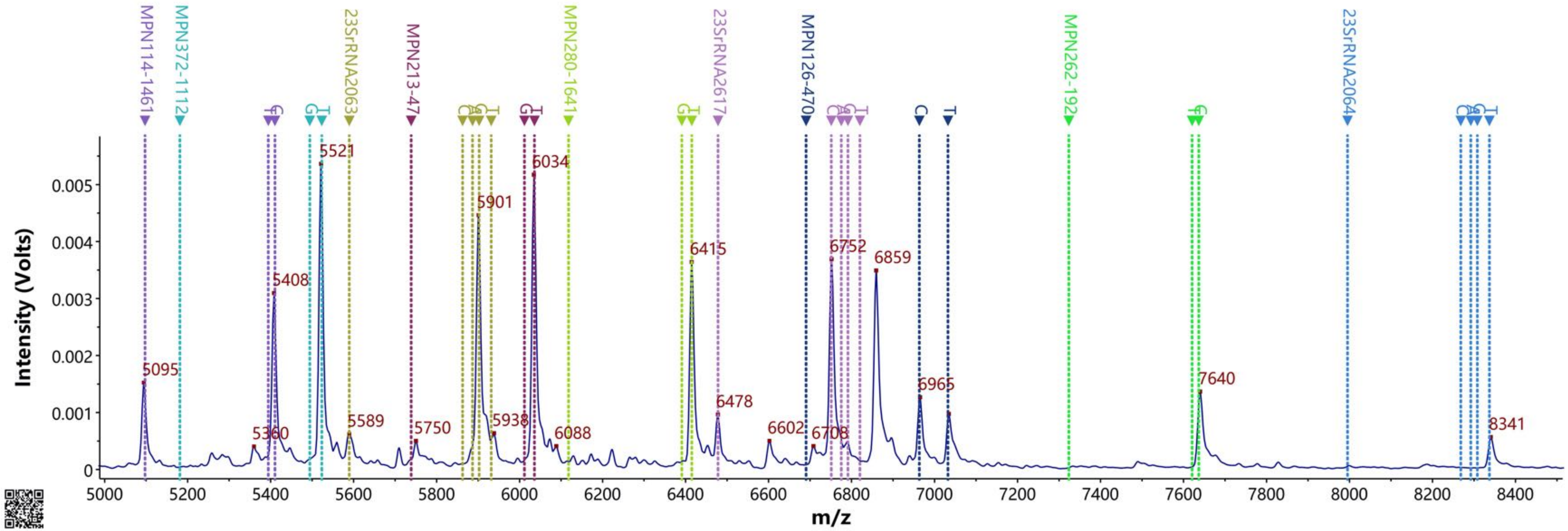

MPN114-1461
MPN372-1112
23SrRNA2063
MPN213-47
MPN280-1641
23SrRNA2617
MPN126-470
MPN262-192
23SrRNA2064
5095
5360
5408
5521
5589
5750
5901
5938
6034
6088
6415
6478
6602
6708
6752
6859
6965
7640
8341
Intensity (Volts)
m/z

Sample-11

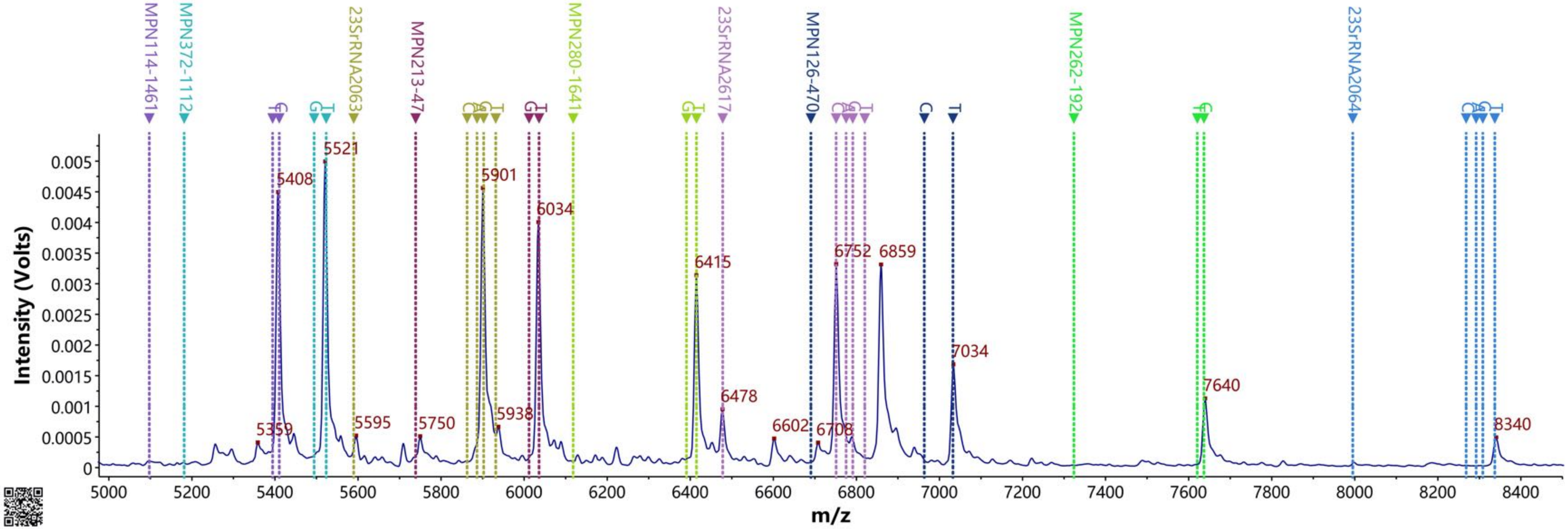

MPN114-1461
MPN372-1112
23SrRNA2063
MPN213-47
MPN280-1641
23SrRNA2617
MPN126-470
MPN262-192
23SrRNA2064
Intensity (Volts)
m/z
5359
5408
5521
5595
5750
5901
5938
6034
6415
6478
6602
6708
6752
6859
7034
7640
8340

Sample-12

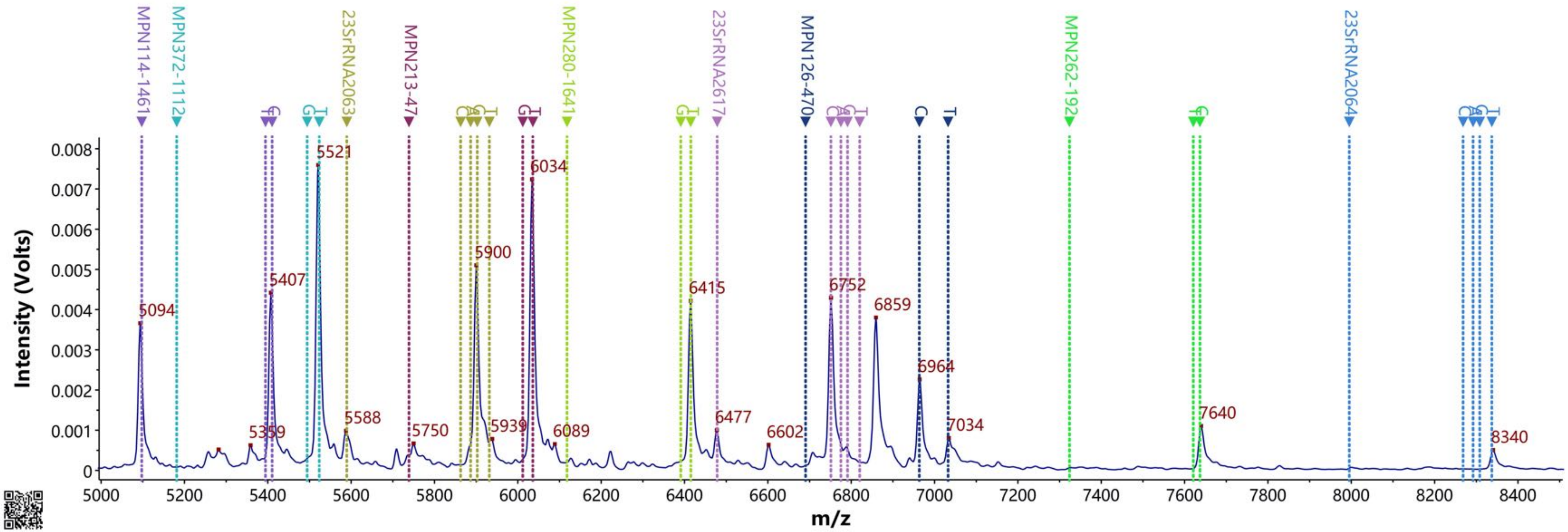

MPN114-1461
MPN372-1112
23SrRNA2063
MPN213-47
MPN280-1641
23SrRNA2617
MPN126-470
MPN262-192
23SrRNA2064
5094
5359
5407
5521
5588
5750
5900
5939
6034
6089
6415
6477
6602
6752
6859
6964
7034
7640
8340
Intensity (Volts)
m/z

Sample-13

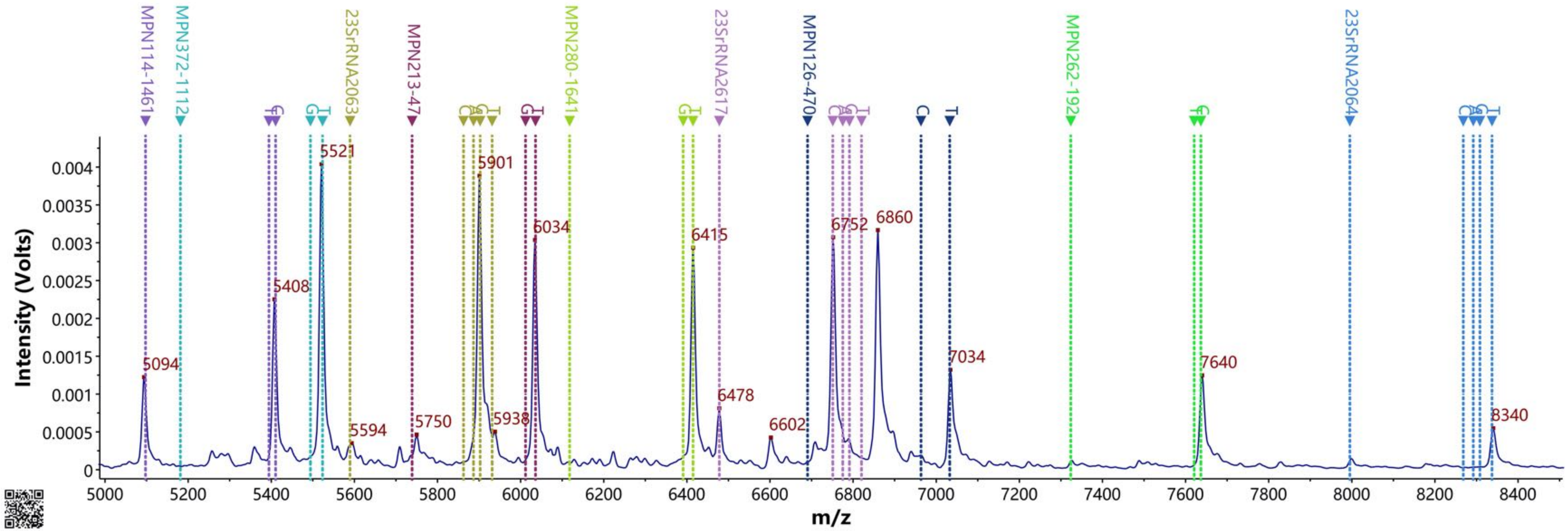

MPN114-1461
MPN372-1112
23SrRNA2063
MPN213-47
MPN280-1641
23SrRNA2617
MPN126-470
MPN262-192
23SrRNA2064
5094
5408
5521
5594
5750
5901
5938
6034
6415
6478
6602
6752
6860
7034
7640
8340
Intensity (Volts)
m/z

Sample-14

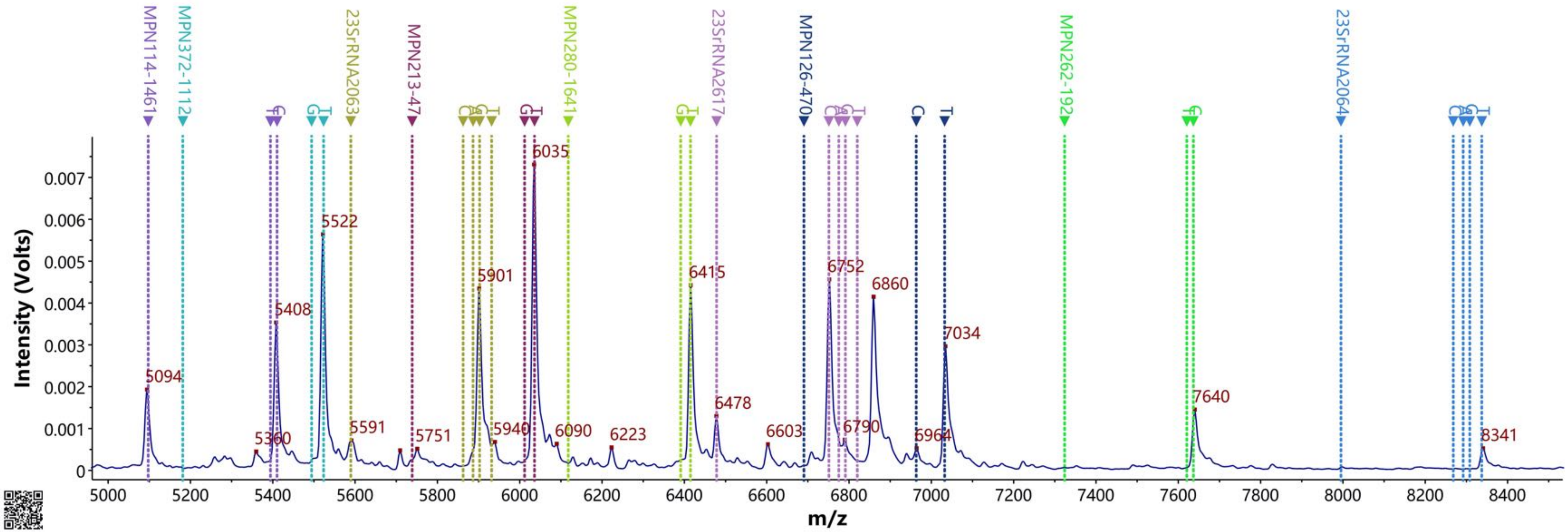
MPN114-1461
MPN372-1112
23SrRNA2063
MPN213-47
MPN280-1641
23SrRNA2617
MPN126-470
MPN262-192
23SrRNA2064
Intensity (Volts)
m/z
5094
5360
5408
5522
5591
5751
5901
5940
6035
6090
6223
6415
6478
6603
6752
6790
6860
6964
7034
7640
8341

Sample-15

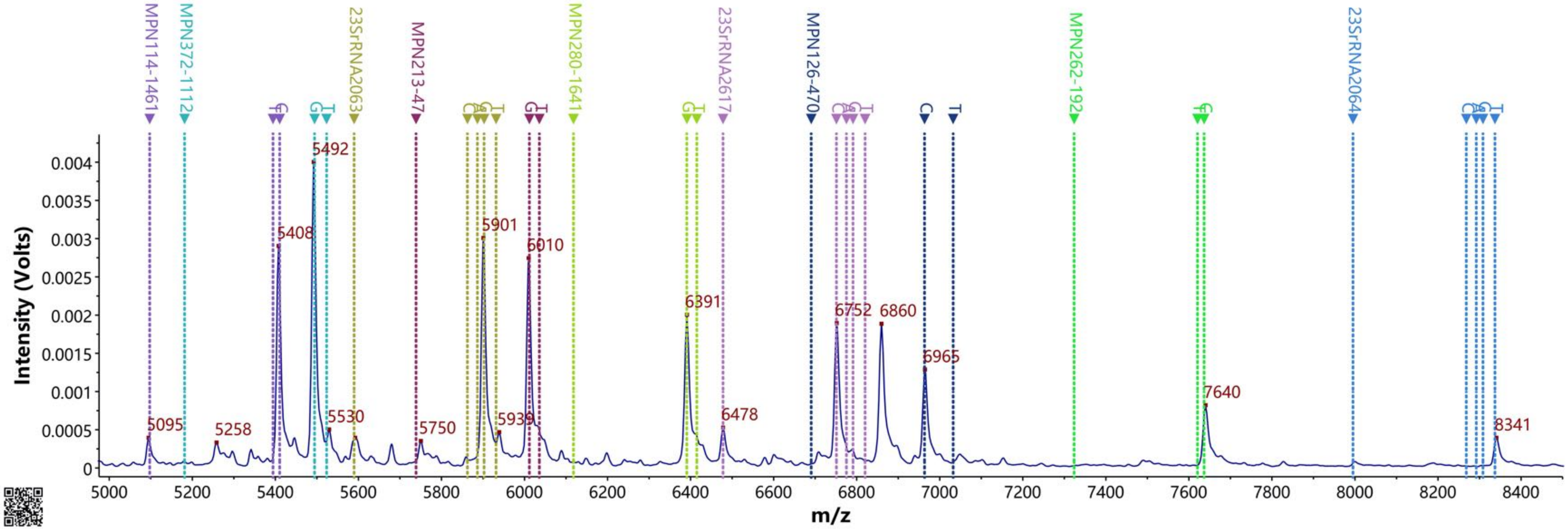

MPN114-1461
MPN372-1112
23SrRNA2063
MPN213-47
MPN280-1641
23SrRNA2617
MPN126-470
MPN262-192
23SrRNA2064
5492
5408
5901
6010
6391
6752
6860
6965
7640
5095
5258
5530
5750
5939
6478
8341
Intensity (Volts)
m/z

Sample-16

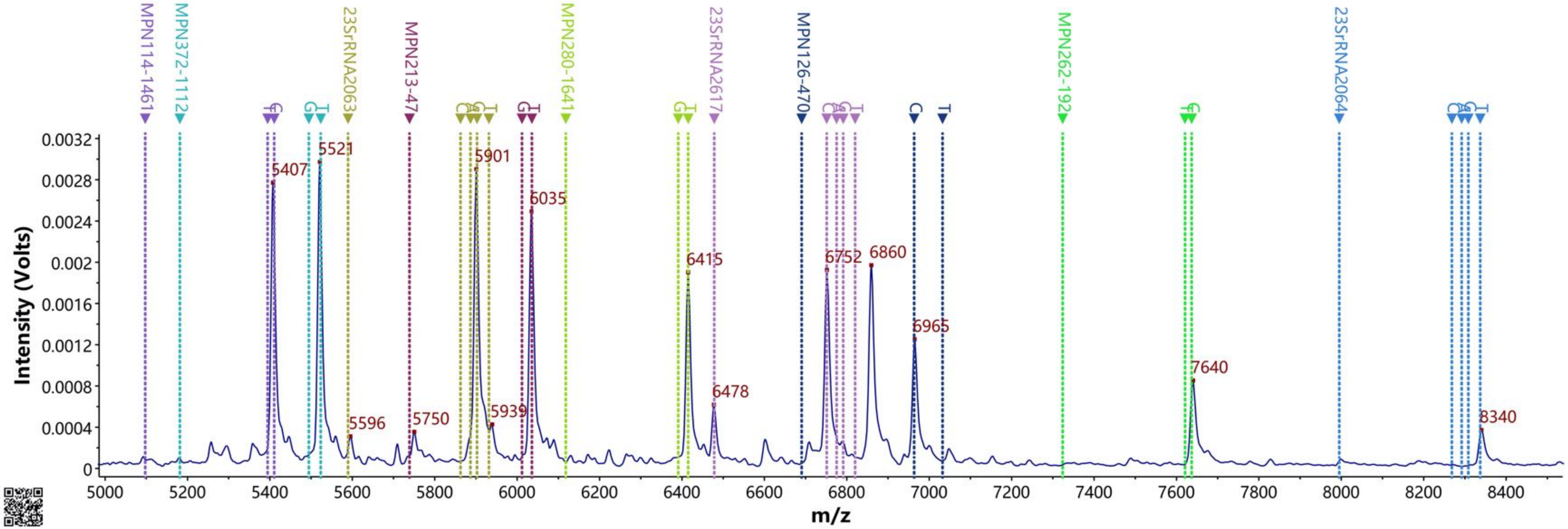

MPN114-1461
MPN372-1112
23SrRNA2063
MPN213-47
MPN280-1641
23SrRNA2617
MPN126-470
MPN262-192
23SrRNA2064
5407
5521
5596
5750
5901
5939
6035
6415
6478
6752
6860
6965
7640
8340
Intensity (Volts)
m/z

Sample-17

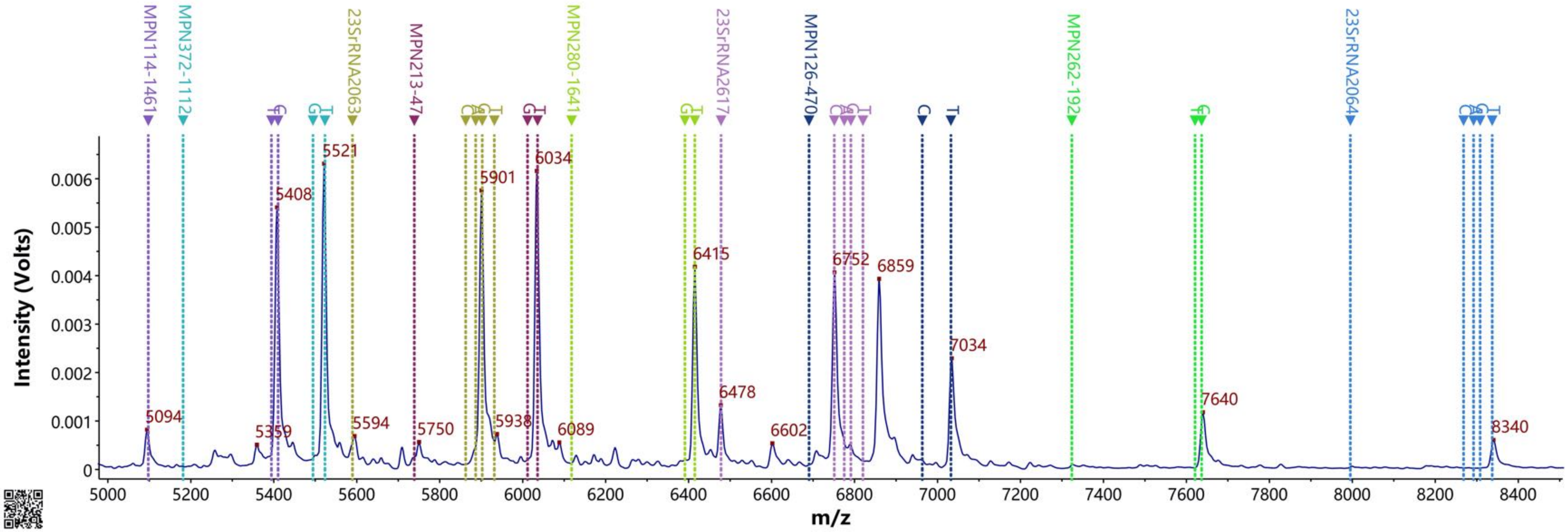

MPN114-1461
MPN372-1112
23SrRNA2063
MPN213-47
MPN280-1641
23SrRNA2617
MPN126-470
MPN262-192
23SrRNA2064
5094
5359
5408
5521
5594
5750
5901
5938
6034
6089
6415
6478
6602
6752
6859
7034
7640
8340
Intensity (Volts)
m/z

Sample-18

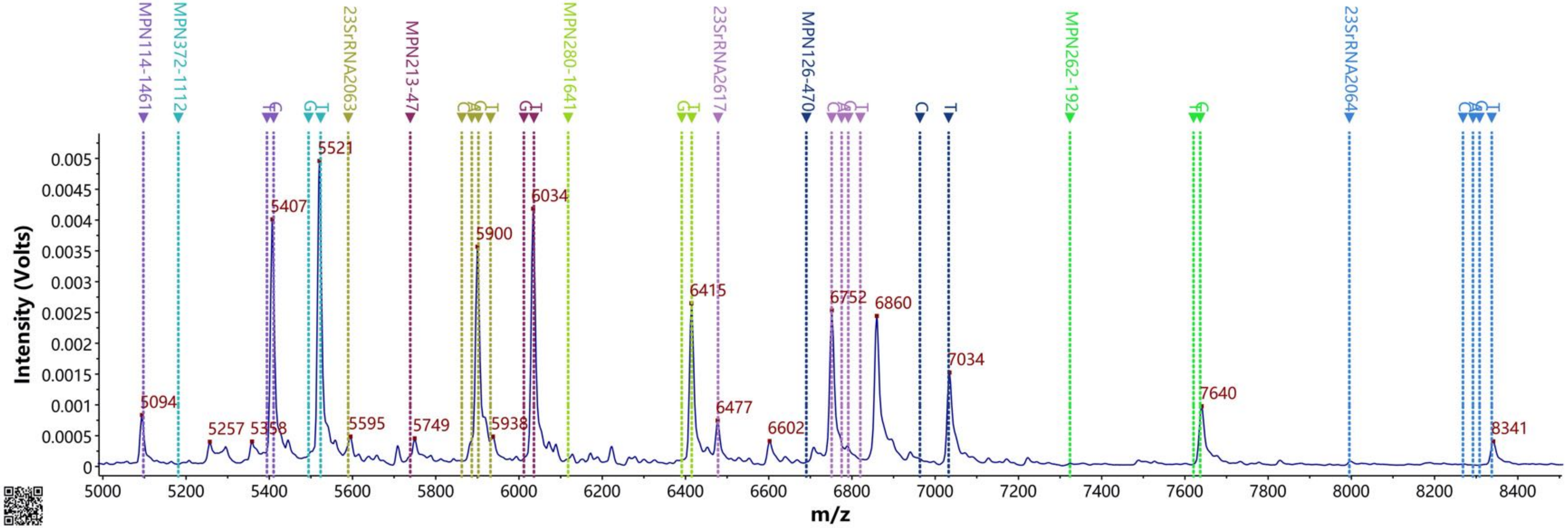

MPN114-1461
MPN372-1112
23SrRNA2063
MPN213-47
MPN280-1641
23SrRNA2617
MPN126-470
MPN262-192
23SrRNA2064
5094
5257
5358
5407
5521
5595
5749
5900
5938
6034
6415
6477
6602
6752
6860
7034
7640
8341
Intensity (Volts)
m/z

Sample-19

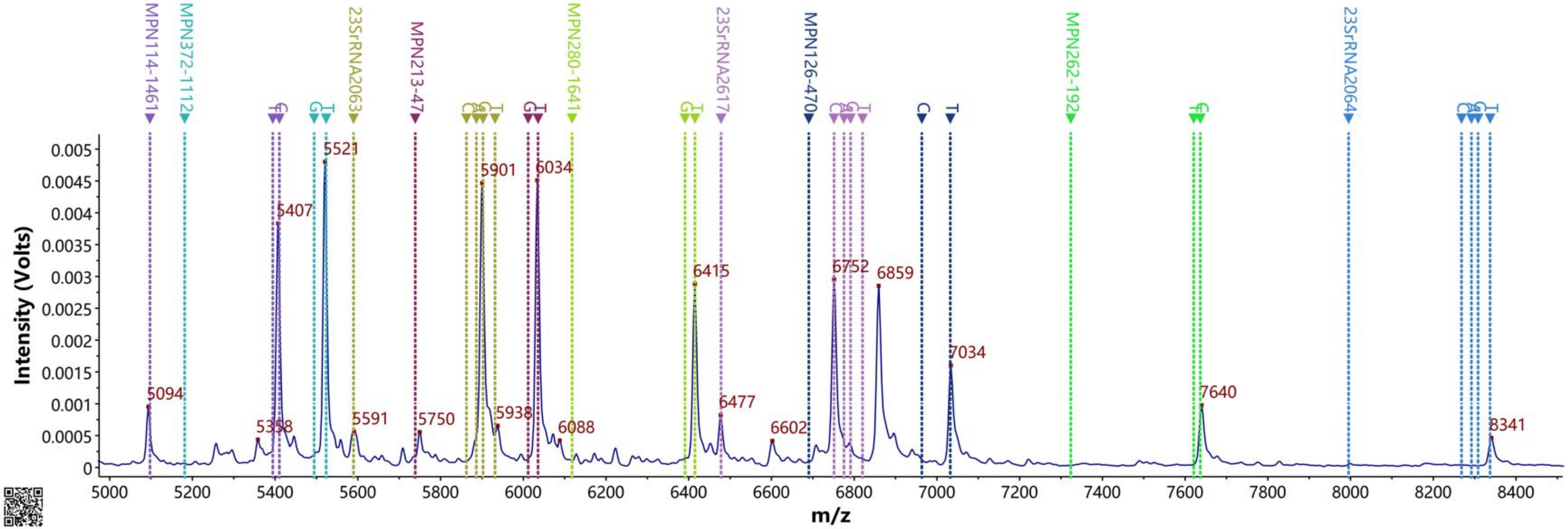

MPN114-1461
MPN372-1112
23SrRNA2063
MPN213-47
MPN280-1641
23SrRNA2617
MPN126-470
MPN262-192
23SrRNA2064
5094
5358
5407
5521
5591
5750
5901
5938
6034
6088
6415
6477
6602
6752
6859
7034
7640
8341
Intensity (Volts)
m/z

Sample-20

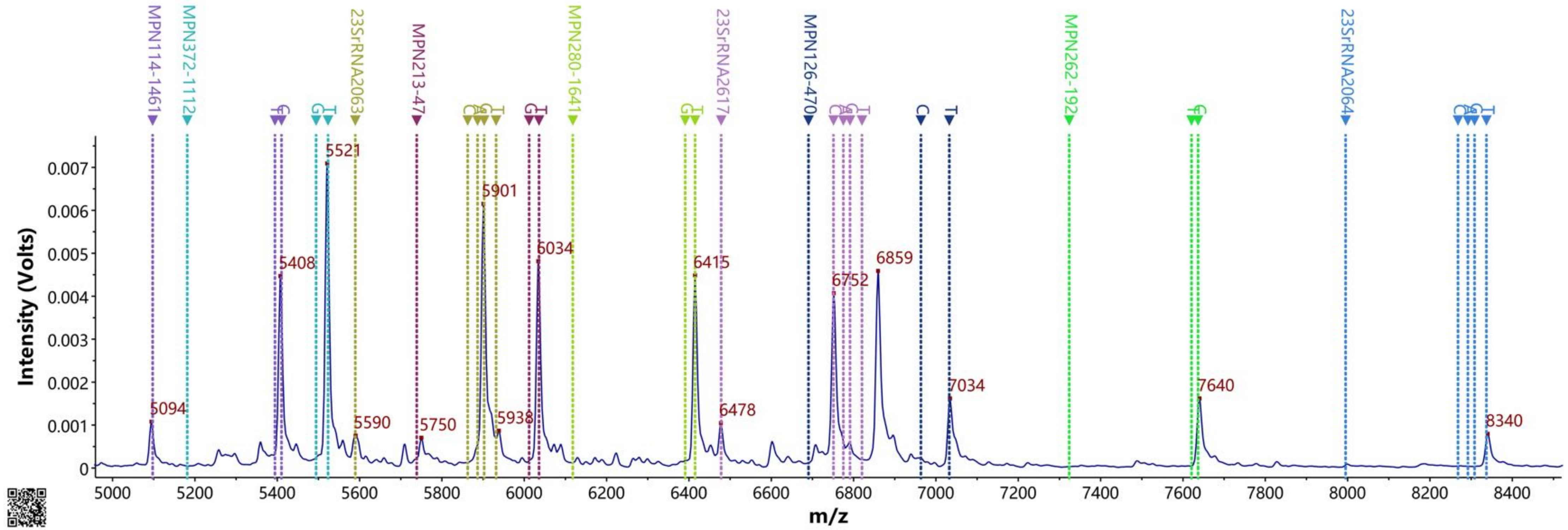

MPN114-1461
MPN372-1112
23SrRNA2063
MPN213-47
MPN280-1641
23SrRNA2617
MPN126-470
MPN262-192
23SrRNA2064
5094
5408
5521
5590
5750
5901
5938
6034
6415
6478
6752
6859
7034
7640
8340
Intensity (Volts)
m/z

Sample-21

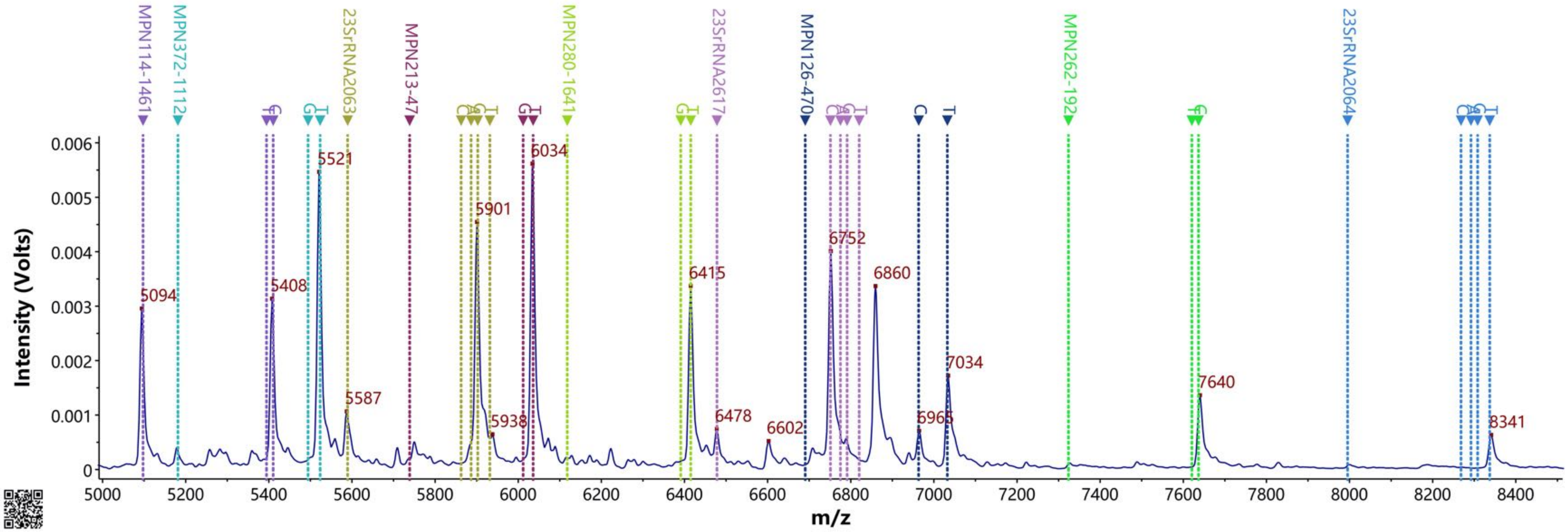

MPN114-1461
MPN372-1112
23SrRNA2063
MPN213-47
MPN280-1641
23SrRNA2617
MPN126-470
MPN262-192
23SrRNA2064
5094
5408
5521
5587
5901
5938
6034
6415
6478
6602
6752
6860
6965
7034
7640
8341
Intensity (Volts)
m/z

Sample-22

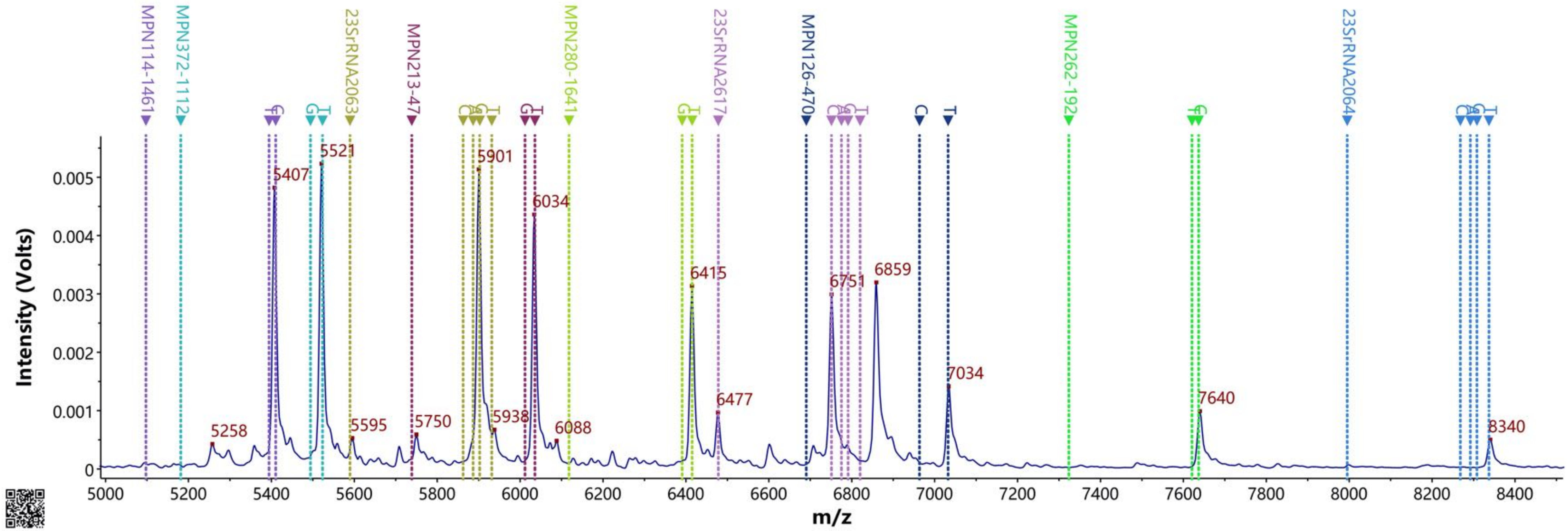

MPN114-1461
MPN372-1112
23SrRNA2063
MPN213-47
MPN280-1641
23SrRNA2617
MPN126-470
MPN262-192
23SrRNA2064
5258
5407
5521
5595
5750
5901
5938
6034
6088
6415
6477
6751
6859
7034
7640
8340
Intensity (Volts)
m/z

Sample-23

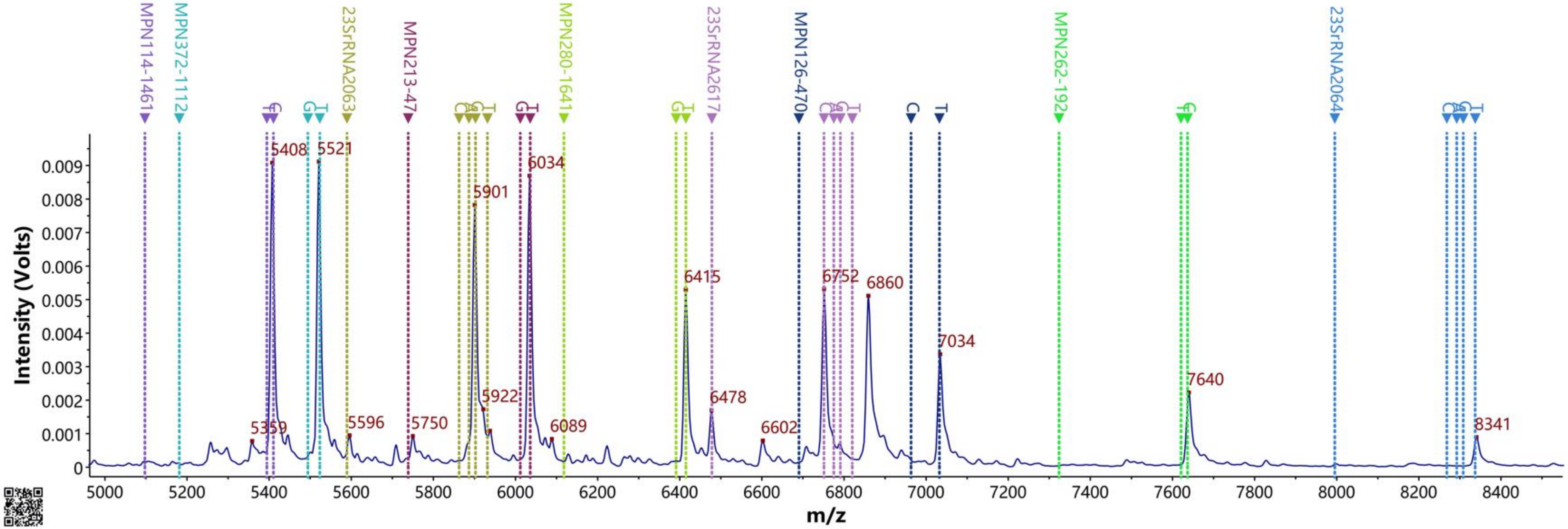

MPN114-1461
MPN372-1112
23SrRNA2063
MPN213-47
MPN280-1641
23SrRNA2617
MPN126-470
MPN262-192
23SrRNA2064
5359
5408
5521
5596
5750
5901
5922
6034
6089
6415
6478
6602
6752
6860
7034
7640
8341
Intensity (Volts)
m/z

Sample-24

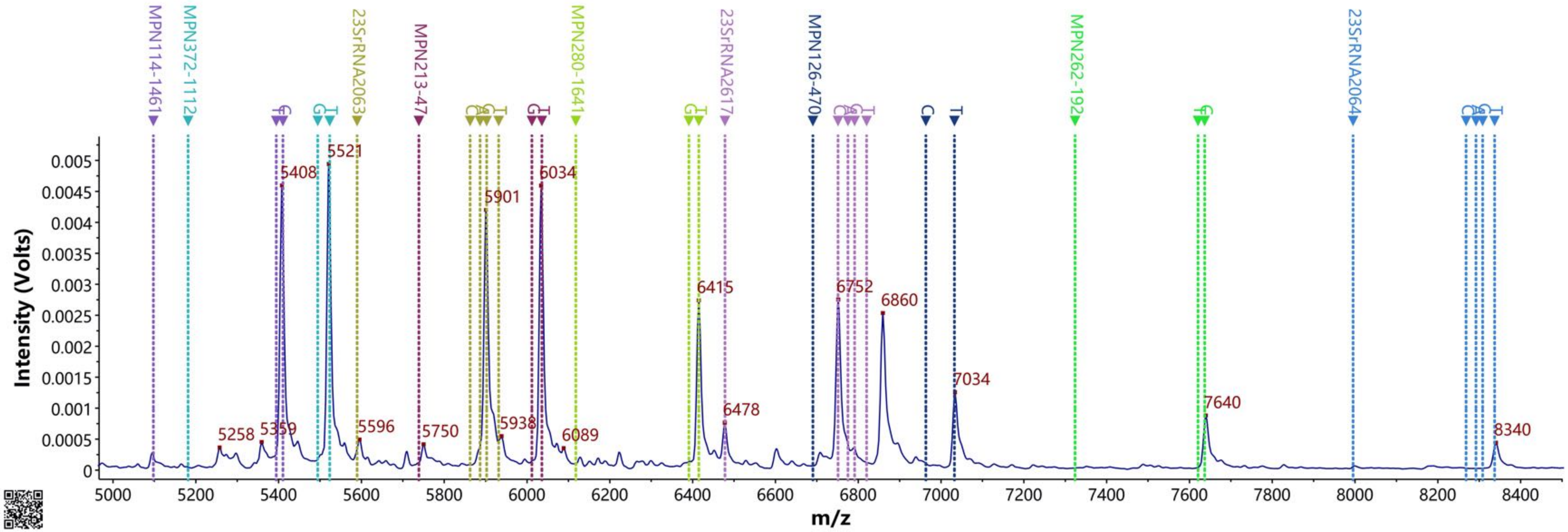
MPN114-1461
MPN372-1112
23SrRNA2063
MPN213-47
MPN280-1641
23SrRNA2617
MPN126-470
MPN262-192
23SrRNA2064
5258
5359
5408
5521
5596
5750
5901
5938
6034
6089
6415
6478
6752
6860
7034
7640
8340
Intensity (Volts)
m/z

Sample-25

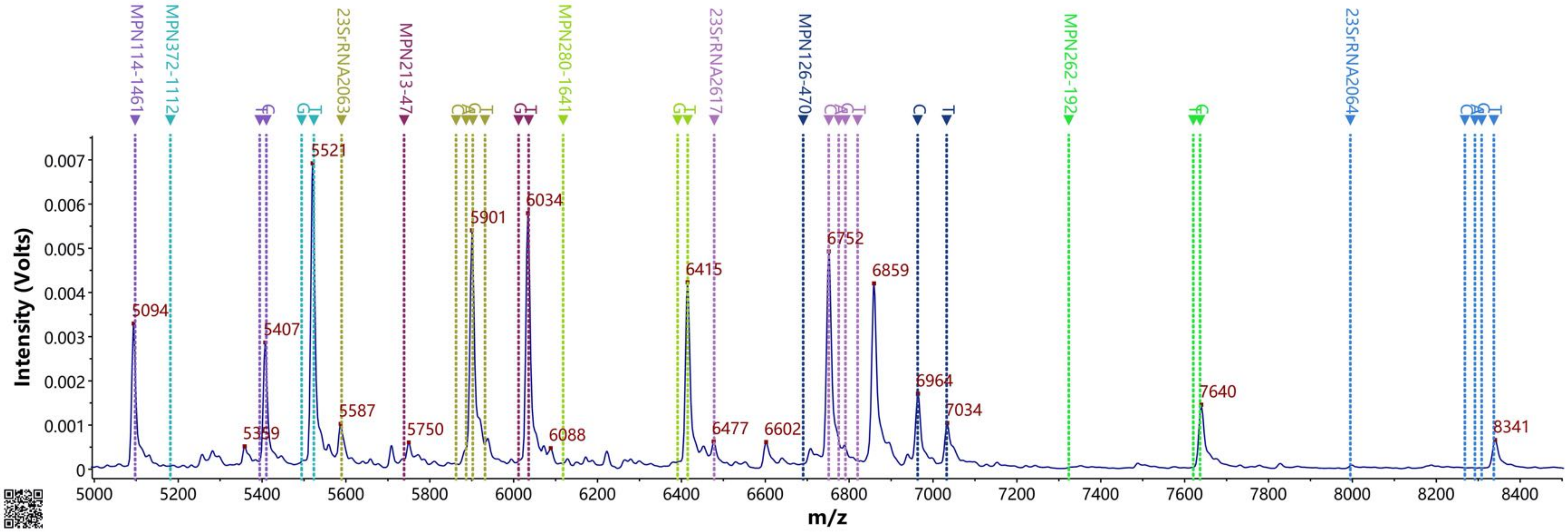

MPN114-1461
MPN372-1112
23SrRNA2063
MPN213-47
MPN280-1641
23SrRNA2617
MPN126-470
MPN262-192
23SrRNA2064
5094
5359
5407
5521
5587
5750
5901
6034
6088
6415
6477
6602
6752
6859
6964
7034
7640
8341
Intensity (Volts)
m/z

Sample-26

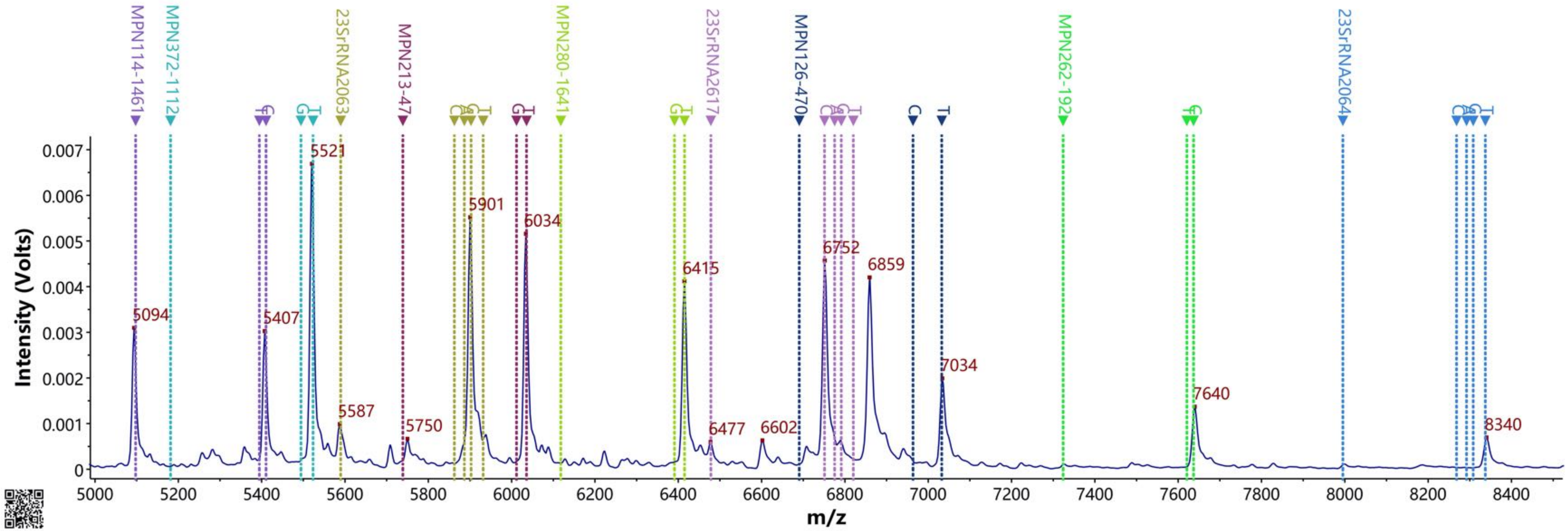

MPN114-1461
MPN372-1112
23SrRNA2063
MPN213-47
MPN280-1641
23SrRNA2617
MPN126-470
MPN262-192
23SrRNA2064
5094
5407
5521
5587
5750
5901
6034
6415
6477
6602
6752
6859
7034
7640
8340
Intensity (Volts)
m/z

Sample-27

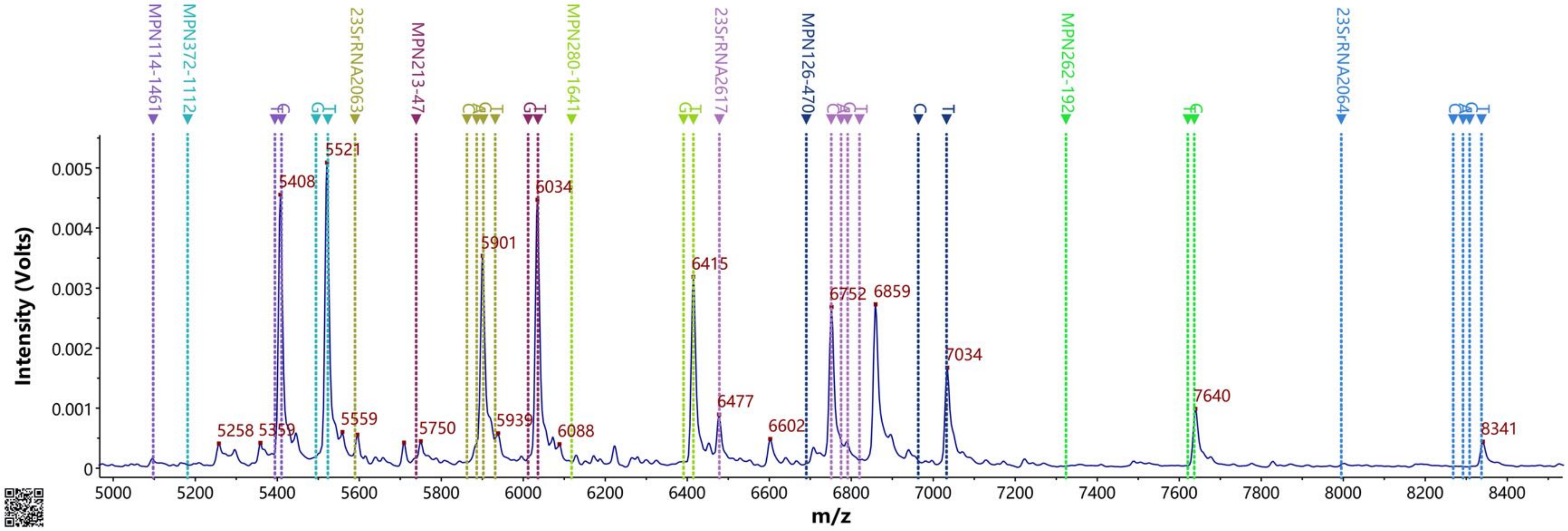
MPN114-1461
MPN372-1112
23SrRNA2063
MPN213-47
MPN280-1641
23SrRNA2617
MPN126-470
MPN262-192
23SrRNA2064
5521
5408
6034
5901
6415
6752
6859
7034
6477
7640
5258
5359
5559
5750
5939
6088
6602
8341
Intensity (Volts)
m/z

Sample-28

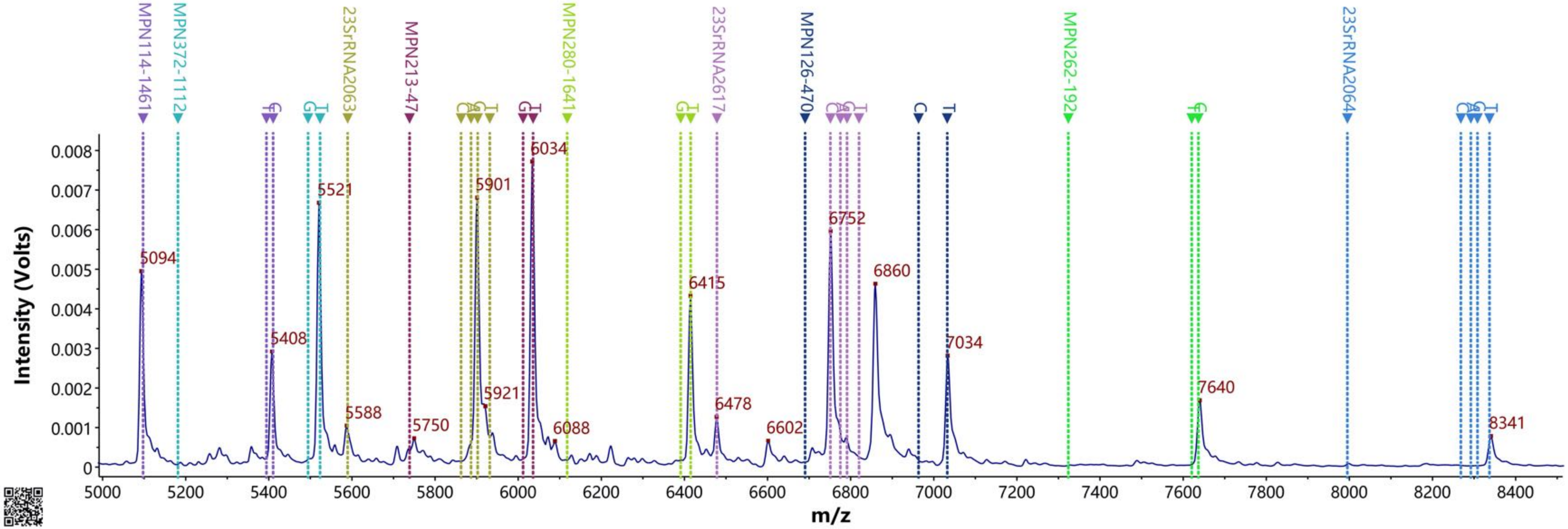

MPN114-1461
MPN372-1112
23SrRNA2063
MPN213-47
MPN280-1641
23SrRNA2617
MPN126-470
MPN262-192
23SrRNA2064
5094
5408
5521
5588
5750
5901
5921
6034
6088
6415
6478
6602
6752
6860
7034
7640
8341
Intensity (Volts)
m/z

Sample-29

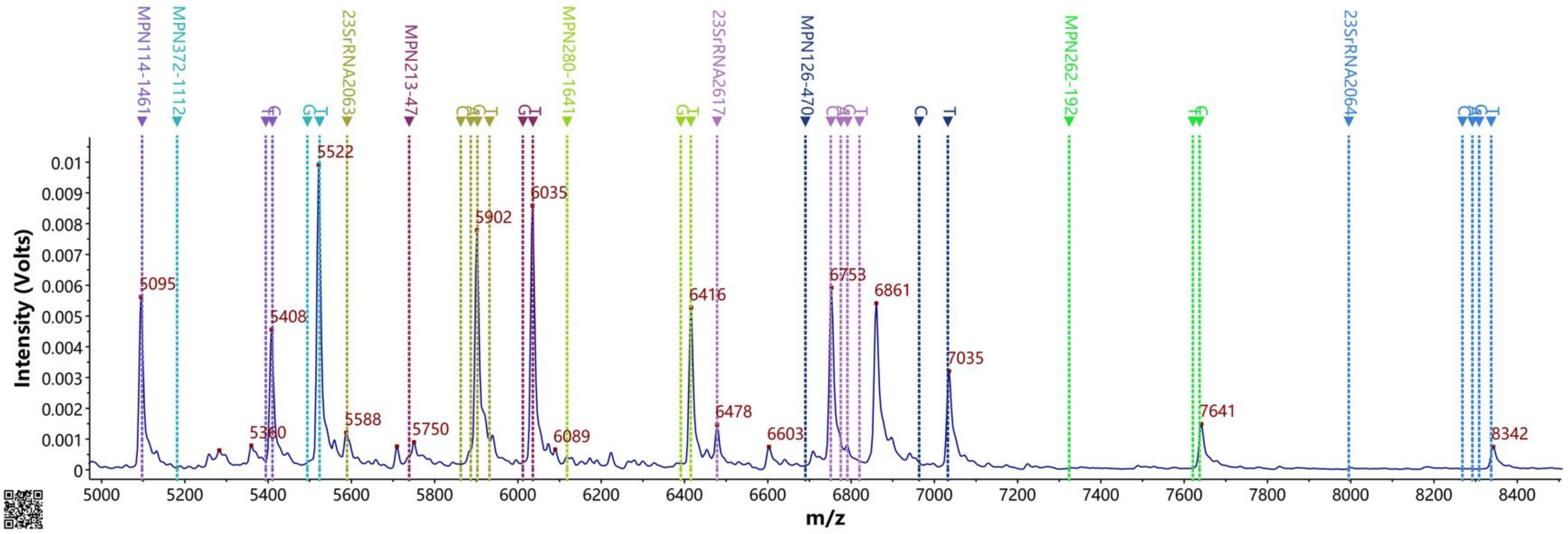

MPN114-1461
MPN372-1112
23SrRNA2063
MPN213-47
MPN280-1641
23SrRNA2617
MPN126-470
MPN262-192
23SrRNA2064
5095
5360
5408
5522
5588
5750
5902
6035
6089
6416
6478
6603
6753
6861
7035
7641
8342
Intensity (Volts)
m/z

Sample-30

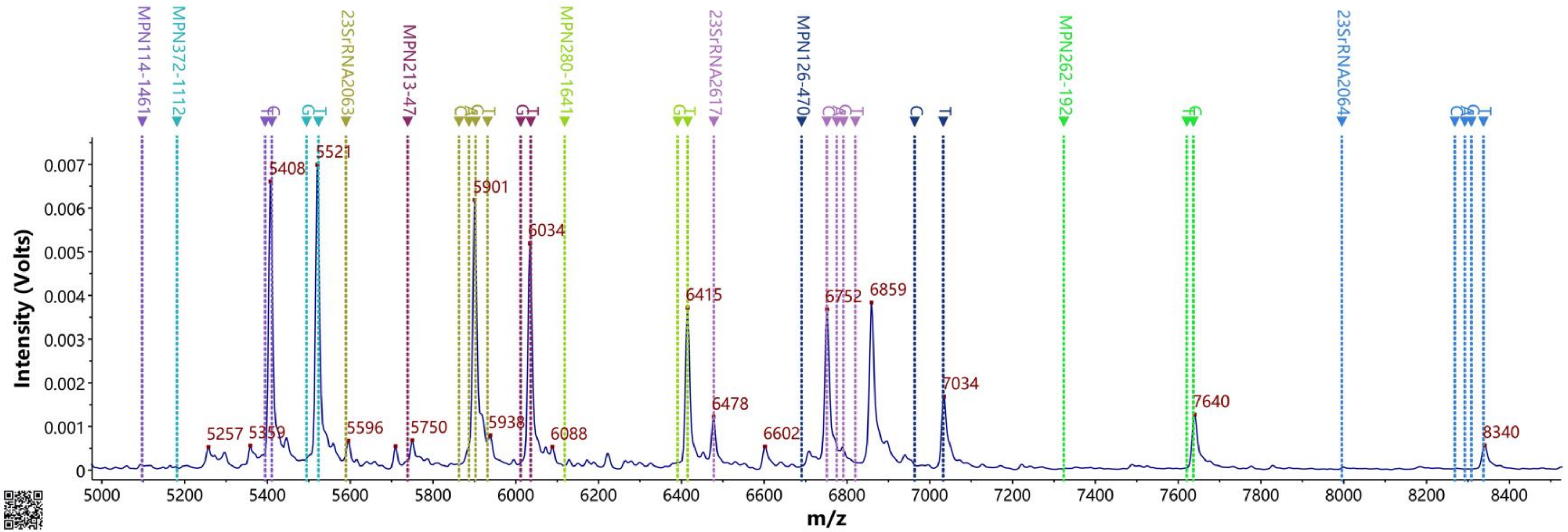

MPN114-1461
MPN372-1112
23SrRNA2063
MPN213-47
MPN280-1641
23SrRNA2617
MPN126-470
MPN262-192
23SrRNA2064
Intensity (Volts)
m/z
5257
5359
5408
5521
5596
5750
5901
5938
6034
6088
6415
6478
6602
6752
6859
7034
7640
8340

Sample-31

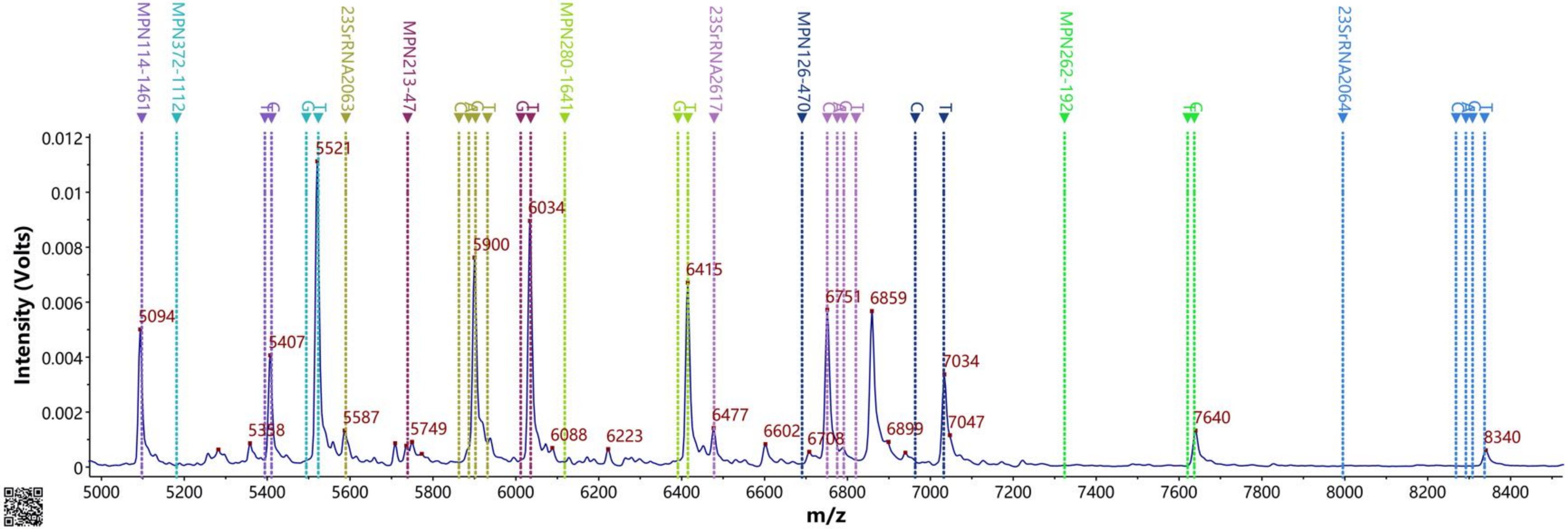

MPN114-1461
MPN372-1112
23SrRNA2063
MPN213-47
MPN280-1641
23SrRNA2617
MPN126-470
MPN262-192
23SrRNA2064
Intensity (Volts)
m/z
5094
5407
5521
5587
5749
5900
6034
6088
6223
6415
6477
6602
6751
6859
6899
7034
7047
7640
8340

Sample-32

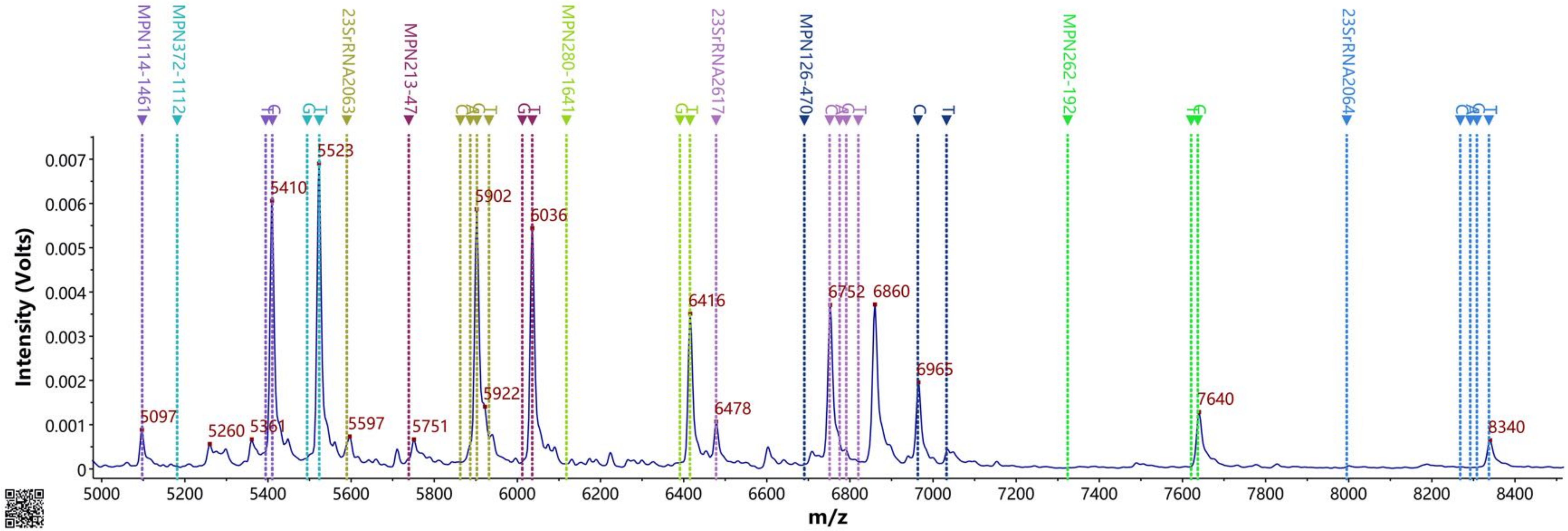

MPN114-1461
MPN372-1112
23SrRNA2063
MPN213-47
MPN280-1641
23SrRNA2617
MPN126-470
MPN262-192
23SrRNA2064
5097
5260
5361
5410
5523
5597
5751
5902
5922
6036
6416
6478
6752
6860
6965
7640
8340
Intensity (Volts)
m/z

Sample-33

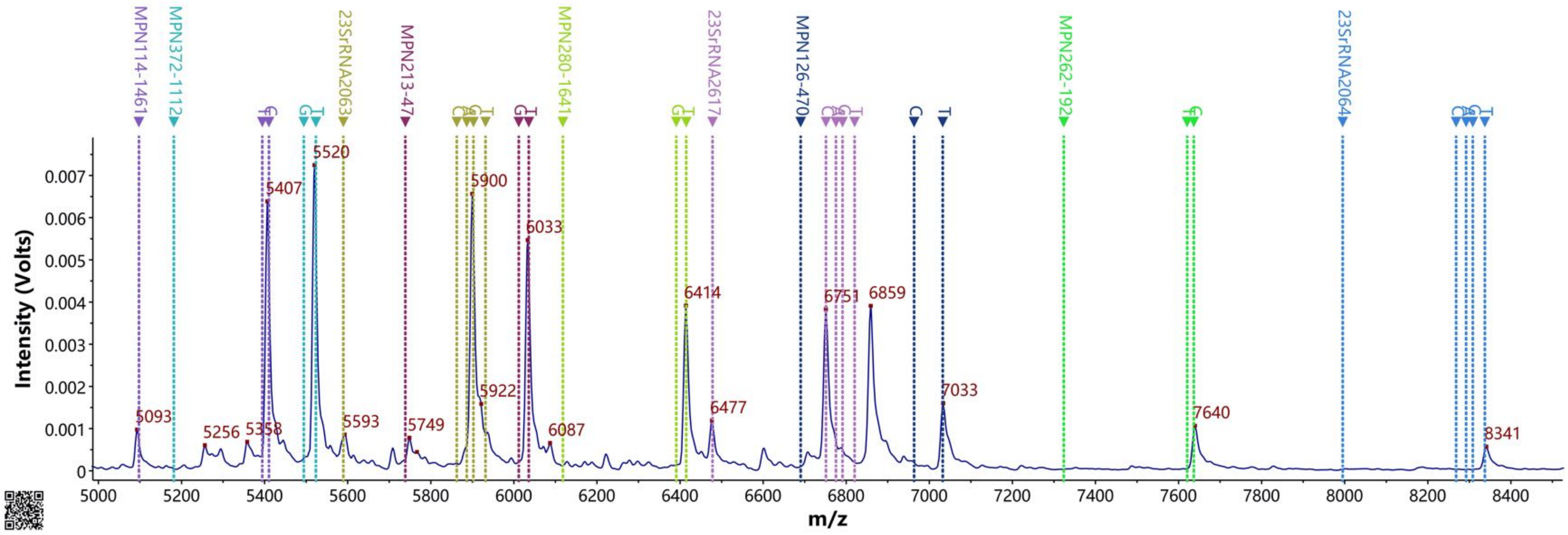
MPN114-1461
MPN372-1112
23SrRNA2063
MPN213-47
MPN280-1641
23SrRNA2617
MPN126-470
MPN262-192
23SrRNA2064
5520
5407
5900
6033
6414
6751
6859
5922
6477
7033
7640
8341
5093
5256
5358
5593
5749
6087
Intensity (Volts)
m/z

Sample-34

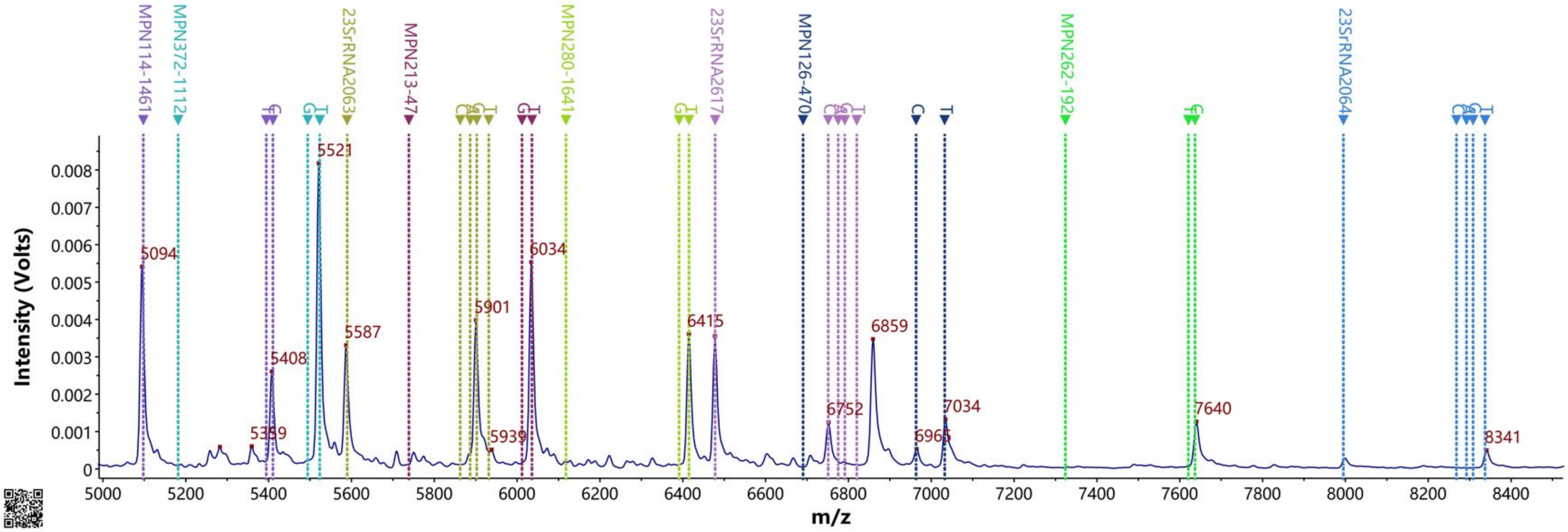

MPN114-1461
MPN372-1112
23SrRNA2063
MPN213-47
MPN280-1641
23SrRNA2617
MPN126-470
MPN262-192
23SrRNA2064
5094
5359
5408
5521
5587
5901
5939
6034
6415
6752
6859
6965
7034
7640
8341
Intensity (Volts)
m/z

Sample-35

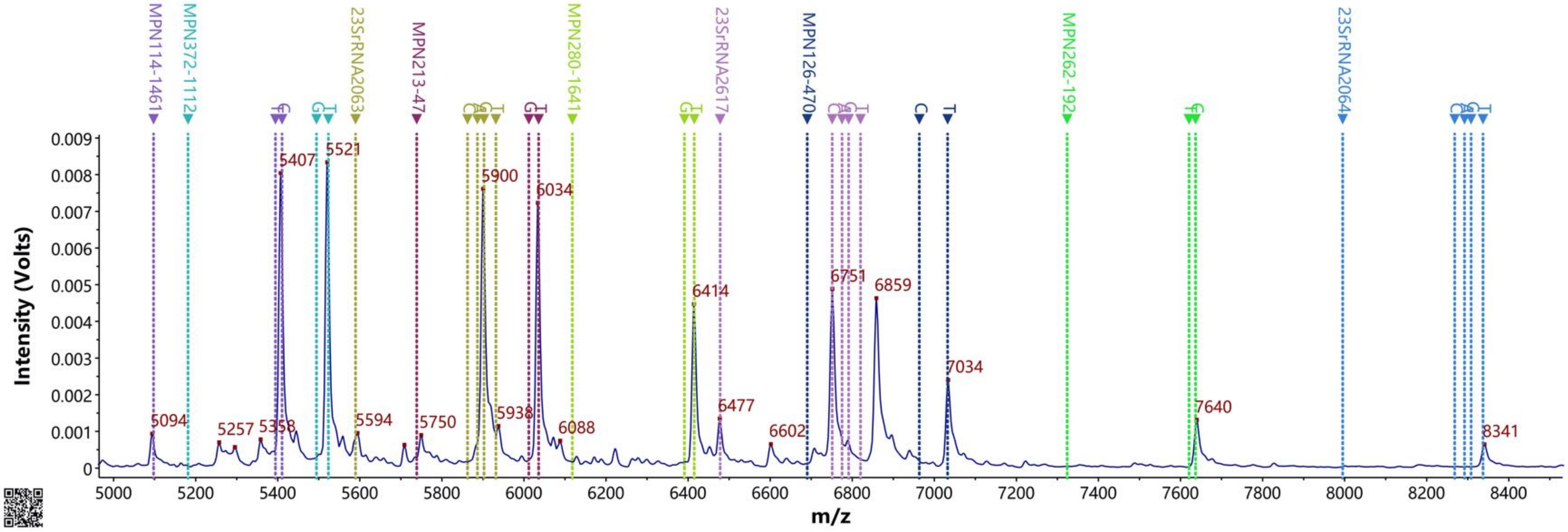
MPN114-1461
MPN372-1112
23SrRNA2063
MPN213-47
MPN280-1641
23SrRNA2617
MPN126-470
MPN262-192
23SrRNA2064
Intensity (Volts)
m/z
5094
5257
5358
5407
5521
5594
5750
5900
5938
6034
6088
6414
6477
6602
6751
6859
7034
7640
8341

Sample-36

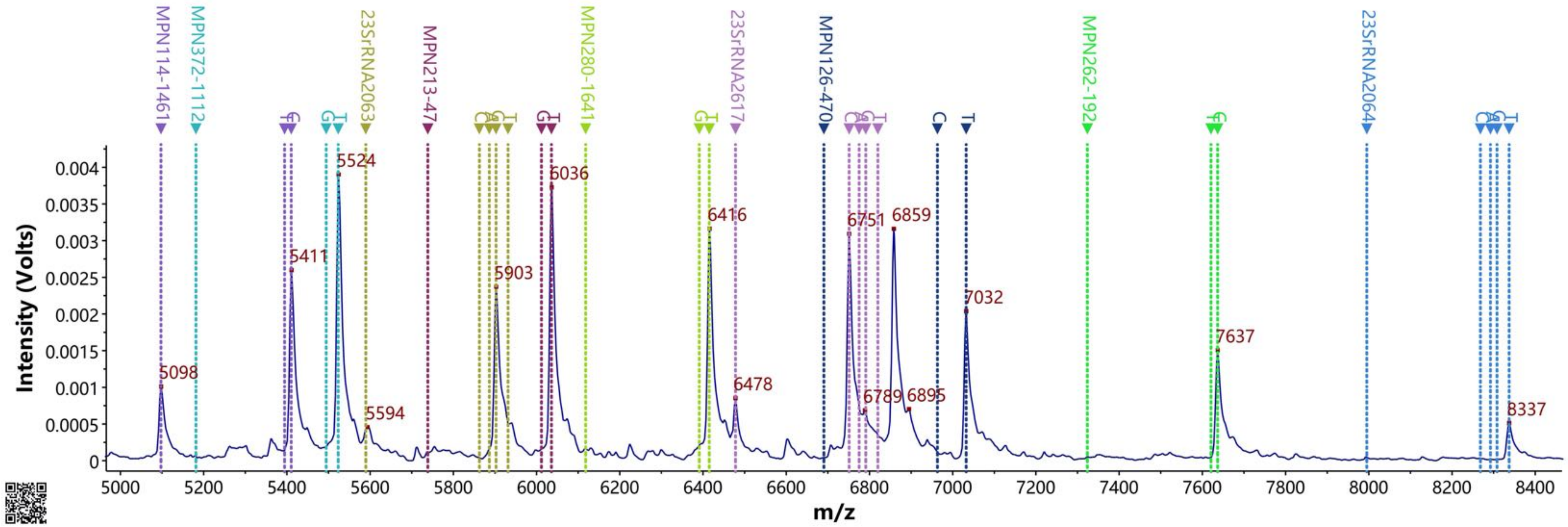

MPN114-1461
MPN372-1112
23SrRNA2063
MPN213-47
MPN280-1641
23SrRNA2617
MPN126-470
MPN262-192
23SrRNA2064
5098
5411
5524
5594
5903
6036
6416
6478
6751
6789
6859
6895
7032
7637
8337
Intensity (Volts)
m/z

Sample-37

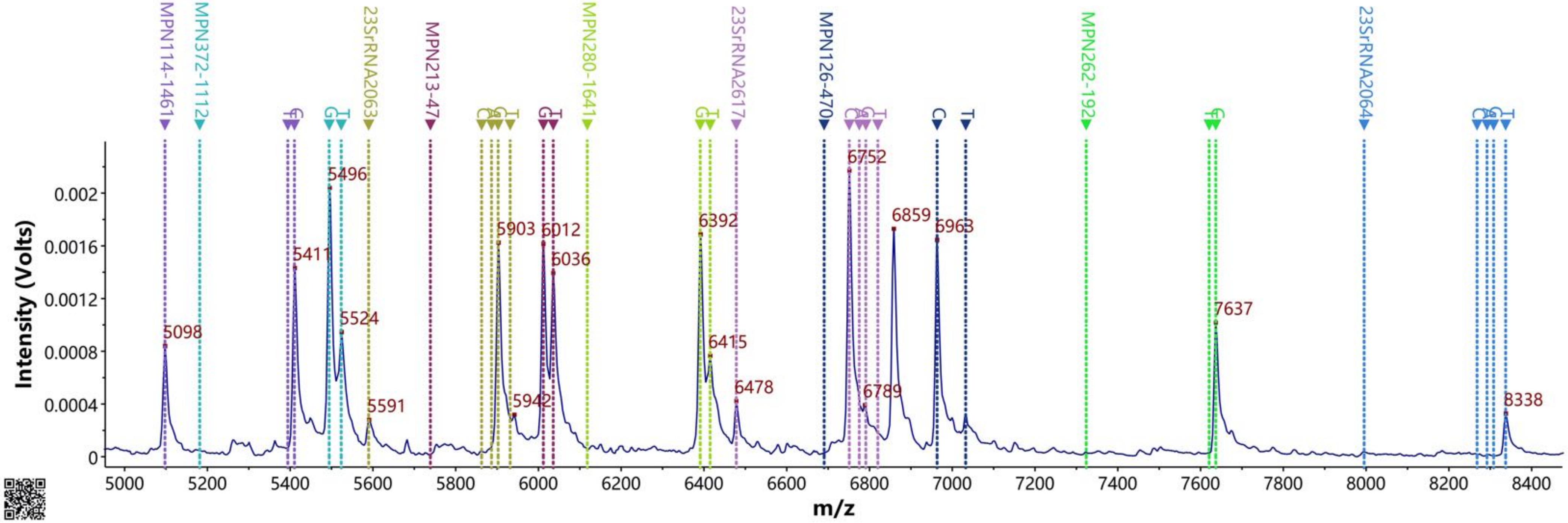

MPN114-1461
MPN372-1112
23SrRNA2063
MPN213-47
MPN280-1641
23SrRNA2617
MPN126-470
MPN262-192
23SrRNA2064
5098
5411
5496
5524
5591
5903
5942
6012
6036
6392
6415
6478
6752
6789
6859
6963
7637
8338
Intensity (Volts)
m/z

Sample-38

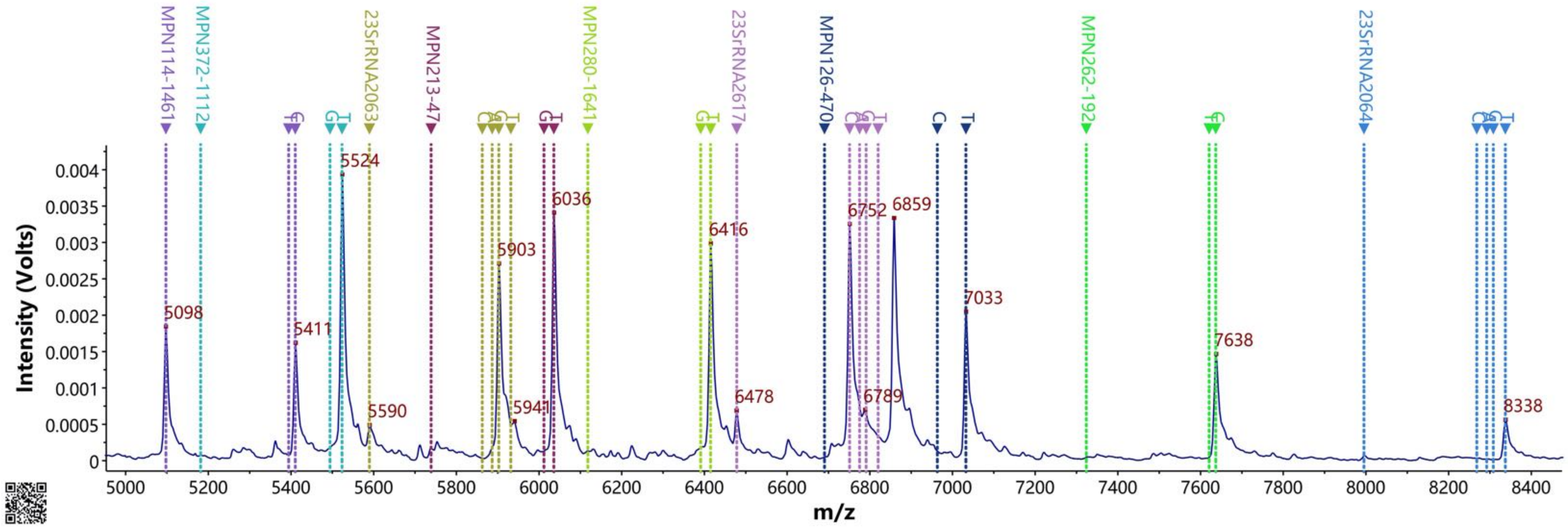

MPN114-1461
MPN372-1112
23SrRNA2063
MPN213-47
MPN280-1641
23SrRNA2617
MPN126-470
MPN262-192
23SrRNA2064
5098
5411
5524
5590
5903
5941
6036
6416
6478
6752
6789
6859
7033
7638
8338
Intensity (Volts)
m/z

Sample-39

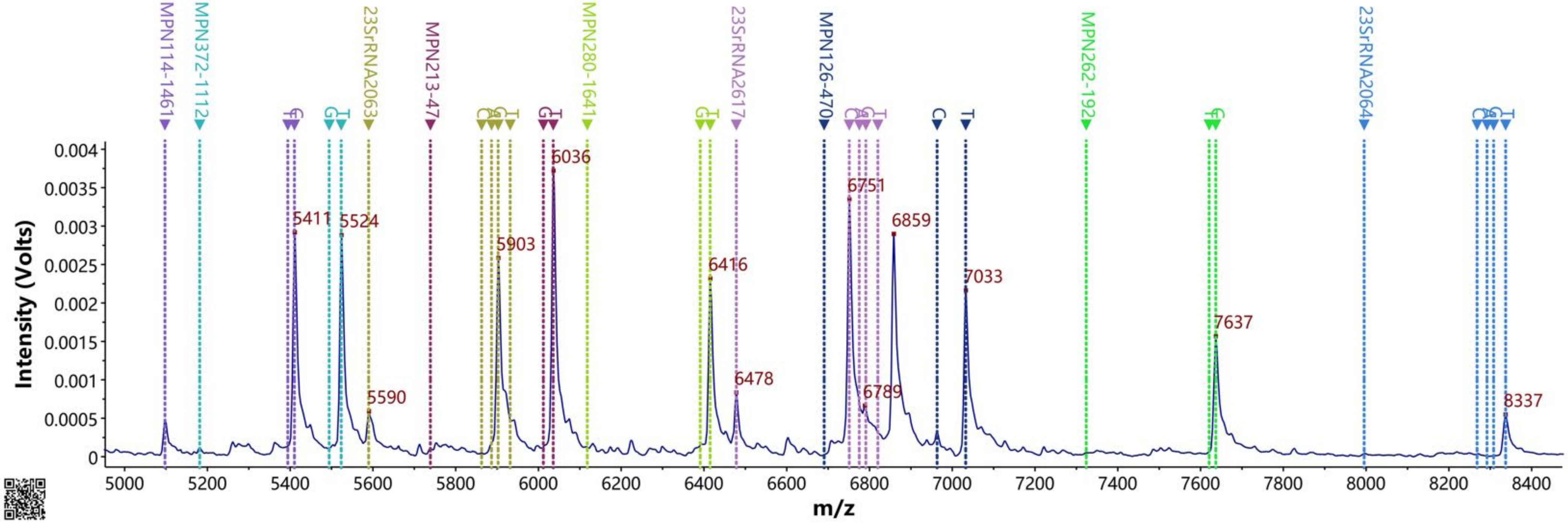
MPN114-1461
MPN372-1112
23SrRNA2063
MPN213-47
MPN280-1641
23SrRNA2617
MPN126-470
MPN262-192
23SrRNA2064
5411
5524
5590
5903
6036
6416
6478
6751
6789
6859
7033
7637
8337
Intensity (Volts)
m/z

Sample-40

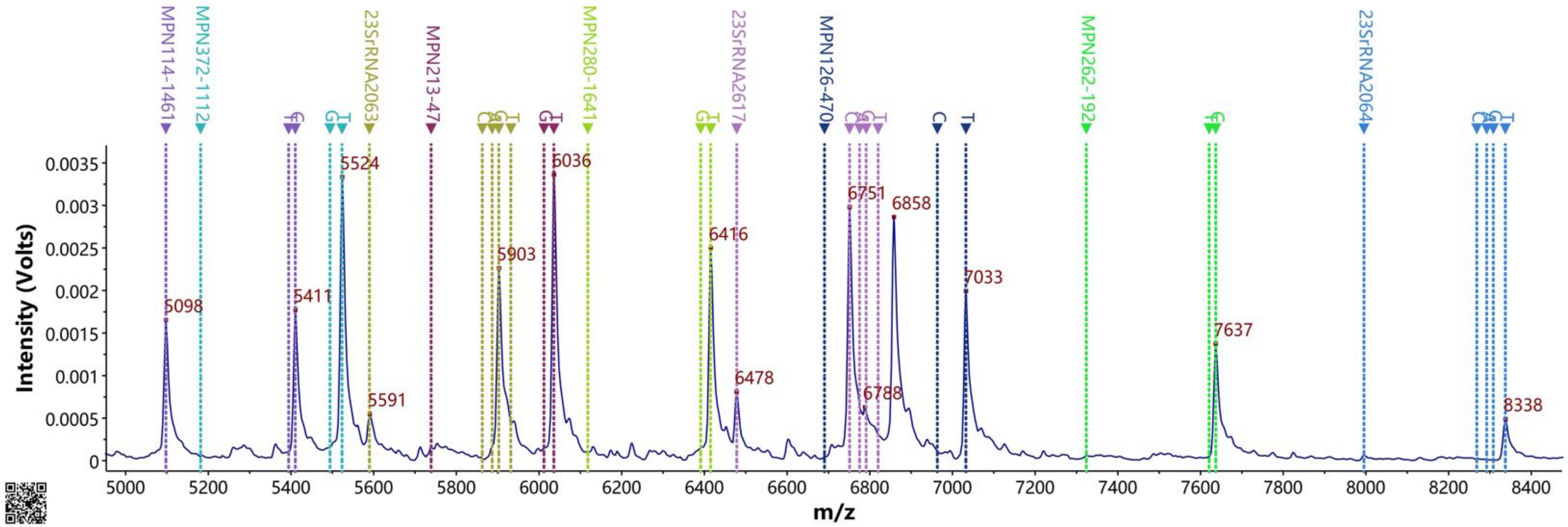

MPN114-1461
MPN372-1112
23SrRNA2063
MPN213-47
MPN280-1641
23SrRNA2617
MPN126-470
MPN262-192
23SrRNA2064
5098
5411
5524
5591
5903
6036
6416
6478
6751
6788
6858
7033
7637
8338
Intensity (Volts)
m/z

Sample-41

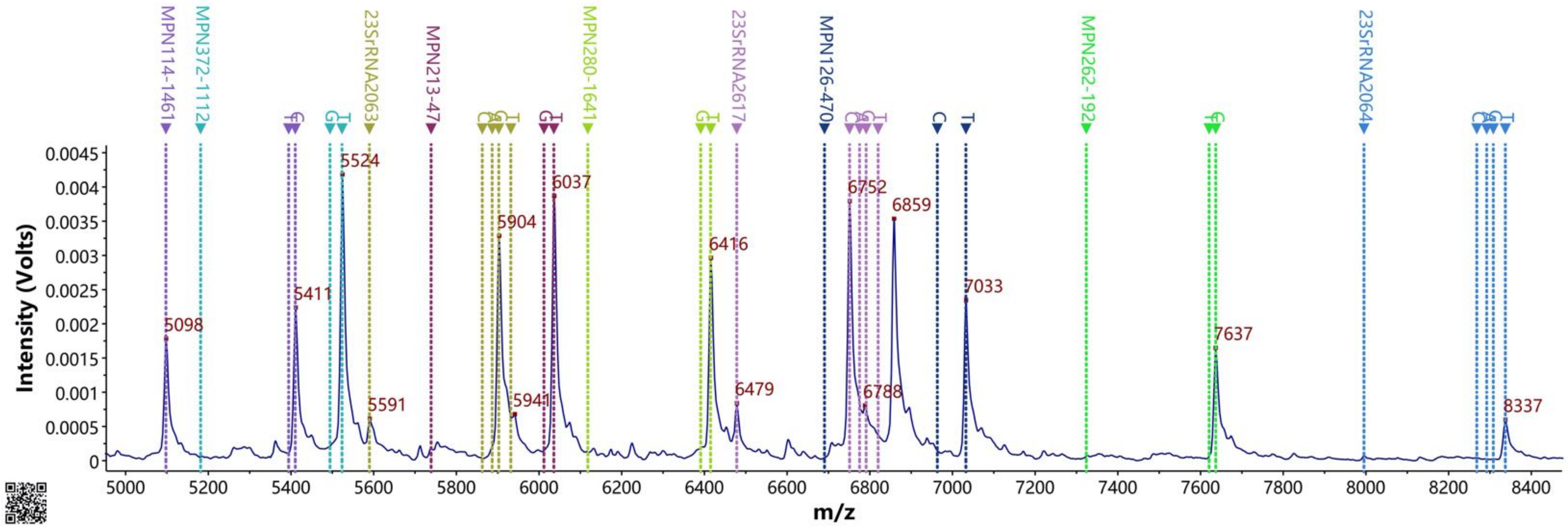

MPN114-1461
MPN372-1112
23SrRNA2063
MPN213-47
MPN280-1641
23SrRNA2617
MPN126-470
MPN262-192
23SrRNA2064
5098
5411
5524
5591
5904
5941
6037
6416
6479
6752
6788
6859
7033
7637
8337
Intensity (Volts)
m/z

Sample-42

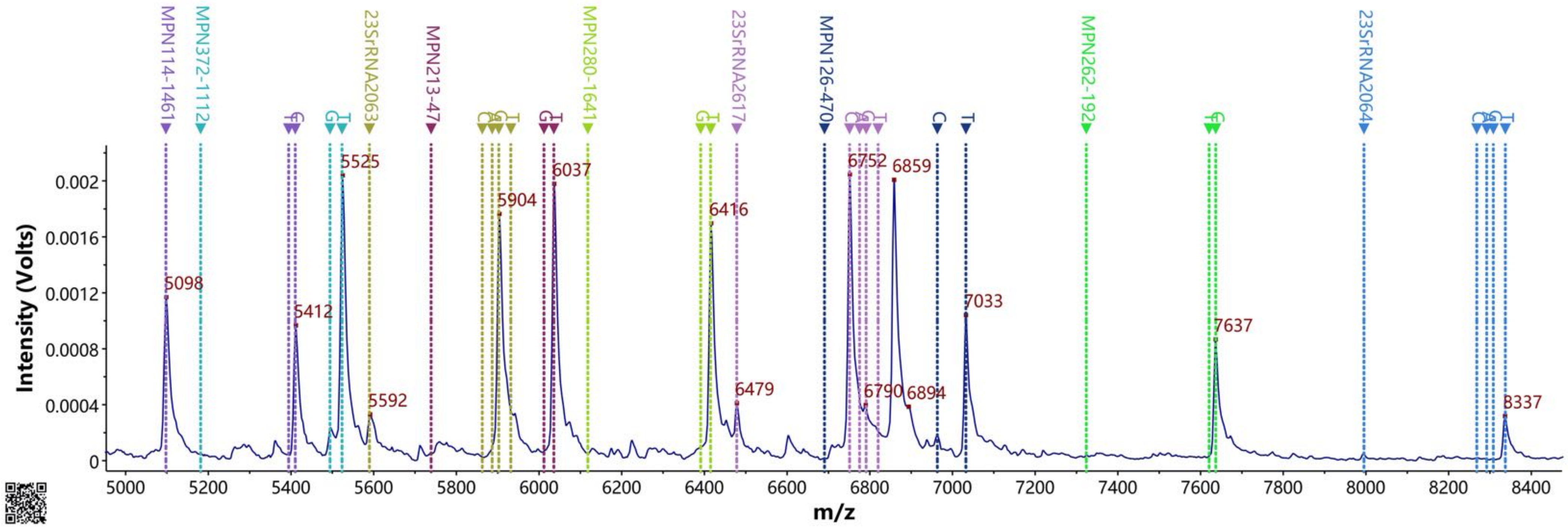

MPN114-1461
MPN372-1112
23SrRNA2063
MPN213-47
MPN280-1641
23SrRNA2617
MPN126-470
MPN262-192
23SrRNA2064
5098
5412
5525
5592
5904
6037
6416
6479
6752
6790
6859
6894
7033
7637
8337
Intensity (Volts)
m/z

Sample-43

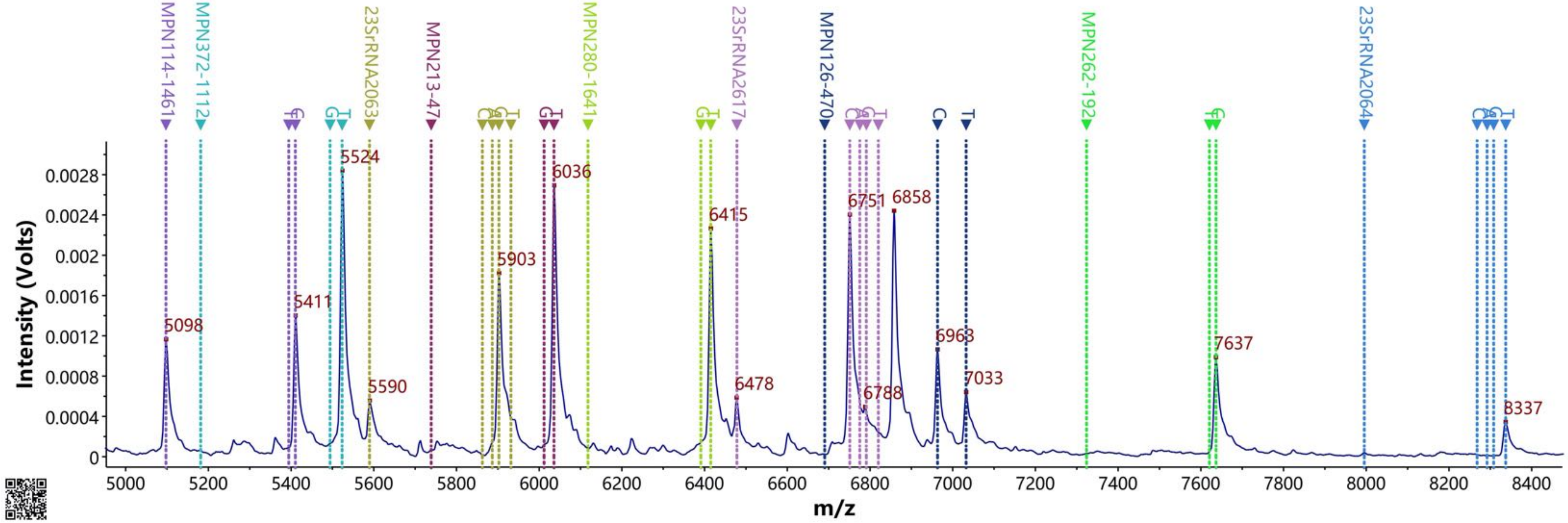

MPN114-1461
MPN372-1112
23SrRNA2063
MPN213-47
MPN280-1641
23SrRNA2617
MPN126-470
MPN262-192
23SrRNA2064
5098
5411
5524
5590
5903
6036
6415
6478
6751
6788
6858
6963
7033
7637
8337
Intensity (Volts)
m/z

Sample-44

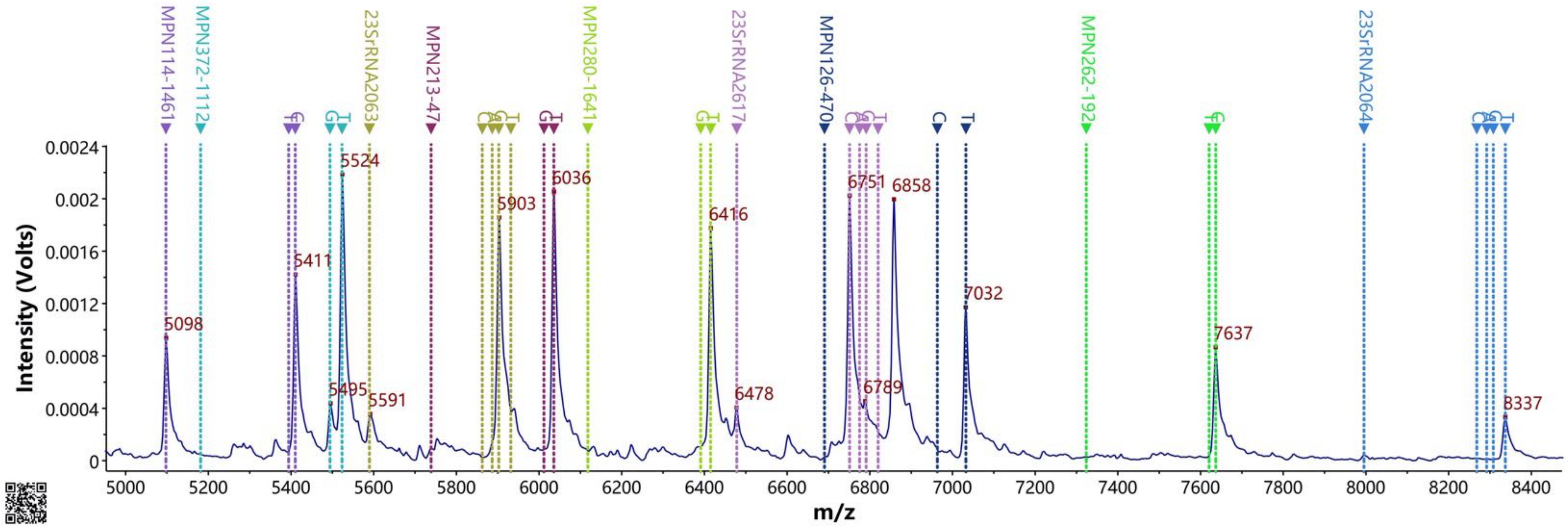

MPN114-1461
MPN372-1112
23SrRNA2063
MPN213-47
MPN280-1641
23SrRNA2617
MPN126-470
MPN262-192
23SrRNA2064
Intensity (Volts)
m/z
5098
5411
5495
5524
5591
5903
6036
6416
6478
6751
6789
6858
7032
7637
8337

Sample-45

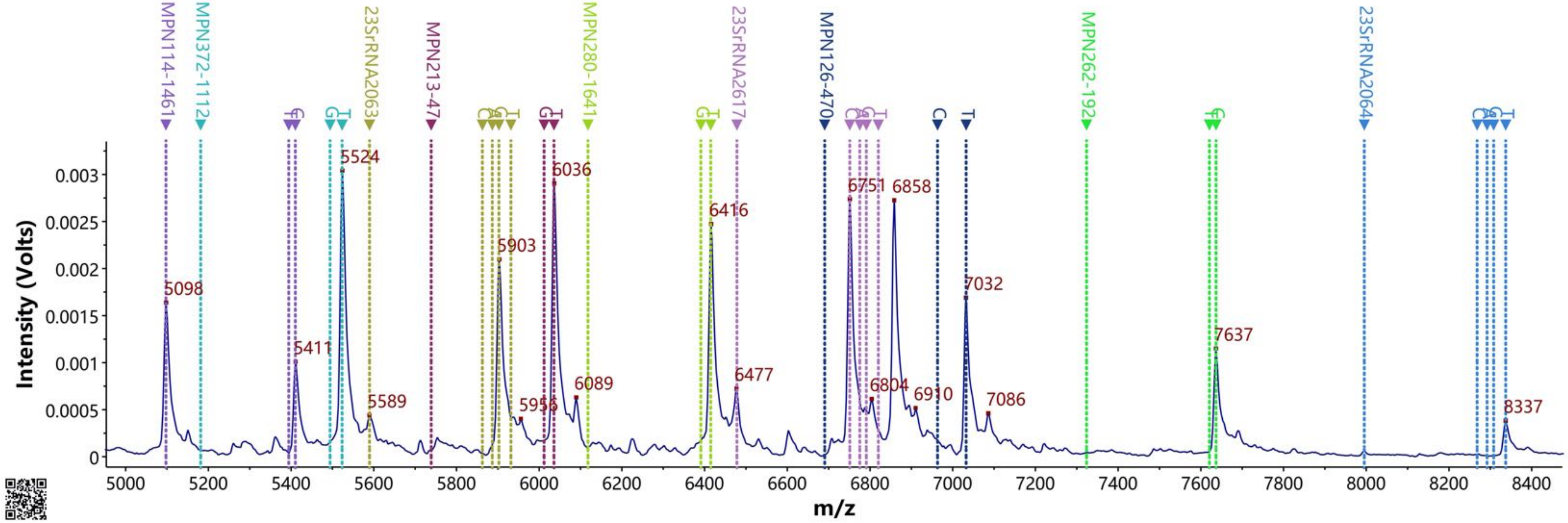

MPN114-1461
MPN372-1112
23SrRNA2063
MPN213-47
MPN280-1641
23SrRNA2617
MPN126-470
MPN262-192
23SrRNA2064
5098
5411
5524
5589
5903
5956
6036
6089
6416
6477
6751
6804
6858
6910
7032
7086
7637
8337
Intensity (Volts)
m/z

Sample-46

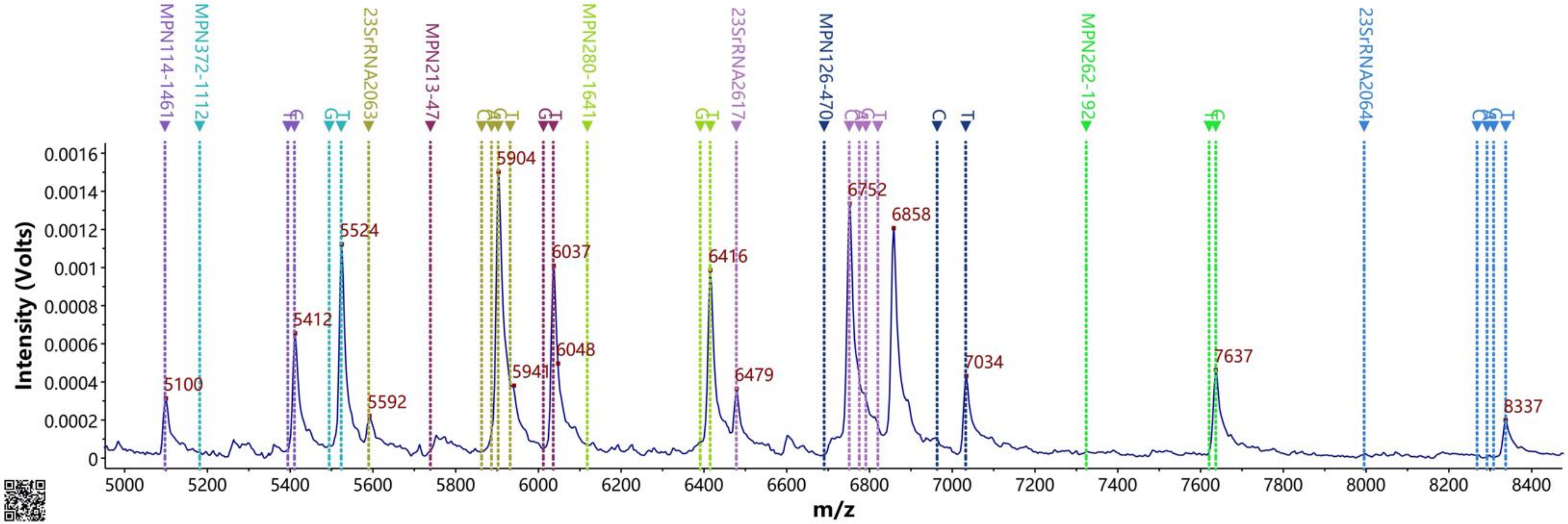
MPN114-1461
MPN372-1112
23SrRNA2063
MPN213-47
MPN280-1641
23SrRNA2617
MPN126-470
MPN262-192
23SrRNA2064
5100
5412
5524
5592
5904
5941
6037
6048
6416
6479
6752
6858
7034
7637
8337
Intensity (Volts)
m/z

Sample-47

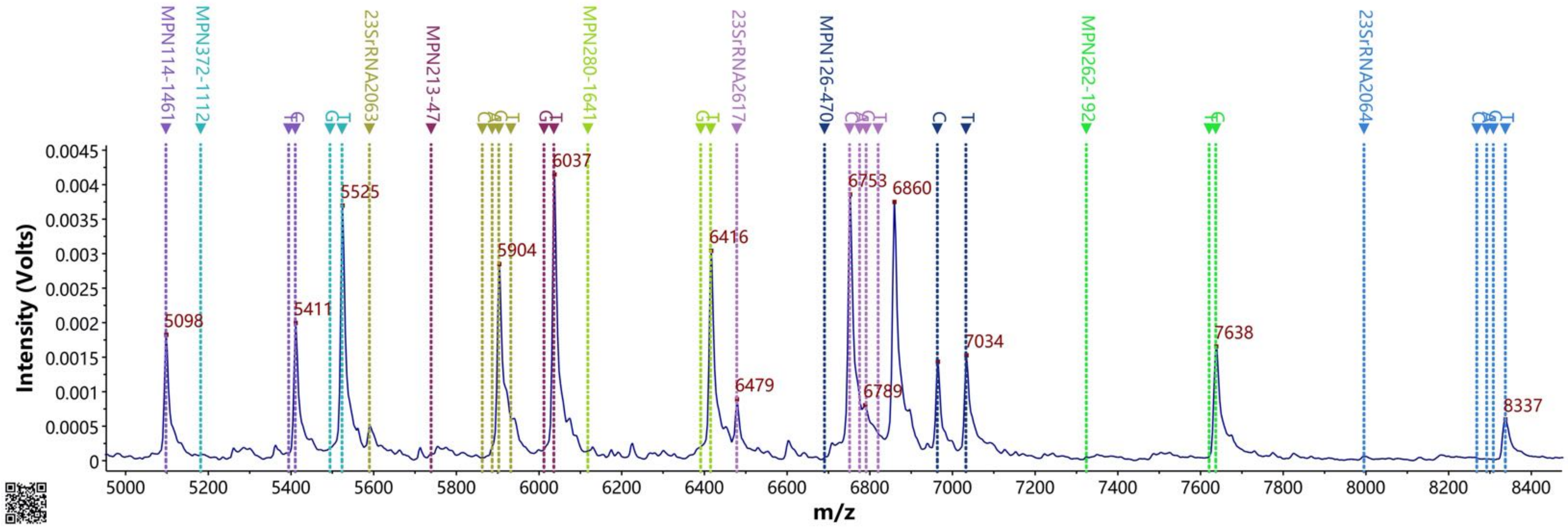

MPN114-1461
MPN372-1112
23SrRNA2063
MPN213-47
MPN280-1641
23SrRNA2617
MPN126-470
MPN262-192
23SrRNA2064
Intensity (Volts)
m/z
5098
5411
5525
5904
6037
6416
6479
6753
6789
6860
7034
7638
8337

Sample-48

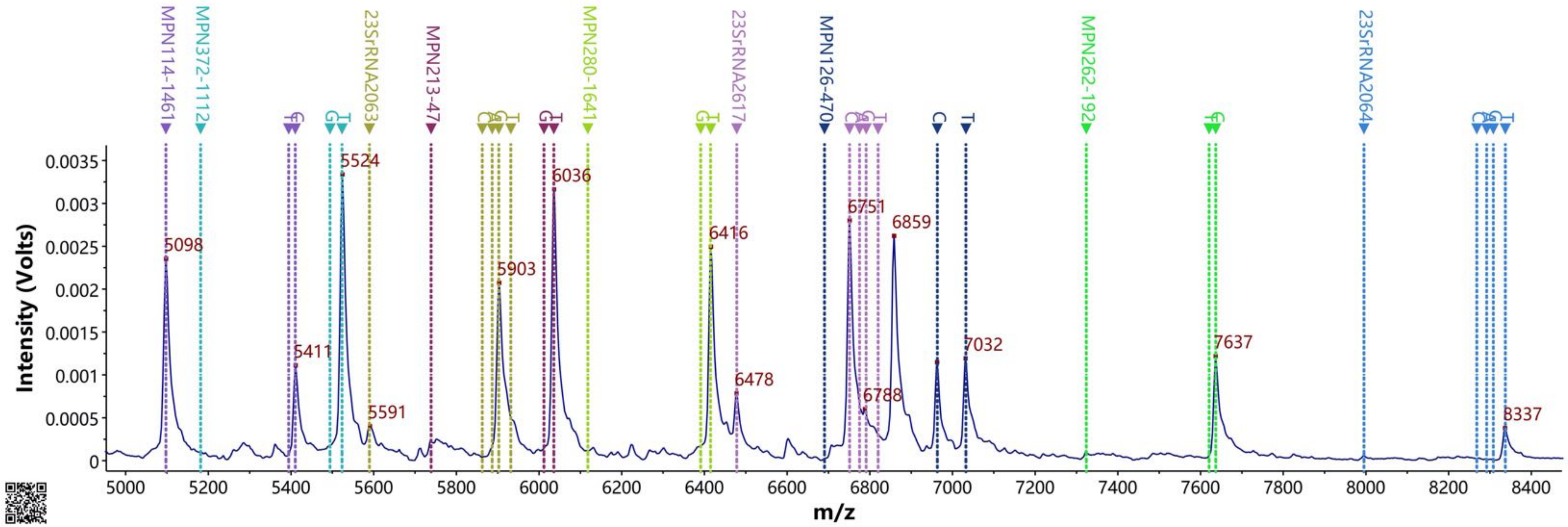

MPN114-1461
MPN372-1112
23SrRNA2063
MPN213-47
MPN280-1641
23SrRNA2617
MPN126-470
MPN262-192
23SrRNA2064
5098
5411
5524
5591
5903
6036
6416
6478
6751
6788
6859
7032
7637
8337
Intensity (Volts)
m/z

Sample-49

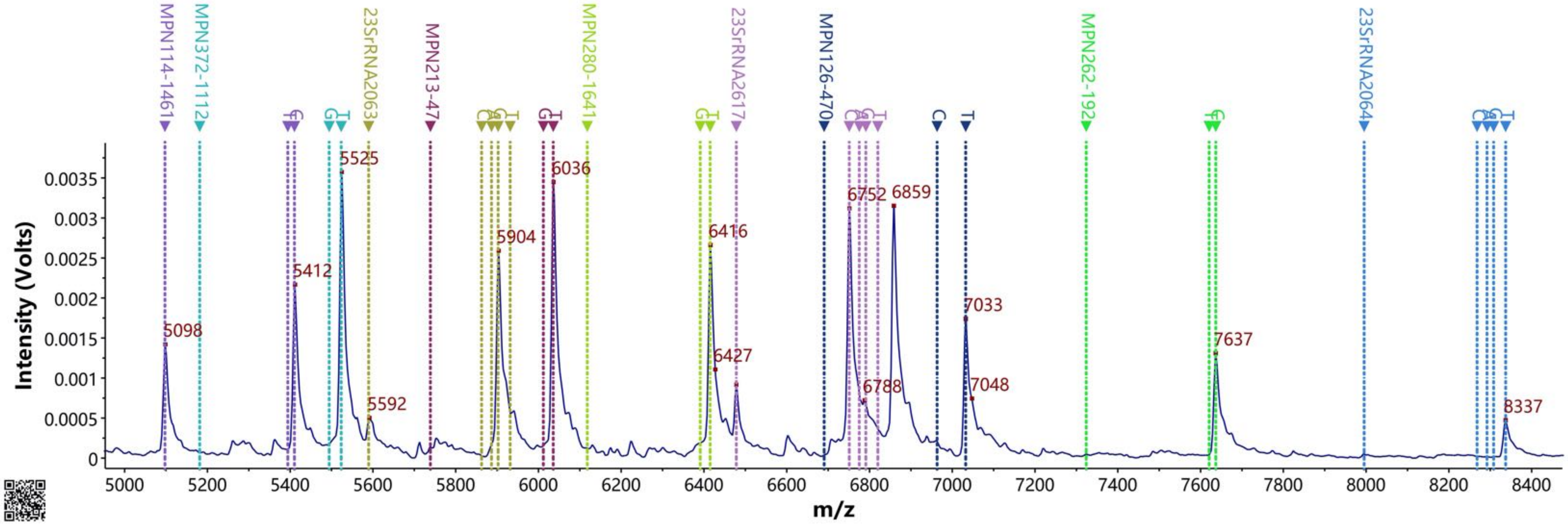

MPN114-1461
MPN372-1112
23SrRNA2063
MPN213-47
MPN280-1641
23SrRNA2617
MPN126-470
MPN262-192
23SrRNA2064
5098
5412
5525
5592
5904
6036
6416
6427
6752
6788
6859
7033
7048
7637
8337
Intensity (Volts)
m/z

Sample-50

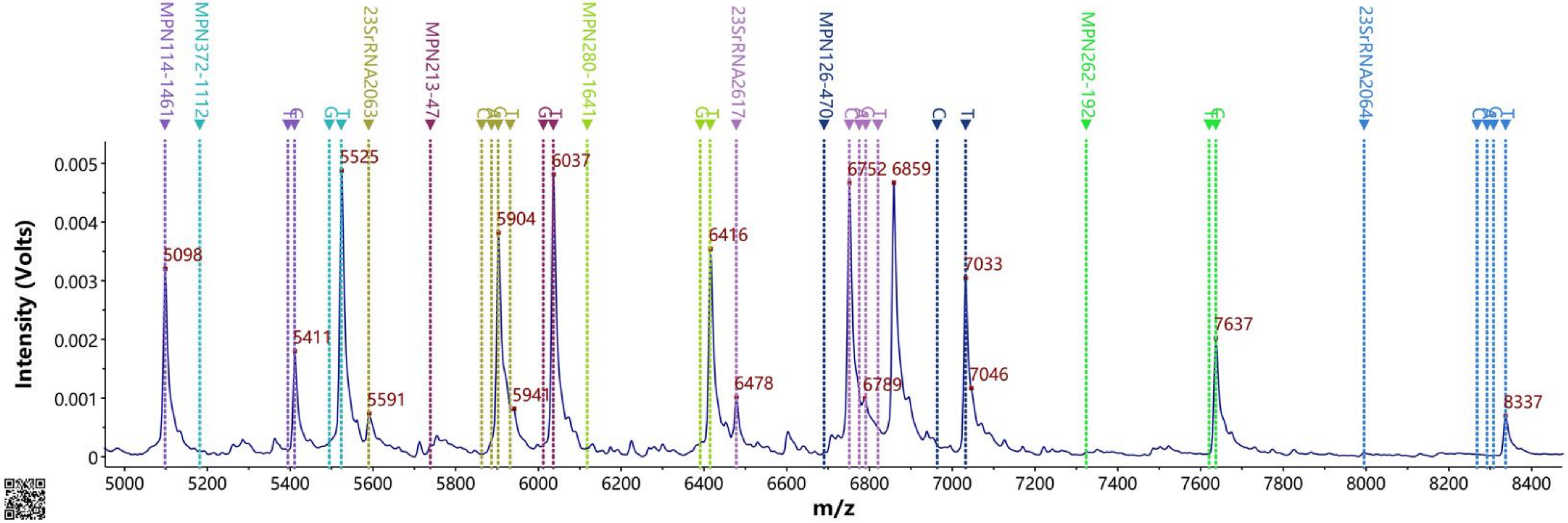
MPN114-1461
MPN372-1112
23SrRNA2063
MPN213-47
MPN280-1641
23SrRNA2617
MPN126-470
MPN262-192
23SrRNA2064
5098
5411
5525
5591
5904
5941
6037
6416
6478
6752
6789
6859
7033
7046
7637
8337
Intensity (Volts)
m/z

Sample-51

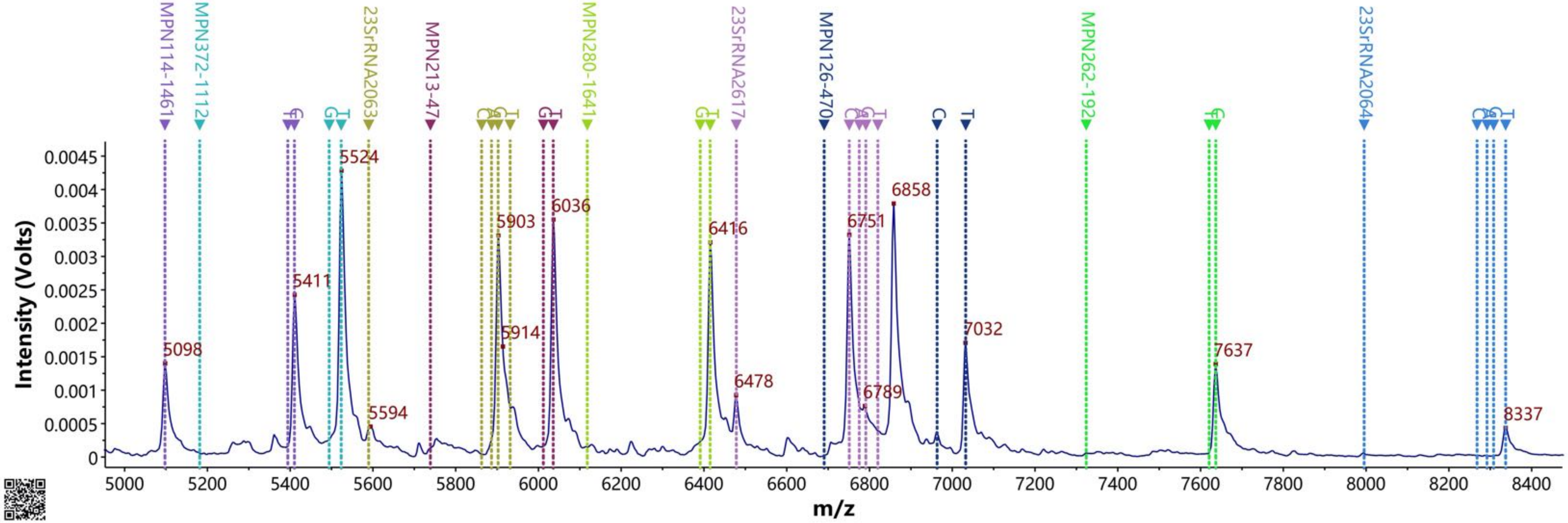

MPN114-1461
MPN372-1112
23SrRNA2063
MPN213-47
MPN280-1641
23SrRNA2617
MPN126-470
MPN262-192
23SrRNA2064
5098
5411
5524
5594
5903
5914
6036
6416
6478
6751
6789
6858
7032
7637
8337
Intensity (Volts)
m/z

Sample-52

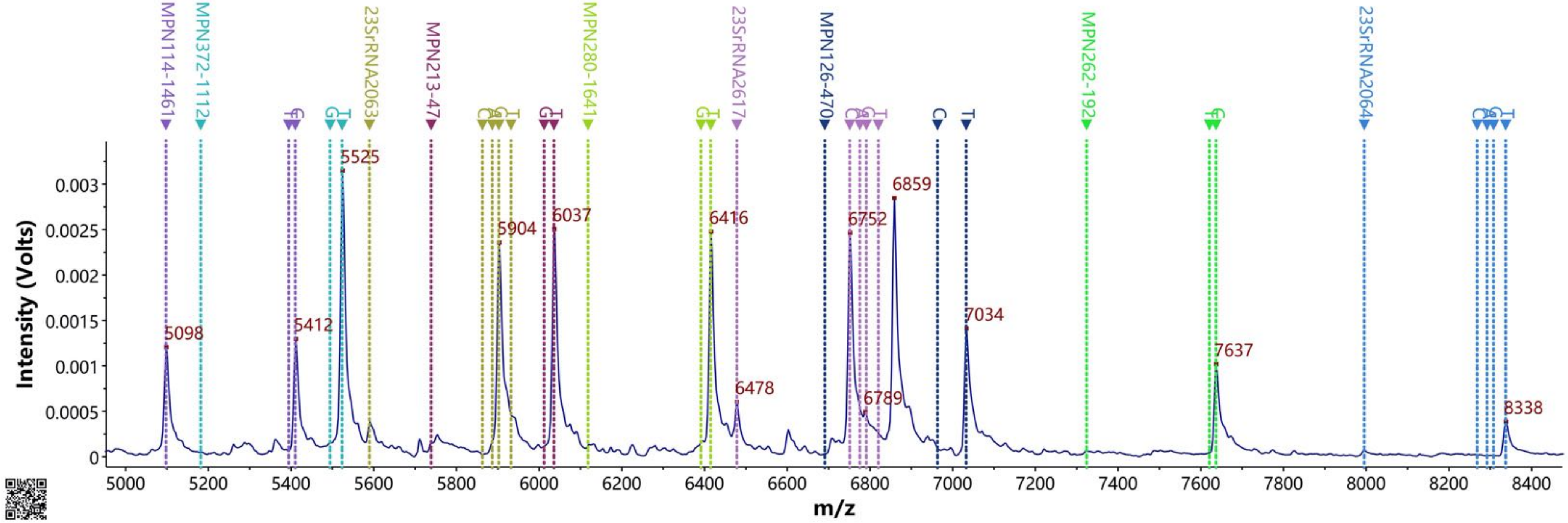

MPN114-1461
MPN372-1112
23SrRNA2063
MPN213-47
MPN280-1641
23SrRNA2617
MPN126-470
MPN262-192
23SrRNA2064
5098
5412
5525
5904
6037
6416
6478
6752
6789
6859
7034
7637
8338
Intensity (Volts)
m/z

Sample-53

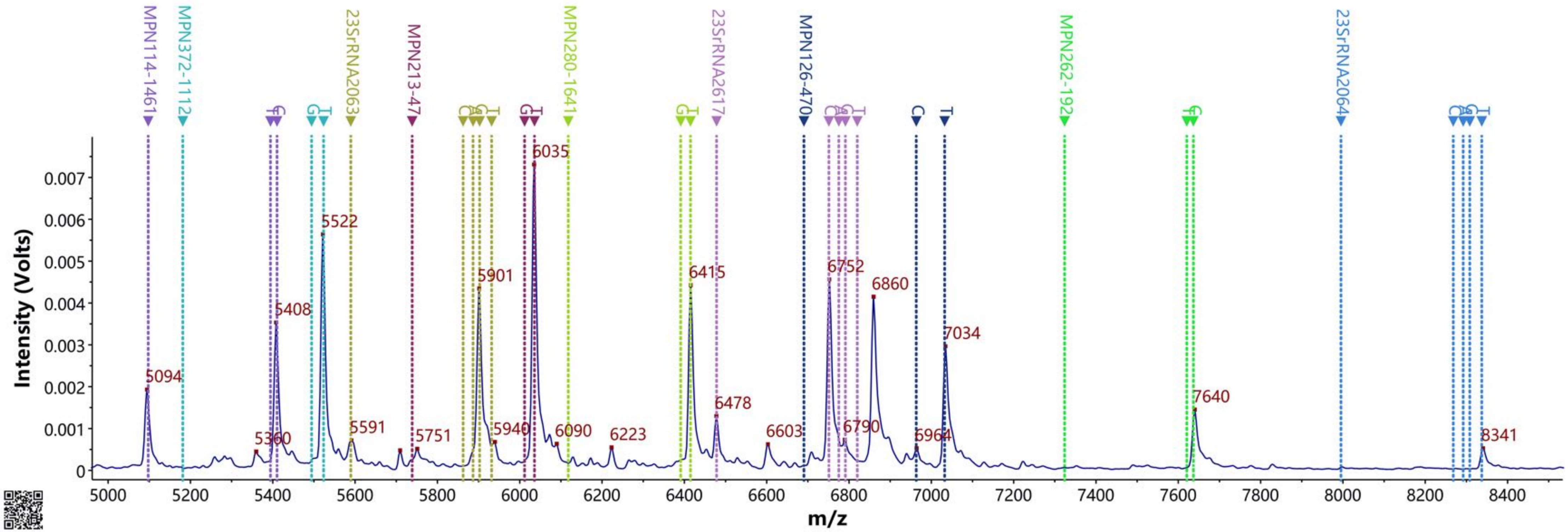

MPN114-1461
MPN372-1112
23SrRNA2063
MPN213-47
MPN280-1641
23SrRNA2617
MPN126-470
MPN262-192
23SrRNA2064
5094
5360
5408
5522
5591
5751
5901
5940
6035
6090
6223
6415
6478
6603
6752
6790
6860
6964
7034
7640
8341
Intensity (Volts)
m/z

Sample-54

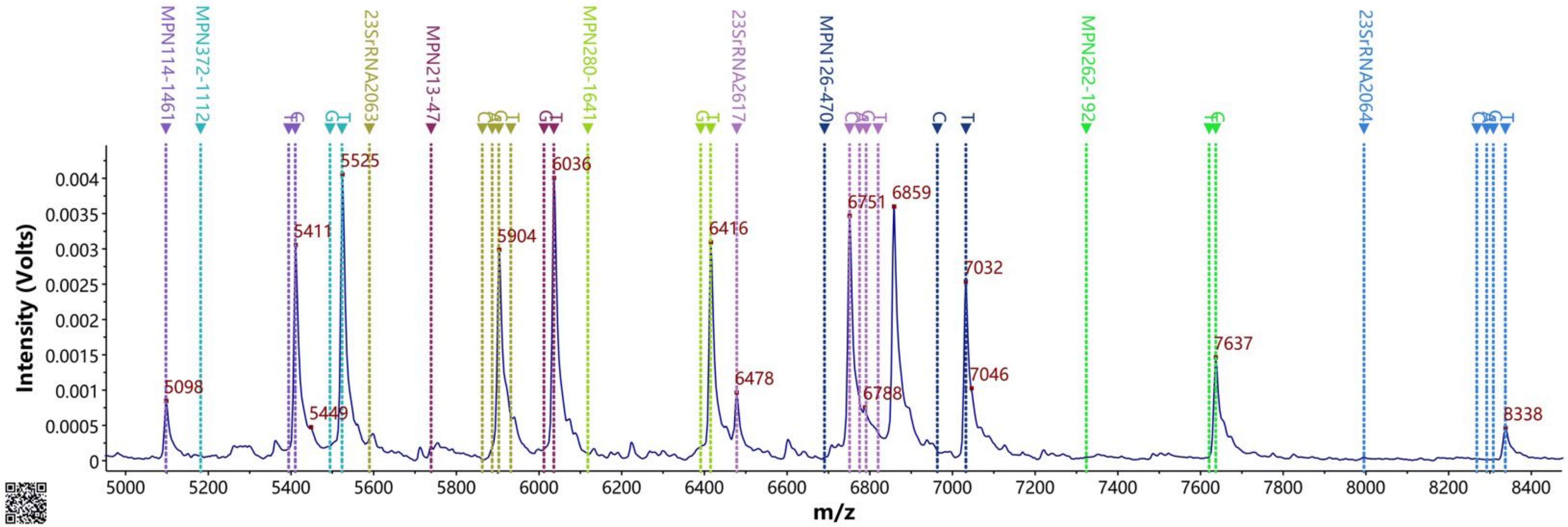

MPN114-1461
MPN372-1112
23SrRNA2063
MPN213-47
MPN280-1641
23SrRNA2617
MPN126-470
MPN262-192
23SrRNA2064
5098
5411
5449
5525
5904
6036
6416
6478
6751
6788
6859
7032
7046
7637
8338
Intensity (Volts)
m/z

Sample-55

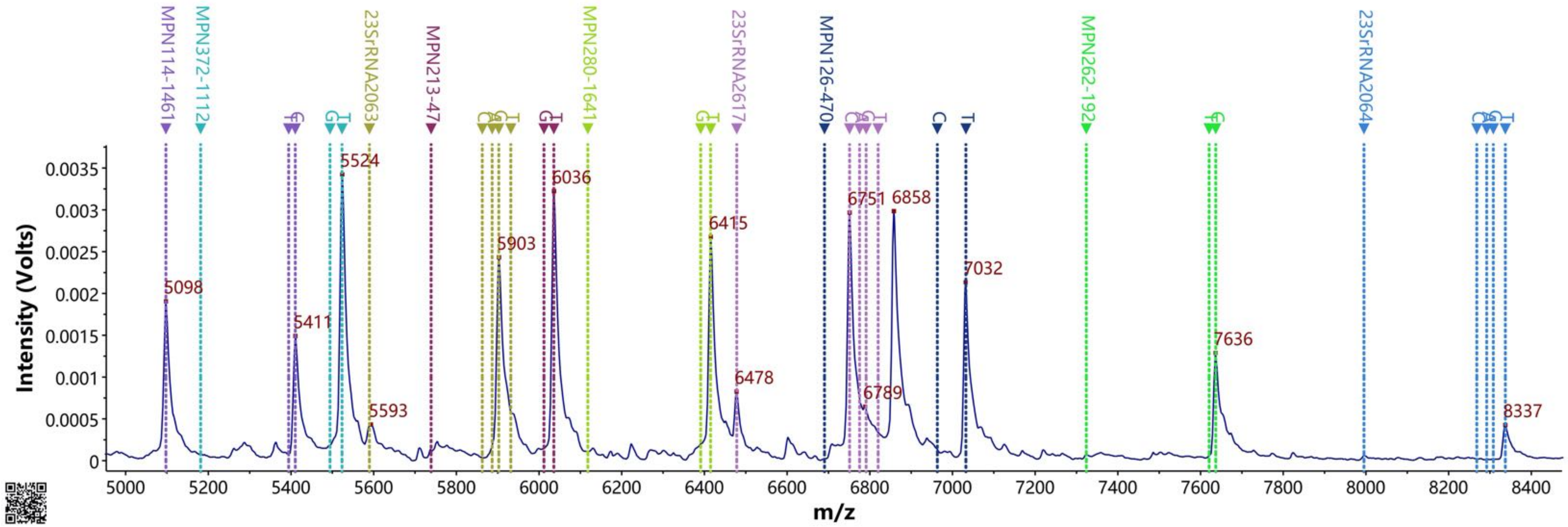
MPN114-1461
MPN372-1112
23SrRNA2063
MPN213-47
MPN280-1641
23SrRNA2617
MPN126-470
MPN262-192
23SrRNA2064
5098
5411
5524
5593
5903
6036
6415
6478
6751
6789
6858
7032
7636
8337
Intensity (Volts)
m/z

Sample-56

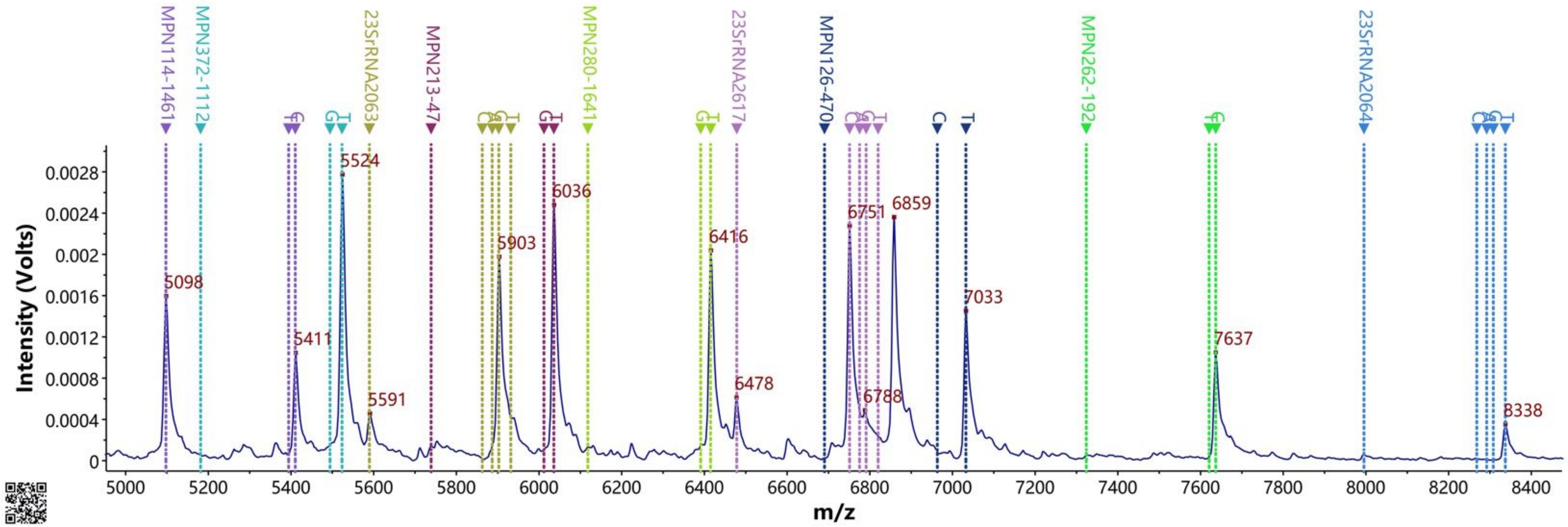
MPN114-1461
MPN372-1112
23SrRNA2063
MPN213-47
MPN280-1641
23SrRNA2617
MPN126-470
MPN262-192
23SrRNA2064
5098
5411
5524
5591
5903
6036
6416
6478
6751
6788
6859
7033
7637
8338
Intensity (Volts)
m/z

Sample-57

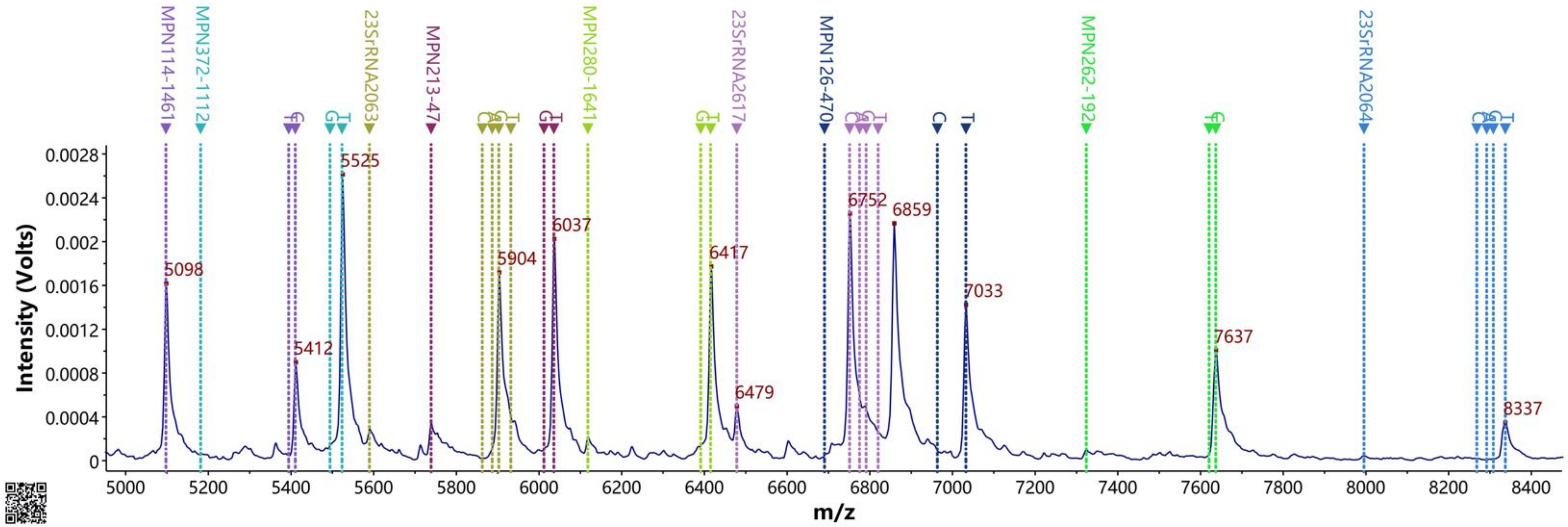

MPN114-1461
MPN372-1112
23SrRNA2063
MPN213-47
MPN280-1641
23SrRNA2617
MPN126-470
MPN262-192
23SrRNA2064
5098
5412
5525
5904
6037
6417
6479
6752
6859
7033
7637
8337
Intensity (Volts)
m/z

Sample-58

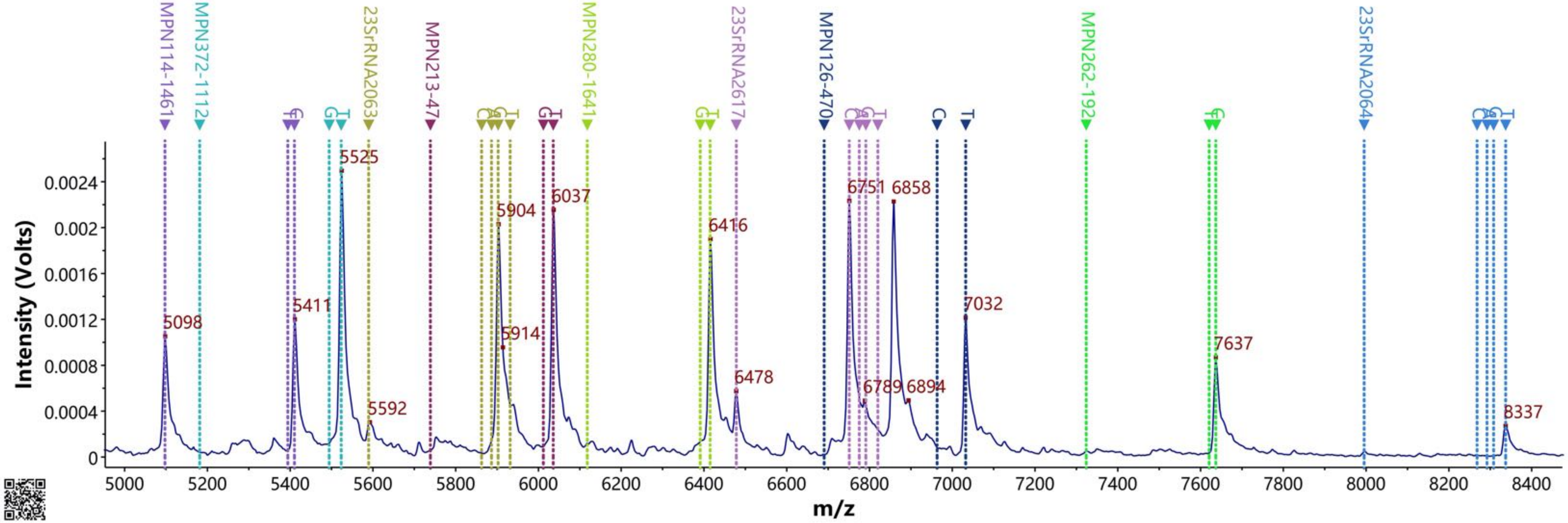

MPN114-1461
MPN372-1112
23SrRNA2063
MPN213-47
MPN280-1641
23SrRNA2617
MPN126-470
MPN262-192
23SrRNA2064
5098
5411
5525
5592
5904
5914
6037
6416
6478
6751
6789
6858
6894
7032
7637
8337
Intensity (Volts)
m/z

Sample-59

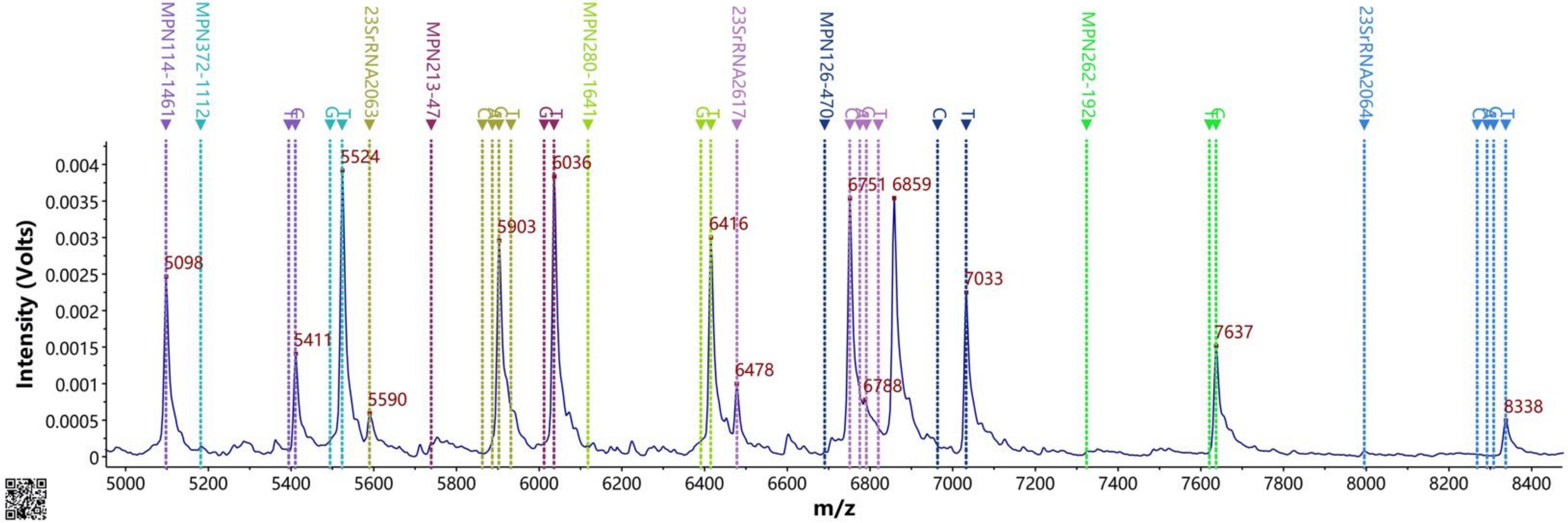

MPN114-1461
MPN372-1112
23SrRNA2063
MPN213-47
MPN280-1641
23SrRNA2617
MPN126-470
MPN262-192
23SrRNA2064
5098
5411
5524
5590
5903
6036
6416
6478
6751
6788
6859
7033
7637
8338
Intensity (Volts)
m/z

Sample-60

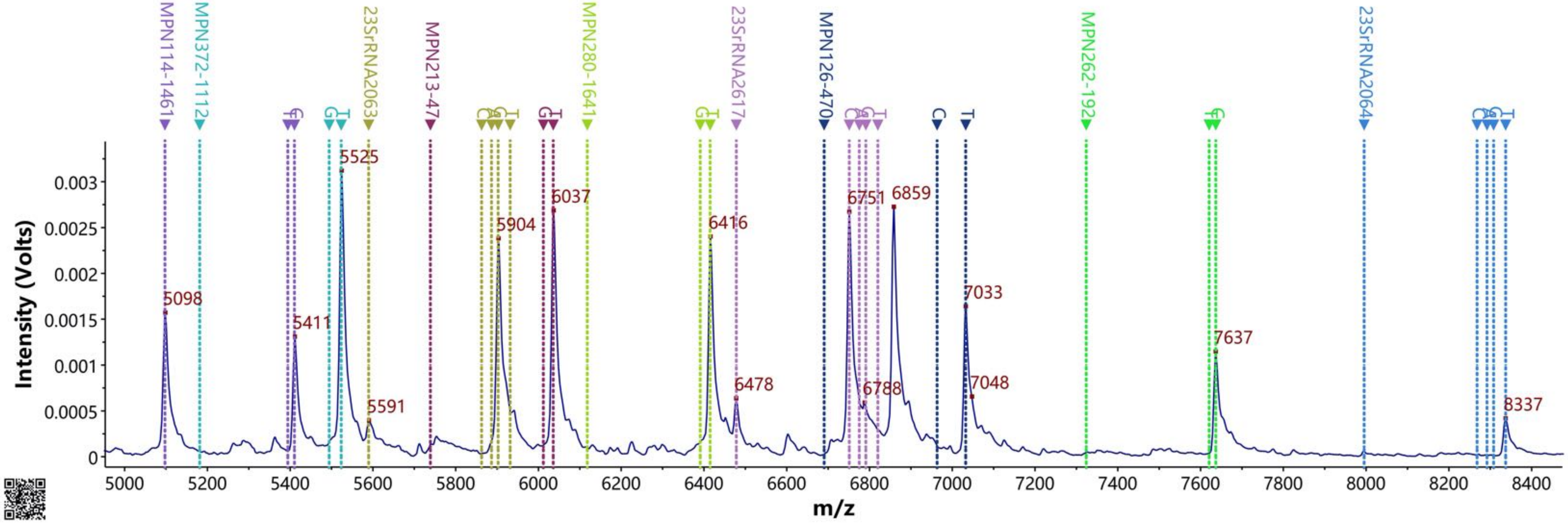
MPN114-1461
MPN372-1112
23SrRNA2063
MPN213-47
MPN280-1641
23SrRNA2617
MPN126-470
MPN262-192
23SrRNA2064
5098
5411
5525
5591
5904
6037
6416
6478
6751
6788
6859
7033
7048
7637
8337
Intensity (Volts)
m/z

Sample-61

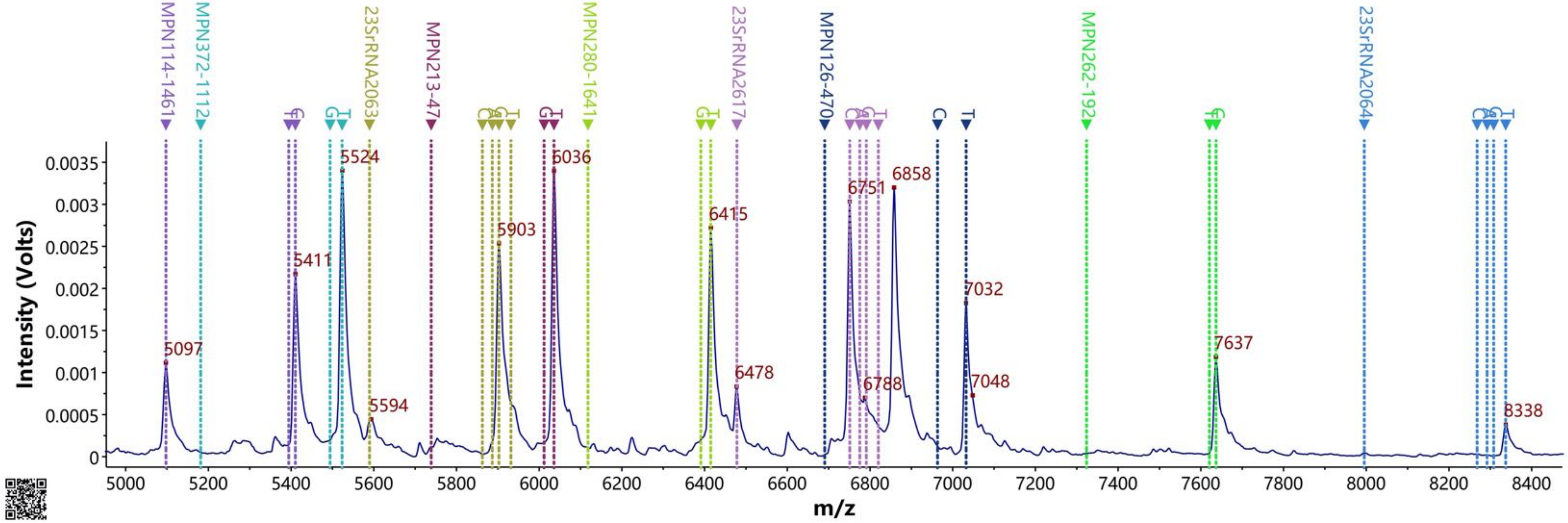
MPN114-1461
MPN372-1112
23SrRNA2063
MPN213-47
MPN280-1641
23SrRNA2617
MPN126-470
MPN262-192
23SrRNA2064
5097
5411
5524
5594
5903
6036
6415
6478
6751
6788
6858
7032
7048
7637
8338
Intensity (Volts)
m/z

Sample-62

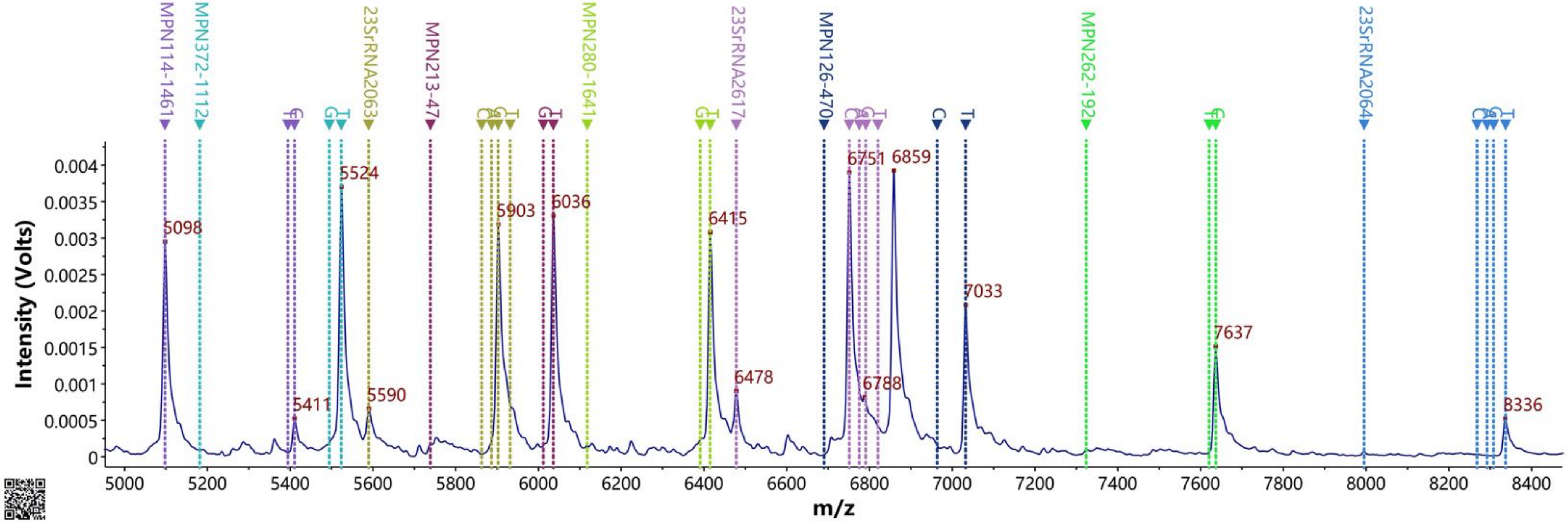

MPN114-1461
MPN372-1112
23SrRNA2063
MPN213-47
MPN280-1641
23SrRNA2617
MPN126-470
MPN262-192
23SrRNA2064
5098
5411
5524
5590
5903
6036
6415
6478
6751
6788
6859
7033
7637
8336
Intensity (Volts)
m/z

Sample-63

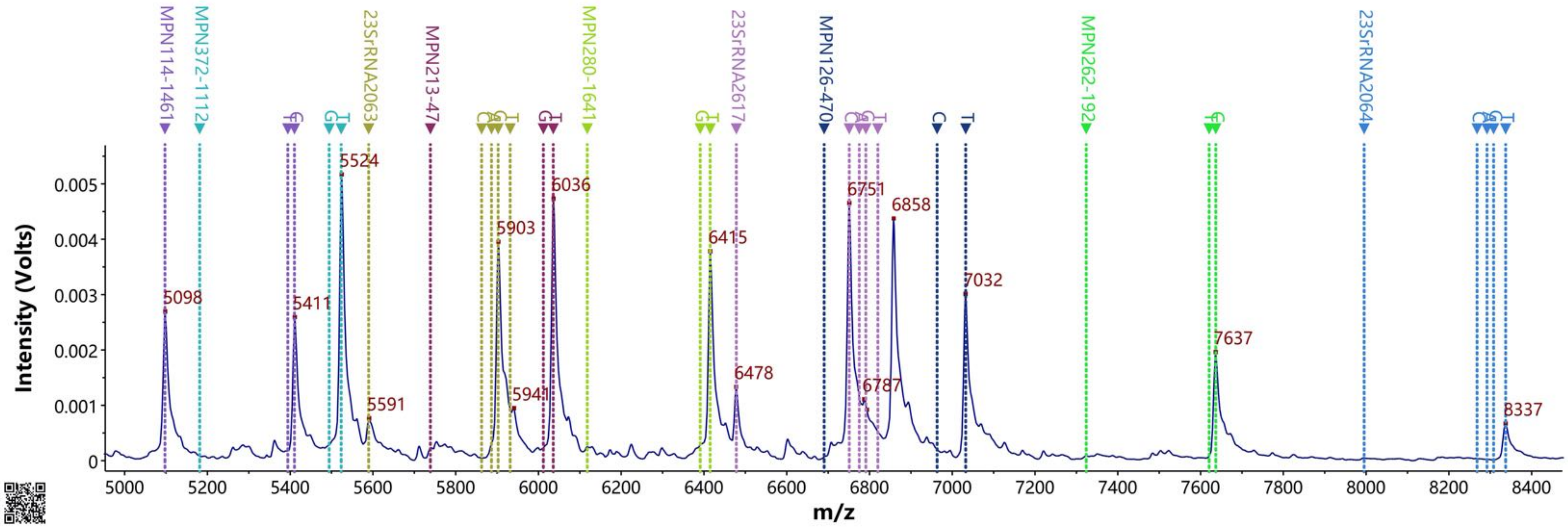

MPN114-1461
MPN372-1112
23SrRNA2063
MPN213-47
MPN280-1641
23SrRNA2617
MPN126-470
MPN262-192
23SrRNA2064
5098
5411
5524
5591
5903
5941
6036
6415
6478
6751
6787
6858
7032
7637
8337
Intensity (Volts)
m/z

Sample-64

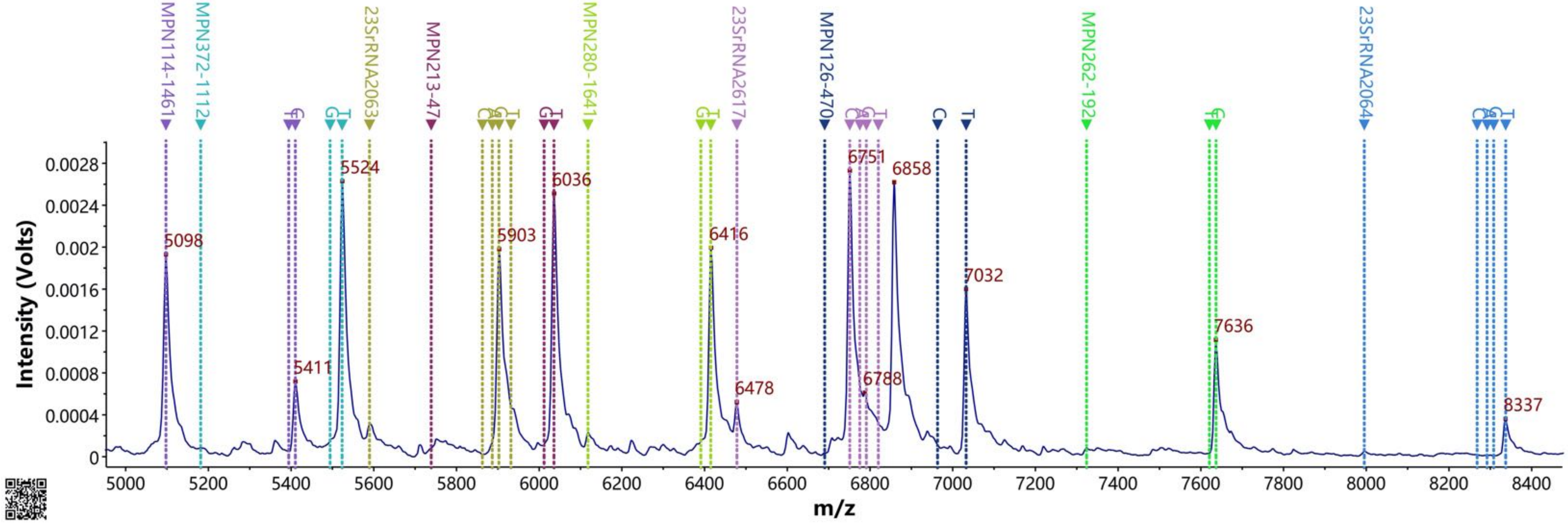

MPN114-1461
MPN372-1112
23SrRNA2063
MPN213-47
MPN280-1641
23SrRNA2617
MPN126-470
MPN262-192
23SrRNA2064
5098
5411
5524
5903
6036
6416
6478
6751
6788
6858
7032
7636
8337
Intensity (Volts)
m/z

Sample-65

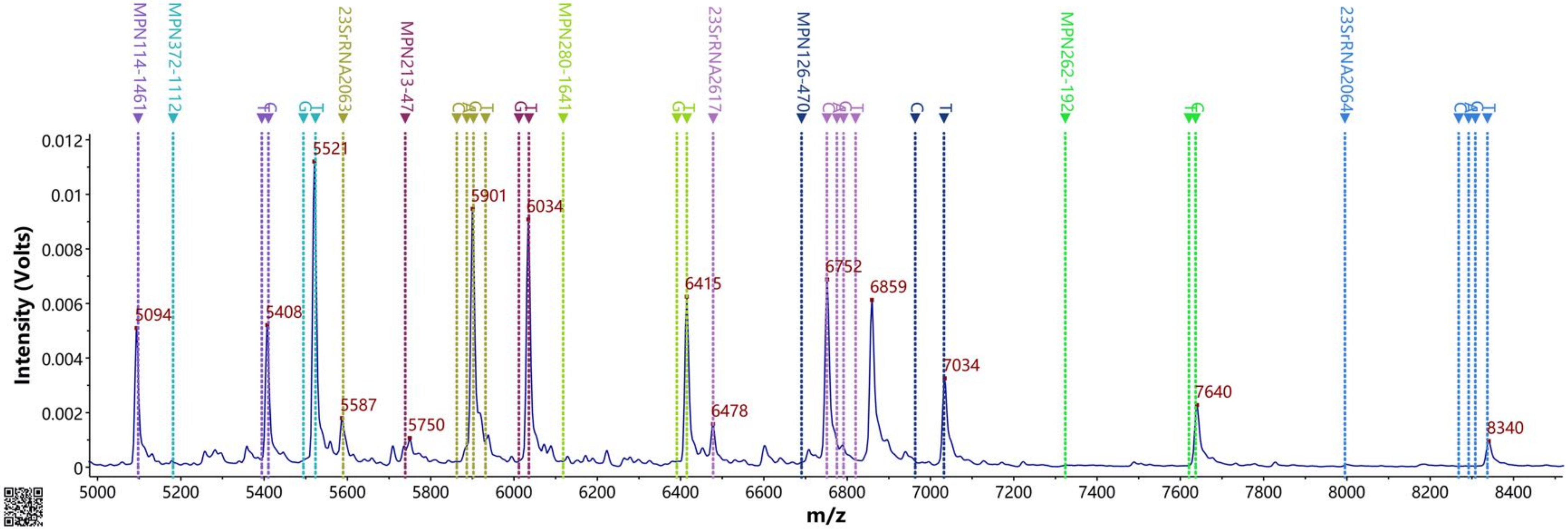

MPN114-1461
MPN372-1112
23SrRNA2063
MPN213-47
MPN280-1641
23SrRNA2617
MPN126-470
MPN262-192
23SrRNA2064
5094
5408
5521
5587
5750
5901
6034
6415
6478
6752
6859
7034
7640
8340
Intensity (Volts)
m/z

Sample-66

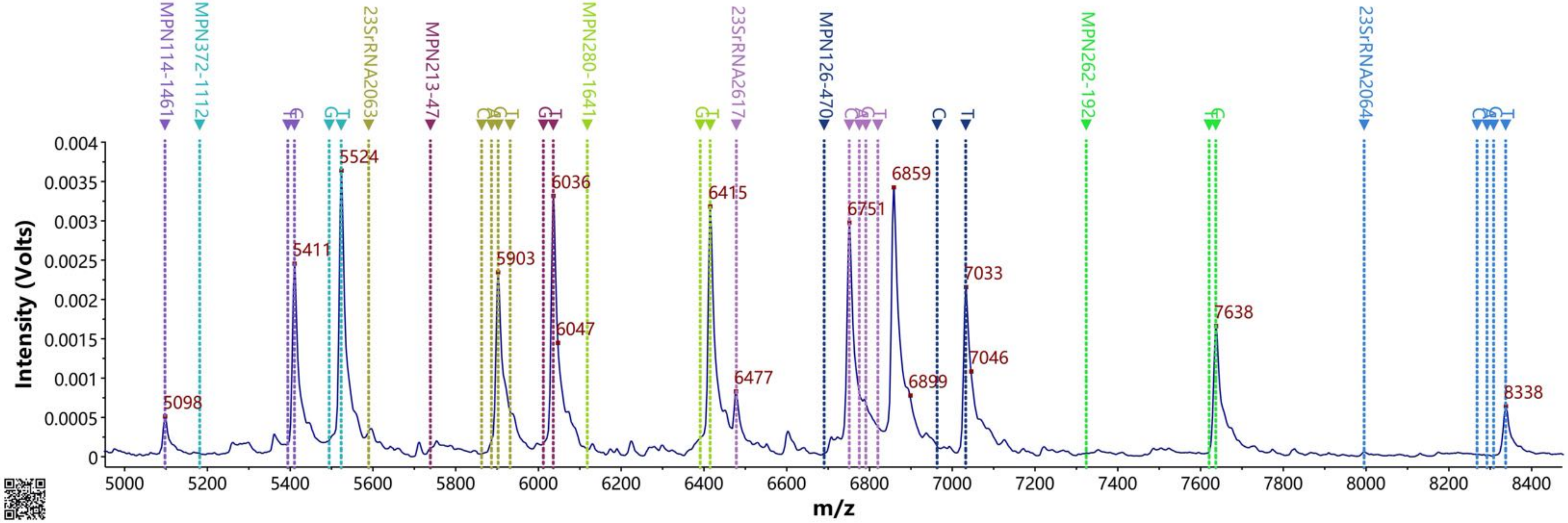

MPN114-1461
MPN372-1112
23SrRNA2063
MPN213-47
MPN280-1641
23SrRNA2617
MPN126-470
MPN262-192
23SrRNA2064
5098
5411
5524
5903
6036
6047
6415
6477
6751
6859
6899
7033
7046
7638
8338
Intensity (Volts)
m/z

Sample-67

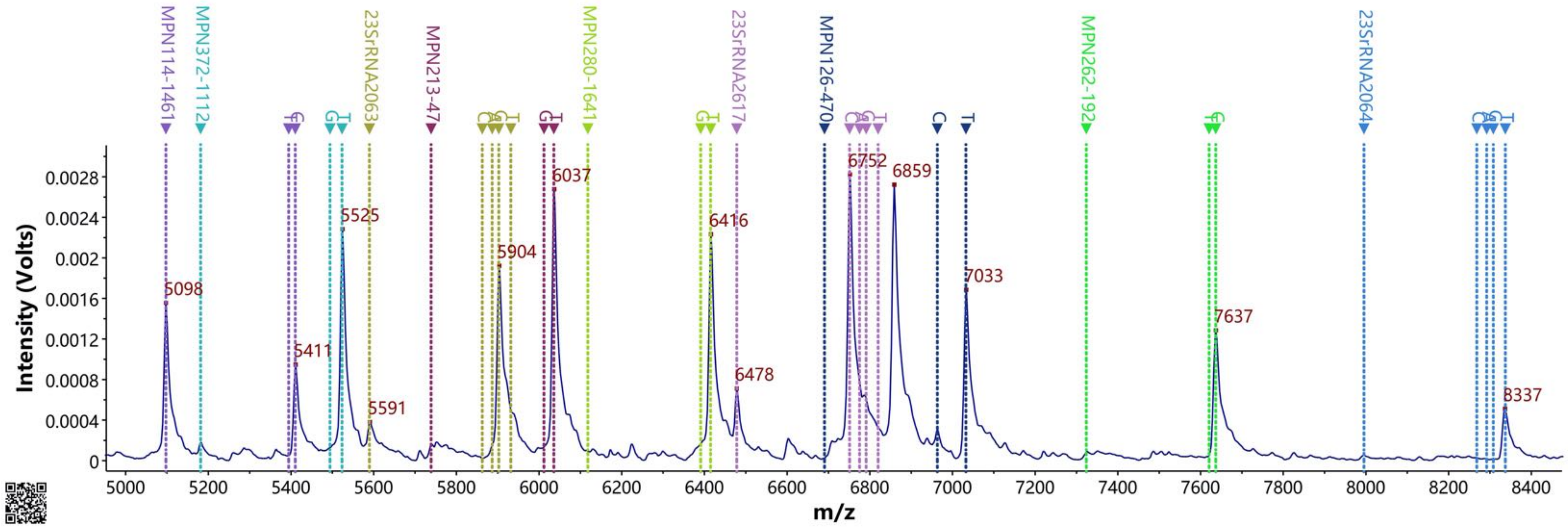

MPN114-1461
MPN372-1112
23SrRNA2063
MPN213-47
MPN280-1641
23SrRNA2617
MPN126-470
MPN262-192
23SrRNA2064
5098
5411
5525
5591
5904
6037
6416
6478
6752
6859
7033
7637
8337
Intensity (Volts)
m/z

Sample-68

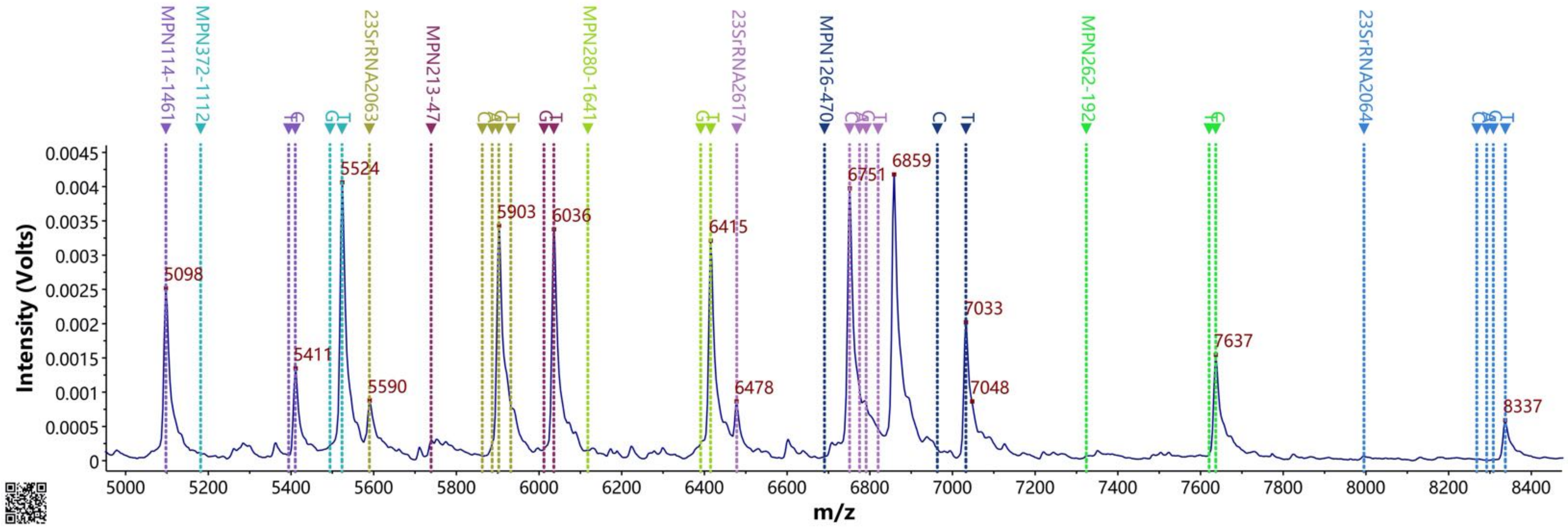

MPN114-1461
MPN372-1112
23SrRNA2063
MPN213-47
MPN280-1641
23SrRNA2617
MPN126-470
MPN262-192
23SrRNA2064
5098
5411
5524
5590
5903
6036
6415
6478
6751
6859
7033
7048
7637
8337
Intensity (Volts)
m/z

Sample-69

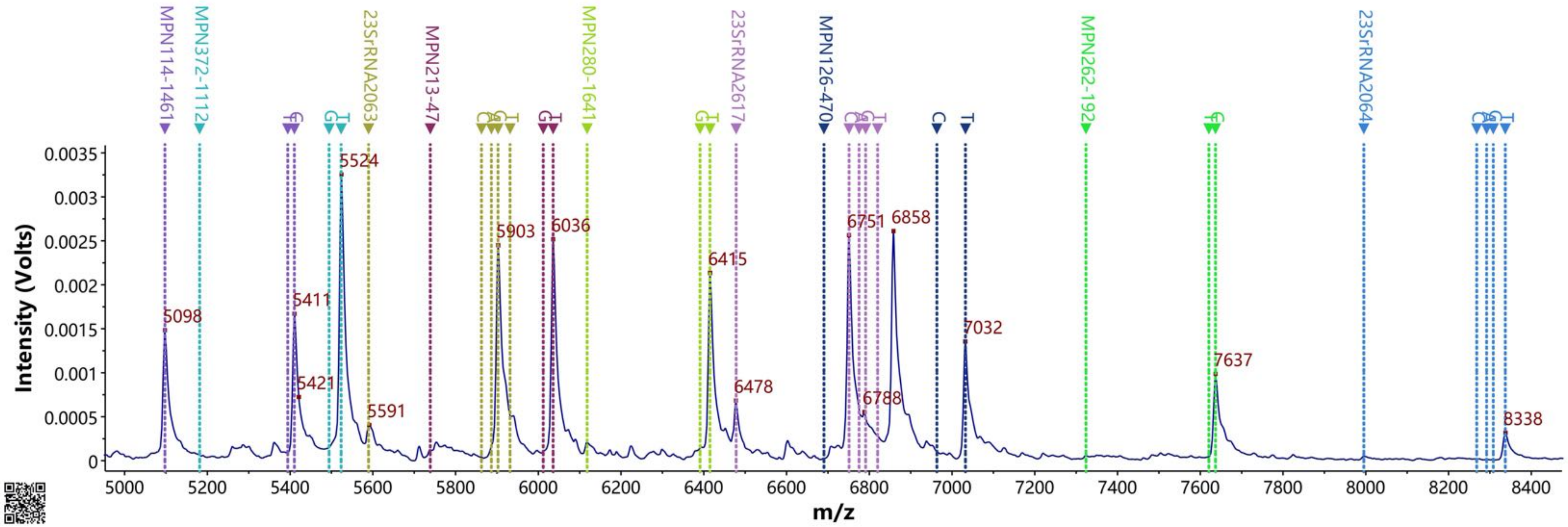
MPN114-1461
MPN372-1112
23SrRNA2063
MPN213-47
MPN280-1641
23SrRNA2617
MPN126-470
MPN262-192
23SrRNA2064
5098
5411
5421
5524
5591
5903
6036
6415
6478
6751
6788
6858
7032
7637
8338
Intensity (Volts)
m/z

Sample-70

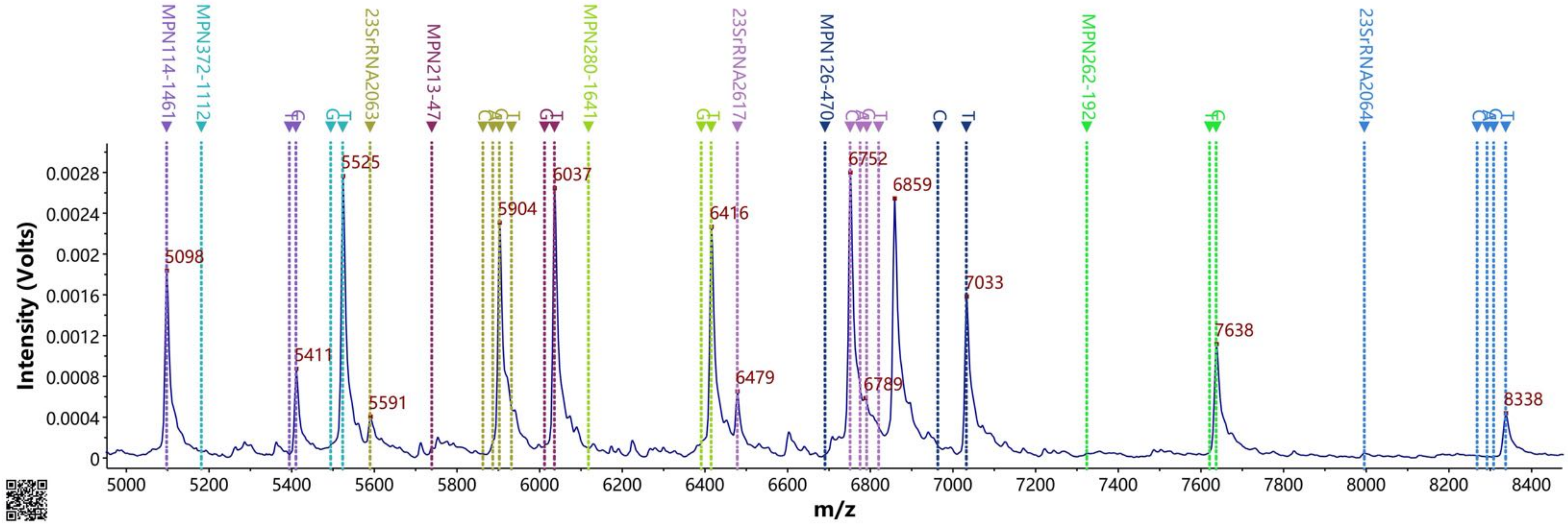

MPN114-1461
MPN372-1112
23SrRNA2063
MPN213-47
MPN280-1641
23SrRNA2617
MPN126-470
MPN262-192
23SrRNA2064
5098
5411
5525
5591
5904
6037
6416
6479
6752
6789
6859
7033
7638
8338
Intensity (Volts)
m/z
